# Metabolomic profiling reveals correlations between spermiogram parameters and the metabolites present in human spermatozoa and seminal plasma

**DOI:** 10.1371/journal.pone.0211679

**Published:** 2019-02-20

**Authors:** Kathrin M. Engel, Sven Baumann, Ulrike Rolle-Kampczyk, Jürgen Schiller, Martin von Bergen, Sonja Grunewald

**Affiliations:** 1 Training Center of the European Academy of Andrology (EAA), Dermatology, Venerology and Allergology Clinic, University Hospital Leipzig, Leipzig, Germany; 2 Institute of Medical Physics and Biophysics, Faculty of Medicine, University of Leipzig, Leipzig, Germany; 3 Department of Molecular Systems Biology, Helmholtz Centre for Environmental Research – UFZ, Leipzig, Germany; 4 Institute of Biochemistry, Faculty of Life Sciences, University of Leipzig, Leipzig, Germany; ENEA Centro Ricerche Casaccia, ITALY

## Abstract

In 50% of all infertility cases, the male is subfertile or infertile, however, the underlying mechanisms are often unknown. Even when assisted reproductive procedures such as *in vitro* fertilization and intracytoplasmic sperm injection are performed, the causes of male factor infertility frequently remain elusive. Since the overall activity of cells is closely linked to their metabolic capacity, we analyzed a panel of 180 metabolites in human sperm and seminal plasma and elucidated their associations with spermiogram parameters. Therefore, metabolites from a group of 20 healthy donors were investigated using a targeted LC-MS/MS approach. The correlation analyses of the amino acids, biogenic amines, acylcarnitines, lysophosphatidylcholines, phosphatidylcholines, sphingomyelins and sugars from sperm and seminal plasma with standard spermiogram parameters revealed that metabolites in sperm are closely related to sperm motility, whereas those in seminal plasma are closely related to sperm concentration and morphology. This study provides essential insights into the metabolome of human sperm and seminal plasma and its associations with sperm functions. This metabolomics technique could be a promising screening tool to detect the factors of male infertility in cases where the cause of infertility is unclear.

## Introduction

Fertility problems are increasingly occurring in couples worldwide, and male factors are involved in approximately 50% of all infertility cases [1]. To predict male reproductive potential and record treatment success, clinicians currently primarily rely on semen analyses conducted according to the guidelines of the World Health Organization (WHO) [2]. This protocol comprises the determination of rather superficial parameters, such as semen volume, semen pH, sperm cell count, sperm motility, and sperm morphology. With the exception of motility these parameters do not allow the detailed assessment of the functional status of the sperm cell. Therefore, the causes of male factor infertility frequently remain elusive even when assisted reproductive procedures such as *in vitro* fertilization (IVF) and intracytoplasmic sperm injection (ICSI) are performed.

New technologies, including genomics, proteomics and, most recently, metabolomics, have spurred the search for novel male infertility biomarkers [3]. In this context, the detection of genes, proteins [4], or metabolites unique to the infertile male might lead to an improved understanding of male sub- and infertility. This approach would also permit the identification of an accurate set of biomarkers that could be used to diagnose such patients with increased sensitivity and specificity.

At present, very limited data on the metabolome of healthy human semen are available. For human seminal plasma, some data on metabolites are available in the literature [5,6]. The correlation analysis of the spermiogram parameters obtained by a computer-assisted sperm analysis (CASA) and sperm metabolites have also been reported [6]. In human sperm, 42 and 27 metabolites have been identified by untargeted approaches using nuclear magnetic resonance (NMR) spectroscopy and gas chromatography coupled to mass spectrometry (GC-MS), respectively [7]. MS-based methods were also used to characterize the lipid composition of sperm and seminal plasma from different species [8–10]. However, to the authors’ best knowledge, a targeted approach to identify metabolites present in sperm and seminal plasma obtained from the same donors by liquid chromatography (LC) and the determination of the correlation of the levels of these metabolites in both types of specimens with manually assessed spermiogram parameters have not yet been reported.

Sperm cell lysis is challenging [11] due to the small cell size, proteins that are almost completely bound to the cytoskeleton, high compartmentalization and limited cytoplasm. In this study, we established reliable protocols for the investigation of the metabolome and the identification of potential metabolomic candidates in the sperm and seminal plasma obtained from 20 healthy donors. The total concentrations of amino acids, biogenic amines, acylcarnitines, lysophosphatidylcholines, phosphatidylcholines, sphingomyelins and the sum of hexoses were determined, and studies correlating the levels of these metabolites with standard spermiogram parameters were performed.

## Materials and methods

### Semen specimens

The study was approved by the Institutional Review Board of the University of Leipzig (No. 136-10-31052010) considering the Declaration of Helsinki. All patients signed a written informed consent form. Semen samples were collected from 20 healthy donors (age (mean ± SD) 31.7 ± 6.8 years, body mass index (BMI) 22.4 ± 2.2, abstention time 3.6 ± 1.1 days). The measured semen parameters of all of the samples were within the reference ranges defined by the World Health Organization for the normal fertile population [2].

### Inclusion and exclusion criteria

Donors were invited to take part in the present study if they were 20 to 45 years old, normal weight (BMI 19 to 24), and did not suffer from any metabolic or cardiovascular diseases. Donors were informed that they should stay abstinent for 2 to up to 7 days. These criteria were confirmed by signing the written informed consent form.

Semen samples were excluded from the study if the sperm concentration was < 15 × 10^6^ cells/ml and/or if sperm total motility (progressive + local motility) was < 40%.

### Manual evaluation of sperm motility and sperm morphology

The motility of sperm in a sample volume of 10 μl was evaluated microscopically at 400× magnification. Sperm were classified as progressively motile, locally motile or immotile as suggested by the WHO (WHO, 2010).

For the assessment of sperm morphology, smears were air-dried and stained according to the modified Papanicolaou staining method using Orange G 6, Mayer’s Hemalum solution (Dr. K. Hollborn & Söhne GmbH & Co. KG, Leipzig, Germany) and EA-50 (Sigma-Aldrich Chemie GmbH, Taufkirchen, Germany). The morphology of 200 spermatozoa was determined under a light microscope at 1,000× magnification using an oil-immersion objective [12]. The head, neck, midpiece and tail of sperm cells were evaluated according to the WHO laboratory handbook [2].

The characteristics of the study group and the determined spermiogram parameters are shown in S1 Table.

### Density gradient centrifugation

The liquefied semen samples were subjected to density gradient centrifugation (ISolate sperm separation medium, Irvine Scientific, Santa Ana, CA, USA). In brief, the samples were loaded onto a 50% and 90% discontinuous gradient of sperm separation medium, and centrifuged at 600×g for 20 min at room temperature (22°C). The supernatant, which consisted of the seminal plasma, was collected in a separate tube and stored at -80°C until further processing. The 90% pellet, which represented the mature sperm fraction, was resuspended in phosphate-buffered saline (PBS, pH 7.4). The sperm cells present in this fraction were then counted using a Neubauer hemocytometer. After an additional centrifugation of the re-suspended mature sperm fraction for 5 min at 5,300×g and 4°C, the supernatant was discarded.

### Metabolic profiling of healthy semen donors

The following protocol was established to ensure a valid analytical procedure. All samples were treated in a standardized manner. The preanalytical period was kept as short as possible.

The metabolomic analysis of human sperm and seminal plasma was conducted using the AbsoluteIDQ p180 kit (Biocrates Life Sciences AG, Innsbruck, Austria) as previously described [13]. All samples were prepared and analyzed according to the protocol provided by Biocrates. Briefly, sperm metabolites were extracted by adding 2 ml methanol/water (1:1, v/v) to the sperm pellet and subjecting it to ultrasonic homogenization for 2 min on ice; this resulted in sperm cell lysis. The samples were centrifuged for 5 min at 5,300×g and 4°C, and the supernatants were treated according to the protocol provided by the manufacturer.

The amino acids and biogenic amines were analyzed by liquid chromatography (LC)-mass spectrometry (MS) (LC-MS/MS). Acylcarnitines, phosphatidylcholines (including lysophosphatidylcholines), sphingomyelins and hexoses were analyzed by flow injection analysis (FIA)-MS/MS measurements (FIA-MS/MS). Samples were pipetted into the wells of a 96-well sandwich filter plate, which included stable isotope-labeled internal standards. The filters were dried under a stream of nitrogen, the amino acids in the sample were derivatized with 5% phenylisothiocyanate reagent (PITC), and the filters were dried again. After the extraction of the metabolites and internal standards with 300 μl 5 mM ammonium acetate in methanol and shaking at 450 rpm (Thermomixer comfort, Eppendorf, Hamburg, Germany) for 30 min at room temperature, the solution was centrifuged through a solid-phase filter membrane and diluted for further analysis.

All analyses were performed on a QTRAP mass spectrometer under electrospray ionization (ESI) (ABI Sciex API 5500 Q-TRAP). The MS was coupled to an ultra-performance liquid chromatography (UPLC) system (Waters Acquity, Waters Corporation, Milford, CT, USA). Metabolite detection was achieved by positive and negative multiple reaction monitoring (MRM) using an injection volume of 20 μl for FIA-MS/MS and an injection volume of 10 μl for LC-MS/MS. For calibration, a calibration mixture consisting of 7 different concentrations was used. Quality controls obtained from lyophilized human plasma samples were included at 3 different concentration levels.

Raw data were collected in Analyst software (Sciex, Framingham, MA, USA) and processed using MetIDQ software, which is an integrated part of the p180 Kit (Biocrates). The quantification of LC-MS/MS data was performed using the IntelliQuan algorithm in Analyst software (Sciex). Subsequently, the determined metabolite concentrations of each sample were normalized to the amount per 100 × 10^6^ sperm cells.

In general, 171 out of 180 metabolites (present as reference compounds in the kit) could be detected in sperm, and 177 out of 180 metabolites were present in seminal plasma. Only those metabolites that were present in ≥75% of the samples were used in further analyses. In detail, the aspartate (Asp), citrulline (Cit), glycine (Gly), histidine (His), lysine (Lys), methionine (Met), ornithine (Orn), tryptophan (Trp), valine (Val) and etherphosphorylcholine (GPCe) 42:4 levels in sperm cells and the acetylornithine (Ac-Orn), phenethylamine (PEA), phosphatidylcholine (PC) 30:2 and PC 38:1 levels in seminal plasma were not included in the correlation analyses.

For a summary of the study please see S1 Fig.

### Statistical analysis

Statistical analyses were performed using GraphPad Prism 6 (GraphPad Software, Inc., La Jolla, CA, USA). The mean values and standard deviations of the concentrations of all metabolites were calculated. The data were analyzed for normal distribution and logarithmic normal distribution (if data were not normally distributed) using the D’Agostino & Pearson omnibus normality test. Because not all data were normally distributed, Spearman correlation coefficients (*r*_*s*_) were calculated. All tests were nonparametric and two-tailed, and significance was indicated by *P* < 0.05.

## Results and discussion

Despite the standardization of the experimental procedure, high deviations were observed for some analytes (S2 Table), possibly as a result of interindividual differences. The normalization to 100 × 10^6^ sperm cells could also contribute to the deviations because the concentrations of "0" remain unaffected by this normalization. However, this normalization is less error-prone than dilution of the samples or the attempt to concentrate the samples to 100 × 10^6^ sperm cells prior to MS analysis. The results of the seminal plasma analysis are more reliable than those of the sperm analysis.

The differences in the metabolite concentrations between sperm and seminal plasma indicate proper sample preparation and negligible mixing of the sperm and seminal plasma. Additionally, it can be assumed that sperm lysis occurred only after the separation of the sperm from the seminal plasma.

### Amino acids and biogenic amines

Mature sperm are transcriptionally and translationally inert [14,15]. The proteins present in sperm are synthesized by the precursor cells during spermatogenesis.

As expected, all proteinogenic amino acids could be extracted from human sperm cells and seminal plasma. The presence of some amino acids, namely, alanine (Ala), arginine (Arg), glutamine (Gln), Gly, serine (Ser), tyrosine (Tyr), Val, leucine (Leu), isoleucine (Ile) and threonine (Thr) in human sperm has already been shown by NMR spectroscopic and GC-MS-based approaches [7].

**Fig 1:**
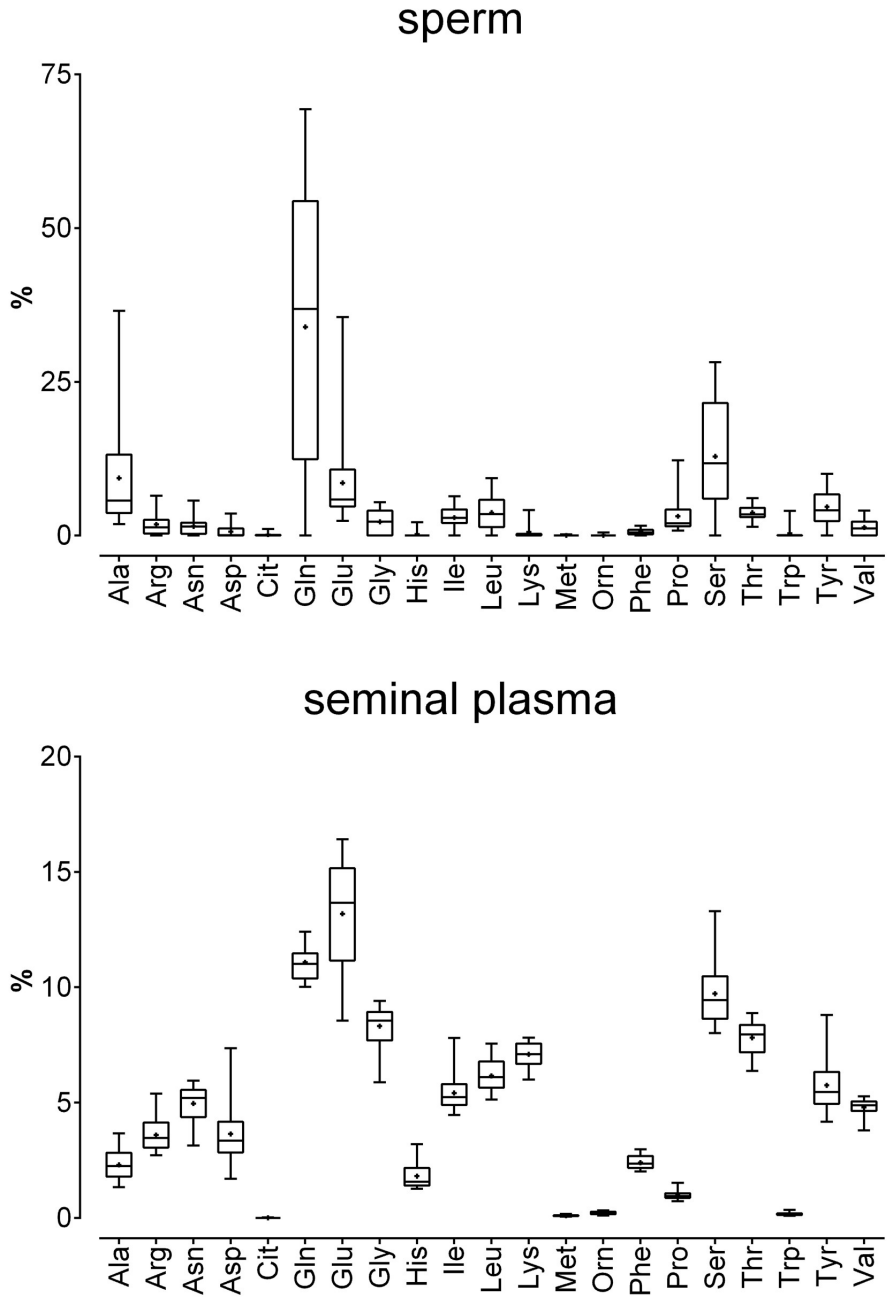
Relative amino acid concentrations in sperm and seminal plasma obtained from 20 healthy human donors. The box plots show the minimum, maximum and mean (black cross) of the data. Relative amounts were calculated for each sample based on the total amount of amino acids in the sample and are given in %. Total concentrations (in μmol) are given in the S2 Table.

In the present study the total amount of amino acids present in seminal plasma was higher than that in sperm, with a mean 800-fold increase. However, the total amino acid content of sperm and seminal plasma correlated positively with sperm count (*r*_*s*_ = 0.535 and 0.544, respectively). The distribution pattern of the proteinogenic amino acids in sperm and in seminal plasma differed. As shown in Fig 1 and S2 Table, although the nonessential amino acid Gln was the most abundant amino acid in both specimens, in absolute counts, it was far more abundant in sperm than in seminal plasma (mean ± SD 33.9 ± 23.8% vs. 11.1 ± 0.7%). The high Gln content of sperm proteins is likely due to the presence of the androgen receptor, which contains polyglutamine stretches (CAG repeats) in its transactivation domain [16,17].

Previously published metabolomic analyses [18–20] reported that Gln is also present in high concentrations in human blood plasma and serum. In the study of Breier and colleagues the mean relative amounts of Gln measured in 22 subjects (approximately 21% for blood plasma and serum) were higher than the relative amounts found in seminal plasma but lower than the relative amounts found in sperm in the present study (Fig 1).

In contrast, not all of the investigated nonproteinogenic amino acids and biogenic amines could be detected in human sperm and seminal plasma. Whereas asymmetrically dimethylated arginine (ADMA), Cit, kynurenine, Orn, putrescine, sarcosine, serotonin, spermidine, spermine, taurine and total dimethylamine (total DMA) were present in both types of specimens, Ac-Orn, α-aminoadipic acid (alpha-AAA), carnosine, creatinine, L-4-hydroxyproline (OH-Pro) and PEA were detected only in seminal plasma. Histamine, methionine sulfoxide (Met-SO) and 3-nitrotyrosine (Nitro-Tyr) were found neither in sperm nor in seminal plasma (S2 Table). This is in contrast to Paiva *et al*. [7] and Zhao *et al*. [21], who also found alpha-AAA, creatinine and PEA in human sperm cells, but with different techniques, namely, NMR spectroscopy or GC.

### Correlations among amino acids and biogenic amines

**Fig 2:**
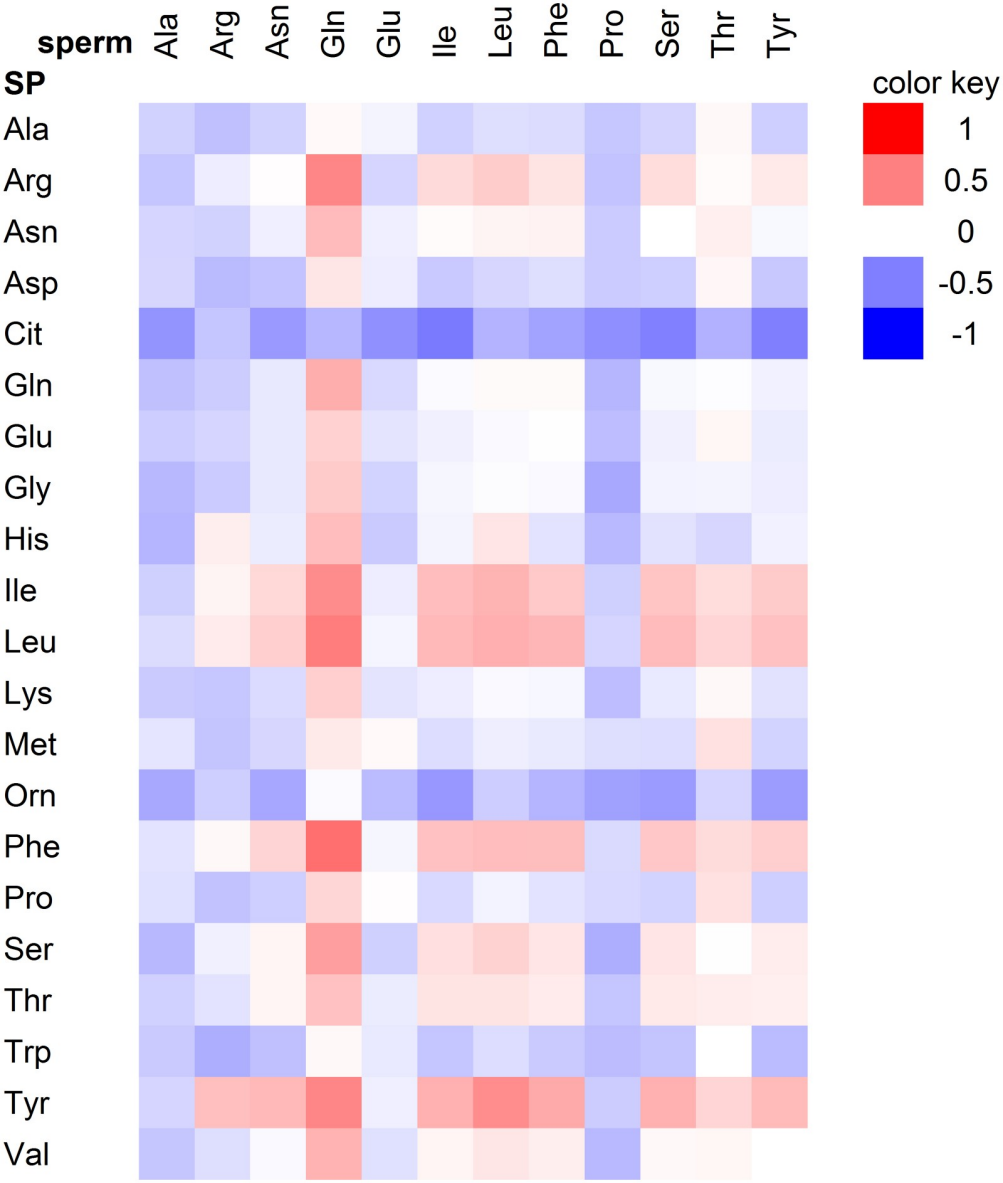
Heat map of the correlation coefficients between levels of amino acids from ejaculated mature human sperm and seminal plasma. Red colored cells represent positive correlations, and blue colored cells represent negative correlations. Only metabolites that were present in ≥ 75% of the samples were considered for correlation analyses.

The quantities of most amino acids in sperm and seminal plasma were correlated in a significantly positive manner (S3 and S4 Tables). In contrast, the amounts of most individual amino acids in sperm and seminal plasma did not show cross-correlation (Fig 2). Only a few statistically significant associations were found between the levels of specific amino acids in sperm and those in seminal plasma. Among those, Gln in sperm showed a significant positive correlation (*P* < 0.05) with Arg (*r*_*s*_ = 0.477), Ile (0.454), Leu(0.512), phenylalanine (Phe, 0.568) and Tyr (0.476) in seminal plasma. A significant positive correlation was also found for Leu in sperm with Tyr (0.448) in seminal plasma (Fig 2, S5 Table).

### Correlations between amino acid and biogenic amine concentrations and spermiogram parameters

**Fig 3:**
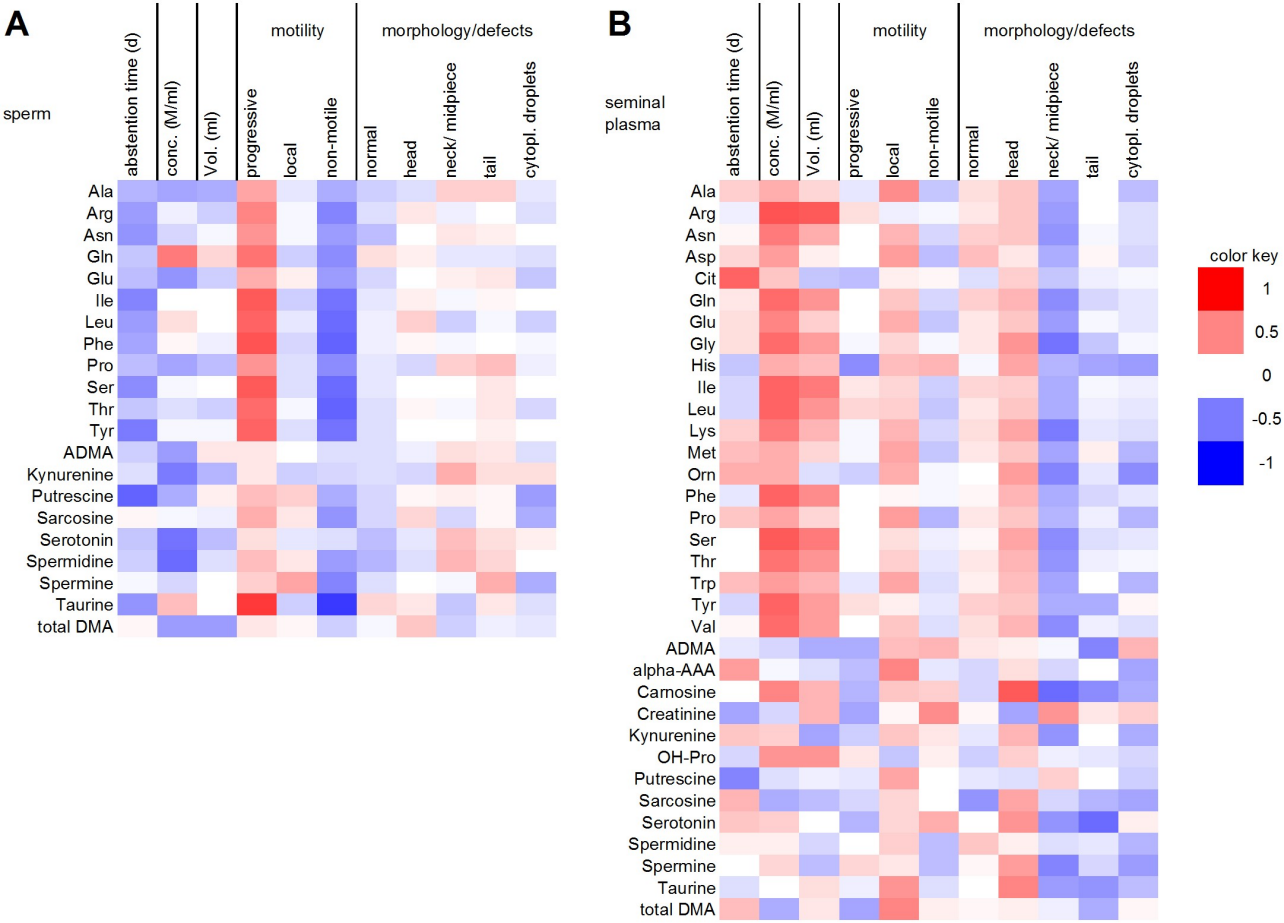
Heat map of the correlation coefficients between spermiogram parameters and the levels of amino acids and biogenic amines from ejaculated mature human sperm (A) and seminal plasma (B). Red colored cells represent positive correlations, blue colored cells represent negative correlations. Only metabolites that were present in ≥ 75% of the samples were included in the correlation analyses.

The concentrations of some amino acids and biogenic amines **in sperm** were correlated with spermiogram parameters (Fig 3A and S6 Table). In detail, abstention time tended to correlate negatively with amino acid content, and the correlation was significant (*P* < 0.05) for Ile (*r*_*s*_ = -0.478), Ser (-0.456), Tyr (-0.523) and putrescine (-0.598). Certain amino acids showed a positive correlation with progressive motility of sperm. This correlation was significant for Arg (0.499), Gln (0.562), Ile (0.629), Leu (0.603), Phe (0.674), Ser (0.639) and Tyr (0.622). NMR- and GC-MS-based studies found that the concentrations of some amino acids of sperm cells are changed significantly in asthenozoospermic patients where the motility of sperm is severely reduced. In accordance with our study the Leu levels were decreased in these patients [21].

Previous publications have described Arg as a crucial component in spermatogenesis. Diets deficient in Arg lead to the presence of multinuclear giant cells in the testis and to the impairment of spermatogenesis in men [22]. It was also shown that following the consumption of an Arg-deficient diet, the number and motility of human sperm decreased dramatically [23]. In contrast, Arg supplementation in men and boars increased the number and motility of sperm [24,25].

Gln and Arg, Gln and Ser, as well as Phe and Tyr, are connected via their synthesis pathways. The Ser and Tyr residues in proteins are potential sites for protein phosphorylation, and such sites have been shown to undergo crucial modifications during signal transduction cascades involved in sperm motility [26–29]. Additionally, several proteins involved in energy metabolism, protein folding/degradation, vesicle trafficking and cytoskeleton function have been reported to be associated with sperm motility [30–32]. The highest and most significant correlation was found between the sperm concentration of taurine and progressive motility (*r*_*s*_ = 0.784, *P* = 0.0001). This finding is consistent with the results of earlier studies in which it was shown that the addition of taurine to the medium improves the sperm motility in various species [33,34].

Although the sperm concentration of ejaculates tended to be negatively associated with their amino acid and biogenic amine content (significant for kynurenine (-0.526), serotonin (-0.545) and spermidine (-0.585)), a significant positive correlation was found for Gln (0.530).

No significant correlations between sperm morphology and the amino acid or biogenic amine content of the sperm were found.

The **seminal plasma** content of most proteinogenic amino acids and carnosine was significantly positively correlated with sperm concentration (*P* < 0.05). This result is in accordance with Qiao *et al*., who also showed a positive correlation of the concentrations of some amino acids detected by GC-MS with sperm concentration [6]. Nonproteinogenic amino acids and other biogenic amines showed no association with sperm concentration (Fig 3B and S7 Table). Accordingly, the amount of all proteinogenic amino acids in seminal plasma also tended to be positively correlated with the volume of the ejaculate, reaching statistical significance for Arg, Ile, Phe and Ser (*r*_*s*_ = 0.642, 0.524, 0.469 and 0.528, respectively). The levels of nonproteinogenic amino acids and biogenic amines showed no clear relationship to the volume of the ejaculate.

High creatinine levels in seminal plasma were significantly positively correlated with the amount of immotile sperm (*P* < 0.05). This finding is in accordance with an NMR-based study that showed increased creatinine levels in the seminal plasma of patients with asthenozoospermia [35]. Creatinine, a degradation product of creatine, is one of the most abundant biogenic amines in human blood serum [20] and is cleared via the kidneys [36]. Therefore, the creatinine in seminal plasma could result from a contamination of the ejaculate with urine. In stallions, urine contamination of ejaculates has been shown to lead to a significant reduction in total and progressive sperm motility [37]. Furthermore, there are reports of elevated creatine kinase activity in subfertile patients [38,39].

The inverse correlation of the putrescine concentration in sperm with abstention time is consistent with the observation of a similar negative correlation in seminal plasma. Putrescine is a basic, polycationic product of Arg degradation and is further converted into spermidine and spermine. In sperm and seminal plasma, the concentrations of spermine were tenfold higher than those of spermidine. A high abundance of spermine was also found in earlier studies in which seminal plasma was shown to contain the highest spermine concentration of all body fluids [40]. The concentrations of putrescine, spermidine and spermine tended to be inversely correlated with sperm immotility. This is consistent with reports in oligospermic men showing that treatment with S-adenosylmethionine, a polyamine precursor, leads to an increase in polyamine content and improved sperm motility [41]. Furthermore, compared to that of normal donors, higher concentrations of spermine and spermidine were reported in the seminal plasma of obese men (BMI > 30) with a poor spermiogram [42]. Low concentrations of spermine and ornithine (a precursor of putrescine) in seminal plasma were associated with higher frequencies of neck/midpiece defects in sperm cells. Spermine has been described as a decapacitation factor for sperm that ensures the proper timing of the acrosome reaction and capacitation [43].

Components of the serotonin (5-HT) system have been shown to be present in human ejaculate. The observed inverse correlation of the serotonin concentration in sperm with the sperm concentration is consistent with the results of former studies in which it was shown that hyperserotonemia leads to azoospermia [44] and that increased serotonin concentration in the spermatic vein is correlated with significantly lower sperm quality [45]. Impaired semen quality has also been associated with the use of serotonin reuptake inhibitors, which block serotonin receptors and thereby lead to an increase in serotonin concentration [46,47].

Normal sperm morphology was not significantly correlated with the levels of any amino acids or biogenic amines in seminal plasma. Interestingly, in-depth morphological analysis revealed significant positive correlations of the carnosine and taurine content of seminal plasma with the occurrence of head defects and significant negative correlations of Gln, Gly, Lys, Orn, Ser, carnosine and spermine (*P* < 0.05; *r*_*s*_ = -0.444, -0.538, -0.498, -0.484, -0.454, -0.576 and -0.473, respectively) levels with neck and midpiece defects in sperm cells. Tail defects were significantly negatively correlated (*P* < 0.05) with seminal plasma levels of ADMA (*r*_*s*_ = -0.488), carnosine (-0.446) and serotonin (-0.590). The presence of cytoplasmic droplets was inversely associated with Orn levels in seminal plasma (-0.457).

### Acylcarnitines

**Fig 4:**
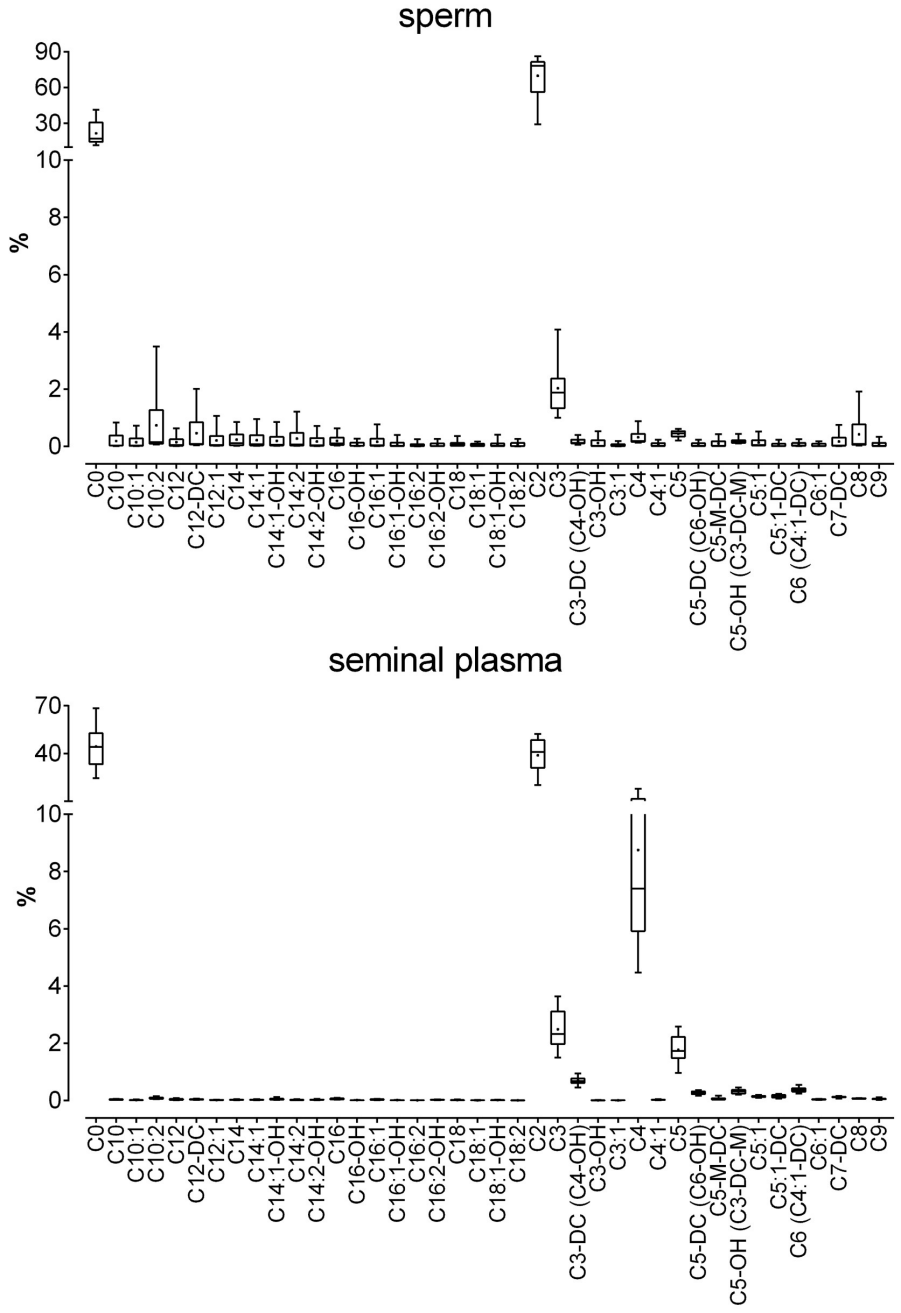
Relative acylcarnitine concentrations in sperm and seminal plasma obtained from 20 healthy human donors. The box plots show the minimum, maximum and mean (black cross) values of the data. The relative amounts were calculated for each sample based on the total amount of acylcarnitines in the sample and are given in %. Total concentrations (in μmol) are given in S2 Table.

Among acylcarnitines, DL-carnitine (C0) and acetyl-L-carnitine (C2) were mainly detected in sperm. All other acylcarnitines were found in amounts less than 1 μmol. The four most abundant acylcarnitines in seminal plasma were C0, C2, butyryl-L-carnitine (C4) and propionyl-L-carnitine (C3). The detailed results are presented in Fig 4 and S2 Table.

### Correlations among acylcarnitines

The quantities of most acylcarnitines in sperm as well as in seminal plasma correlated with each other in a significant positive manner (*P* < 0.05). In contrast, significant cross-correlations between sperm and seminal plasma were observed only for C3 (*r*_*s*_ = 0.460) and hexadecadienyl-L-carnitine (C16:2, -0.453). The amounts of C3-DC-M/C5-OH and C4:1-DC/C6 in seminal plasma were significantly negatively correlated with those of nearly every other acylcarnitine species in sperm. The methylglutaryl-L-carnitine (C5-M-DC) content of sperm was significantly negatively correlated with the levels of C3, C6, octanoyl-L-carnitine (C8), C16:2 and all other C5 metabolites.

The C2 and C3 levels in sperm correlated positively with the levels of acylcarnitines in seminal plasma but were significantly negatively correlated with decenoyl-L-carnitine (C10:1) and dodecenoyl-L-carnitine (C12:1) levels. The correlation coefficients for the acylcarnitine concentrations in sperm with those in seminal plasma are presented in S8 Table.

### Correlations between acylcarnitine concentrations and spermiogram parameters

**Fig 5:**
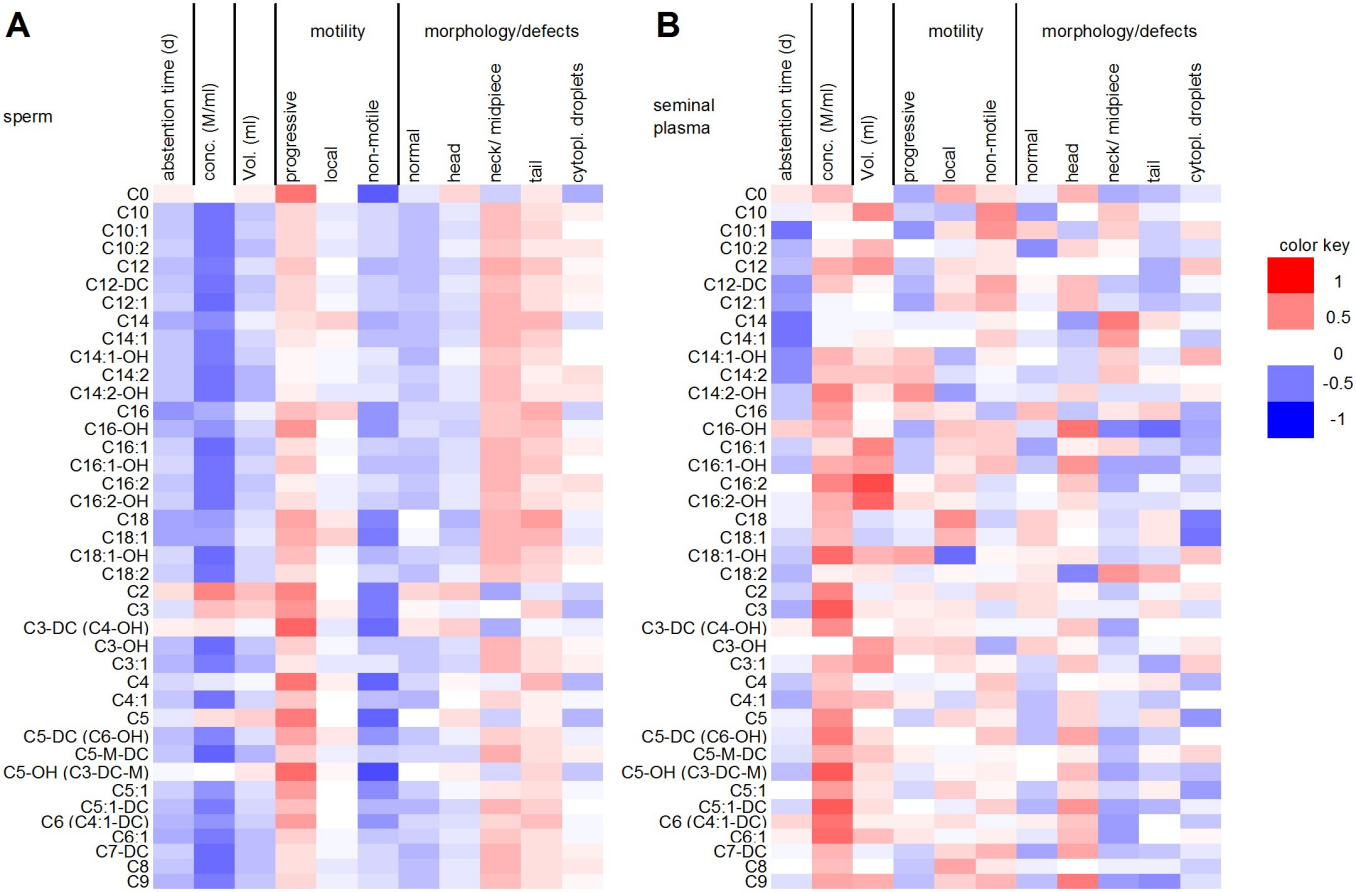
Heat map of the correlation coefficients between spermiogram parameters and the levels of acylcarnitines from ejaculated mature human sperm (A) and seminal plasma (B). Red colored cells represent positive correlations, and blue colored cells represent negative correlations.

The concentration of every acylcarnitine **in sperm** was significantly correlated (*P* < 0.05) with at least one spermiogram parameter (Fig 5A). In detail, the concentrations of most acylcarnitines, except C0, C16, C16-OH, C18, C18:1, C3, C3-DC, C4, C5, C5-OH, C5:1 and C6, were significantly negatively correlated with sperm concentration. Interestingly, those acylcarnitines that were not significantly correlated with sperm concentration were linked to sperm motility. Among them were the most abundant acylcarnitines, namely, C0, C2 and C3. This result is accordance with the relevance of acylcarnitines in the energy production of mammalian cells. The complete mechanisms were recently summarized in [48]. With respect to sperm, it has been shown that high concentrations of free acylcarnitines are positively correlated with progressive sperm movement [49]. Furthermore, a study reported lower C2 concentrations in semen of oligozoospermic men [50]. In accordance with this finding, Busetto and colleagues reported that L-carnitine, acetyl-L-carnitine, vitamins and zinc diet supplementation has a beneficial effect on sperm concentration and sperm motility in men with abnormal spermiograms that show oligo-, astheno-, or teratozoospermia [51].

In **seminal plasma**, in contrast to sperm, the acylcarnitine content tended to correlate positively with sperm concentration (Fig 5B). The correlations were significant for C14:2-OH, C16:2, C18:1-OH, C2, C3, C5, C5-DC, C5-OH, C5:1-DC, C6 and C6:1. This finding is consistent with an earlier comparison of infertile and fertile men that showed significantly reduced concentrations of free and total carnitine in the infertile cohort [52]. Because there were no significant associations of acylcarnitine levels with sperm motility, it might be reasonable to assume that acylcarnitines in seminal plasma are responsible for the protection of the sperm, particularly against oxidative damage.

The amount of C16-OH in seminal plasma was associated with morphological defects; specifically, it showed a significant positive association with head defects (*r*_*s*_ = 0.548) and a significant negative association with neck (-0.477) and tail defects (-0.563). A similar trend was observed for C9.

### Lipids

**Fig 6:**
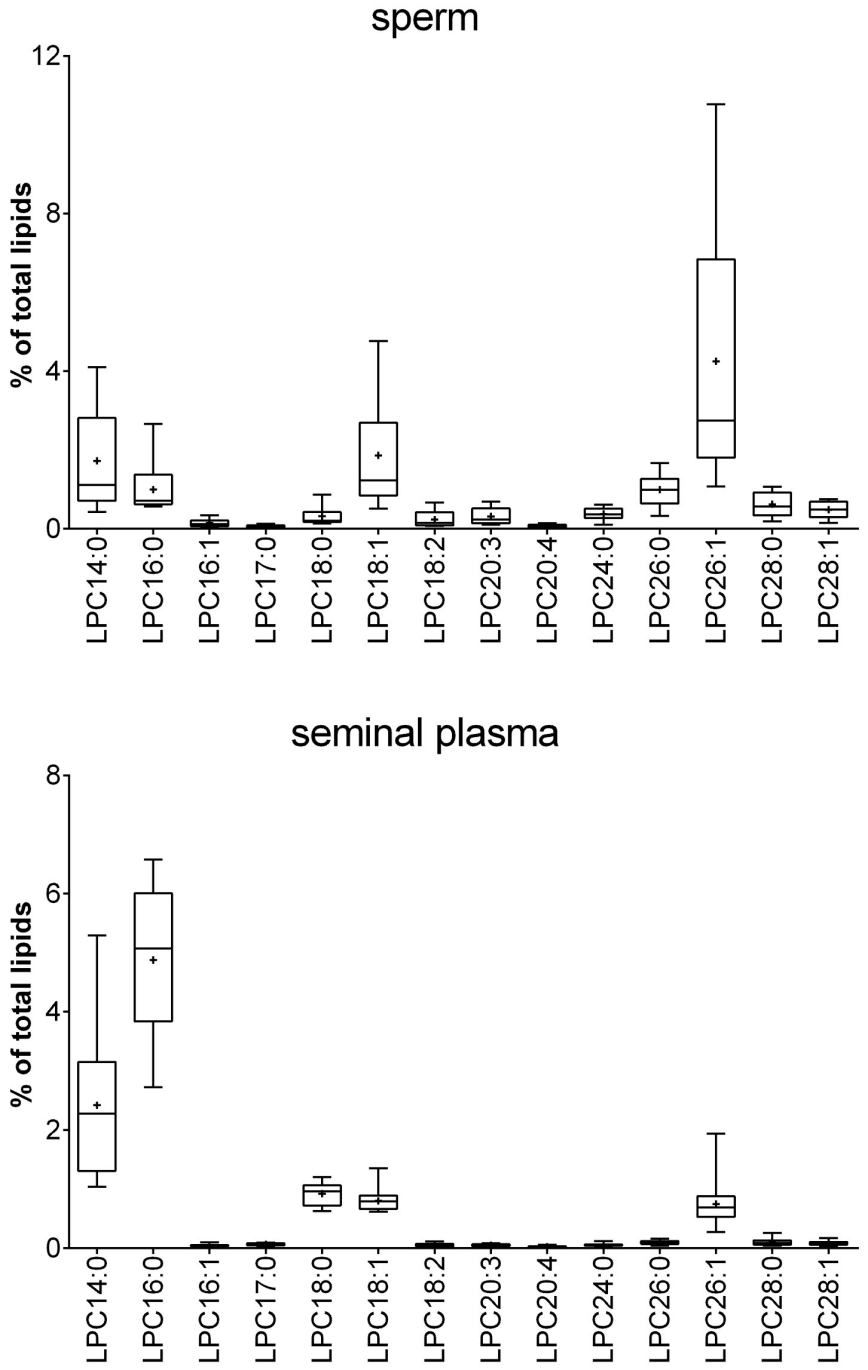
Relative lysophosphatidylcholine (LPC) concentrations in sperm and seminal plasma obtained from 20 healthy human donors. The box plots show the minimum, maximum and mean (black cross) values of the data. The relative amounts were calculated for each sample based on the total amount of lipids in the sample and are given in %. Total concentrations (in μmol) are given in S2 Table.

**Fig 7:**
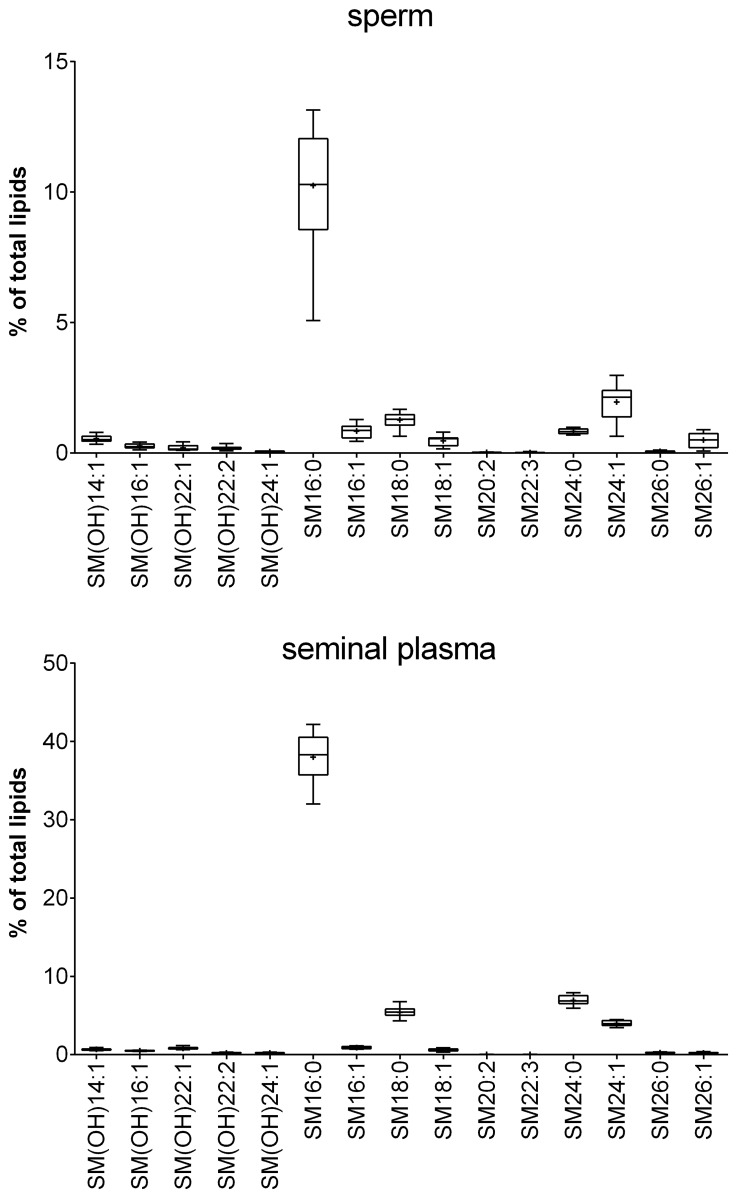
Relative sphingomyelin (SM) concentrations in sperm and seminal plasma obtained from 20 healthy human donors. The box plots show the minimum, maximum and mean (black cross) values of the data. The relative amounts were calculated for each sample based on the total amount of lipids in the sample and are given in %. Total concentrations (in μmol) are given in S2 Table.

**Fig 8:**
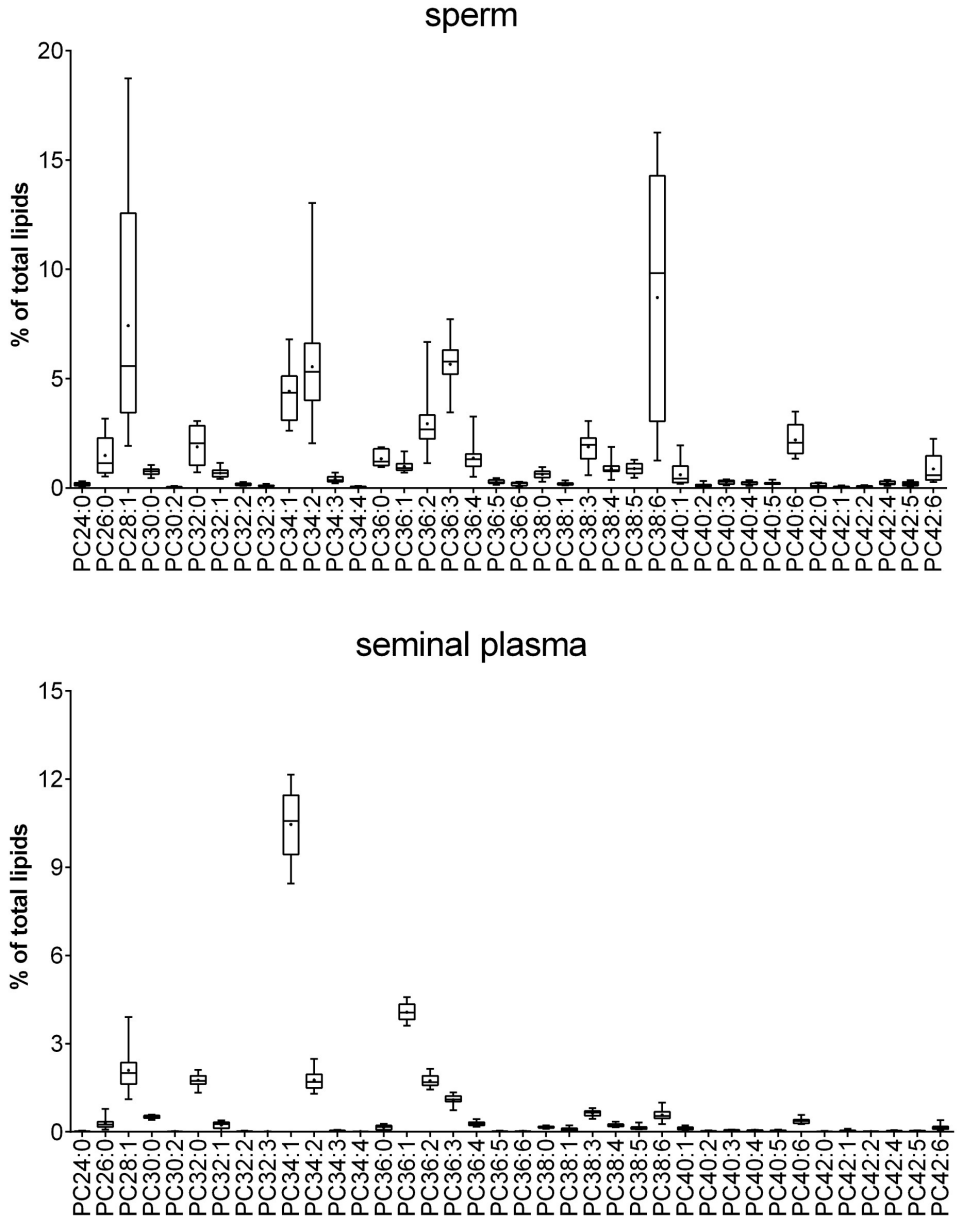
Relative phosphatidylcholine (PC) concentrations in sperm and seminal plasma obtained from 20 healthy human donors. The box plots show the minimum, maximum and mean (black cross) values of the data. The relative amounts were calculated for each sample based on the total amount of lipids in the sample and are given in %. Total concentrations (in μmol) are given in S2 Table.

**Fig 9:**
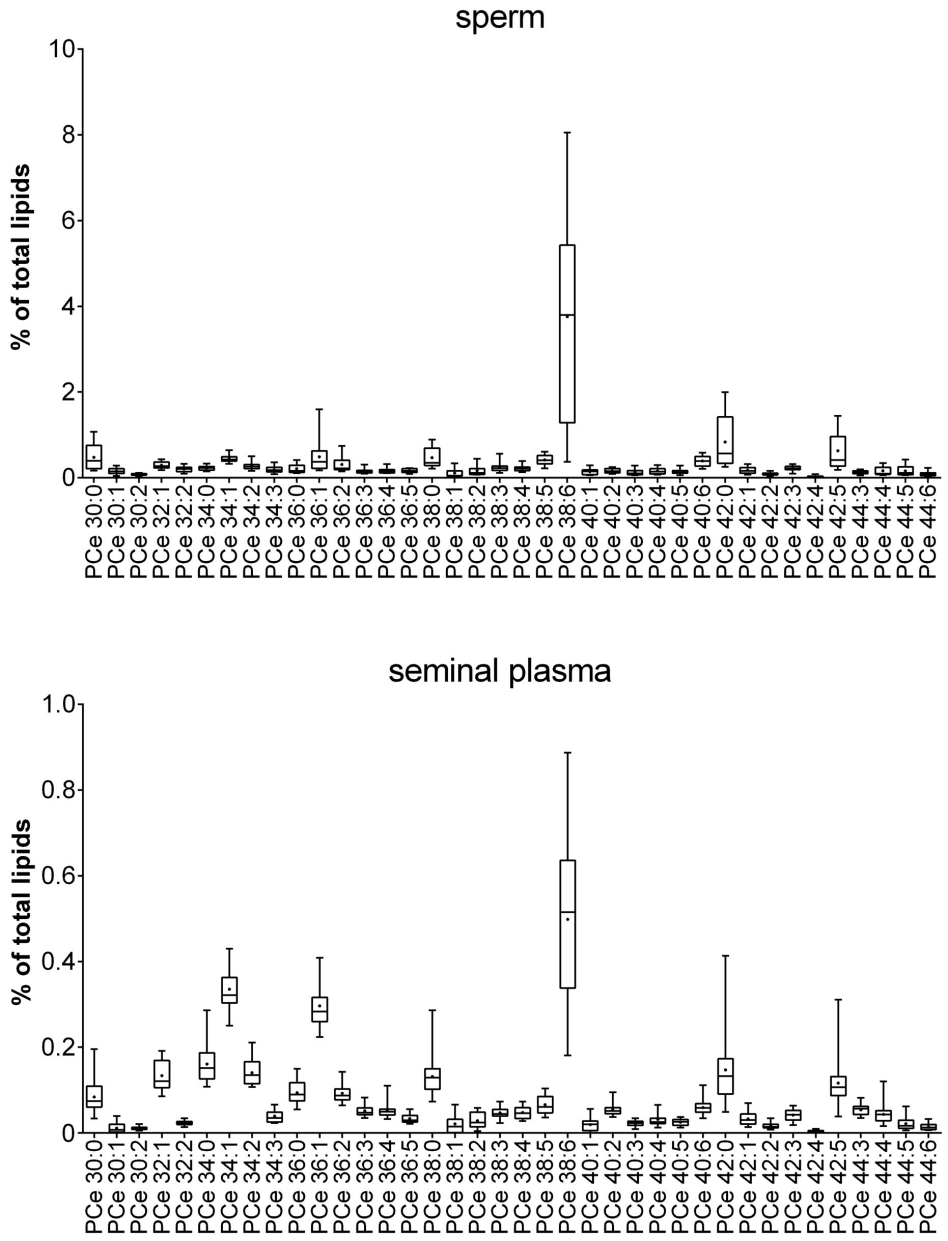
Relative etherphosphorylcholine (GPCe) concentrations in sperm and seminal plasma obtained from 20 healthy human donors. The box plots show the minimum, maximum and mean (black cross) values of the data. The relative amounts have been calculated for each sample based on the total amount of lipids in the sample and are given in %. Total concentrations (in μmol) are given in S2 Table.

Because most of the currently available metabolomic data describing sperm and seminal plasma have been either performed with NMR spectroscopy or GC-MS, there are no metabolomics data of the PC compositions and intact lipids available. The three most abundant lipids in sperm were sphingomyelin (SM) 16:0, PC 38:6 and PC 28:1. In seminal plasma, SM 16:0, PC 34:1 and SM 24:0 were present at the highest concentrations of all lipids investigated. These results are in accordance with previously published studies that relied on another MS-based technique, namely, MALDI-TOF MS [53,54]. For detailed results, please see Figs 6–9 and S2 Table.

### Correlations among lipids

**Fig 10:**
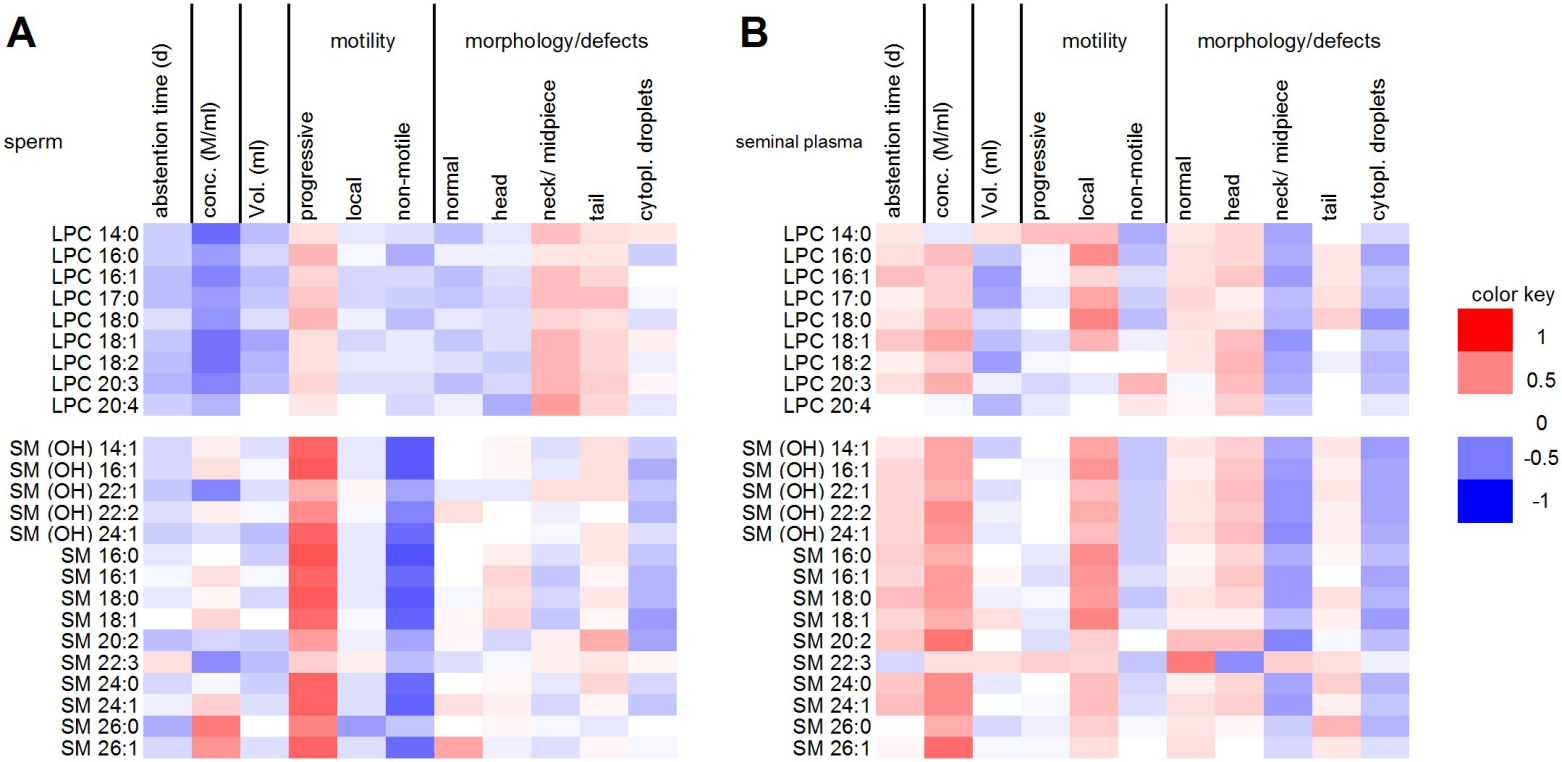
Heat map of the correlation coefficients between spermiogram parameters and the levels of lysophosphatidylcholines (LPC) and sphingomyelins (SM) from ejaculated mature human sperm (A) and seminal plasma (B). Red colored cells represent positive correlations, and blue colored cells represent negative correlations. Only metabolites that were present in ≥ 75% of the samples were considered in the correlation analyses.

**Fig 11:**
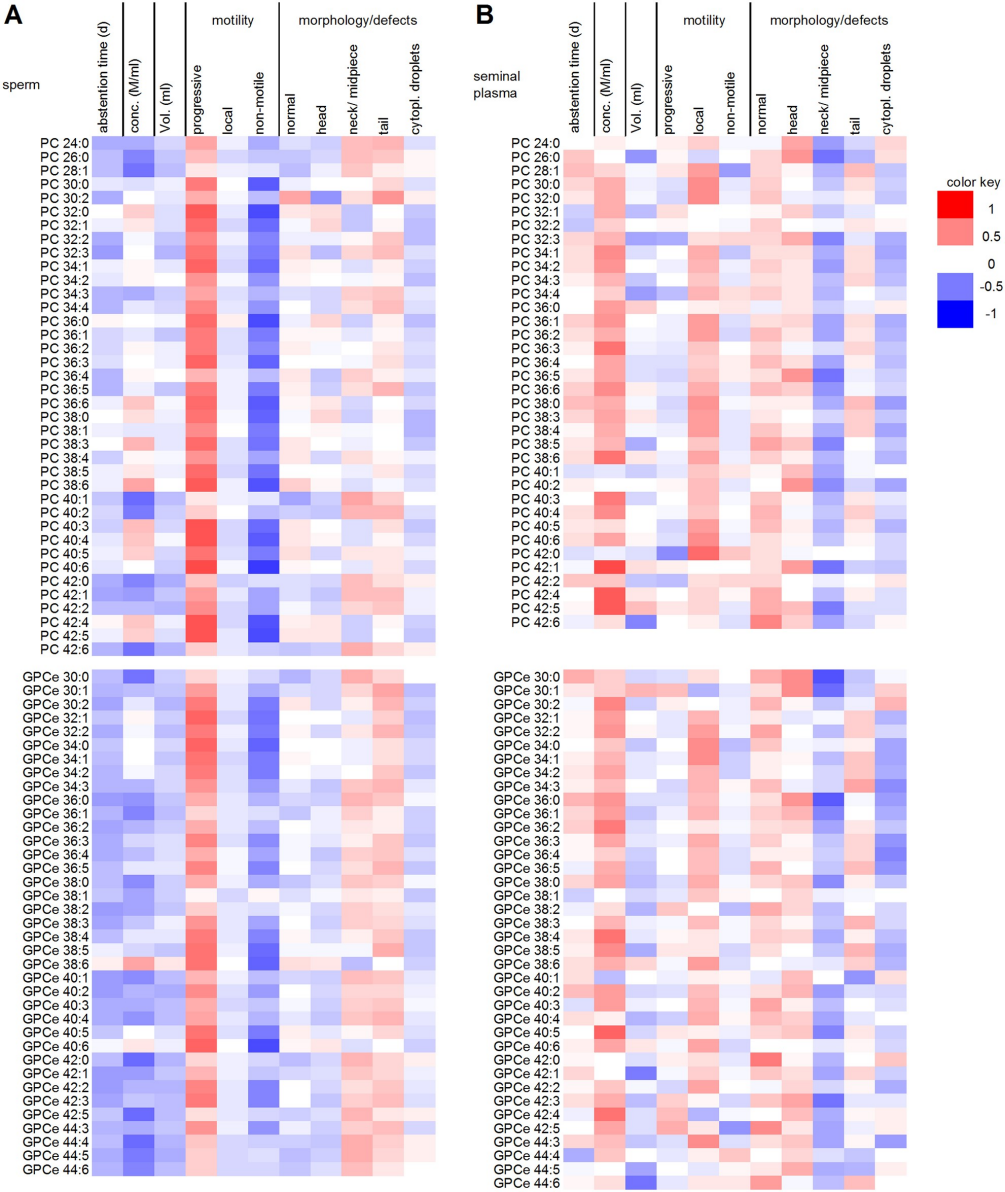
Heat map of the correlation coefficients between spermiogram parameters and the levels of phosphatidylcholines (PC) and ether-bound glycerophosphorylcholines (GPCe) from ejaculated mature human sperm (A) and seminal plasma (B). Red colored cells represent positive correlations, and blue colored cells represent negative correlations. Only metabolites that were present in ≥ 75% of the samples were included in the correlation analyses.

In sperm cells, the levels of all LPC species were correlated in a significantly positive manner (*P* < 0.05). This correlation was also observed for most PC, ether GPC (GPCe) and SM species (Figs 10A and 11A). Only a few correlations were found for PC 28:1, PC 30:2, PC 40:1, PC 42:6, GPCe 38:6 and SM 22:3 within the respective lipid classes.

The correlation analysis of LPC with PC species resulted in significant positive associations in most cases, except for LPC 14:0 and PC 38:6. Regarding the association of LPC with GPCe and SM in human spermatozoa, no associations were found for GPCe 38:6 and SM 26:0 levels with any LPC species.

With the exception of PC 38:6, most PC species also correlated with GPCe. Considering the associations between PC and SM as well as GPCe and SM species, only SM 22:3 did not correlate with most PC and GPCe species.

The results for seminal plasma were similar, and many significant correlations were found (Figs 10B and 11B). However, no associations were found for GPCe 40:1. Among the lipids investigated, fewer than ten correlations were found for LPC 14:0 (8), PC 26:0 (7), PC 36:0 (5), PC 40:1 (9), GPCe 42:0 (5), GPCe 42:5 (7), GPCe 44:5 and SM 22:3 (2). GPCe 44:4 was correlated only with GPCe 38:1, and GPCe 30:1 was correlated only with PC 40:2.

### Correlations between lipids and spermiogram parameters

**Fig 12:**
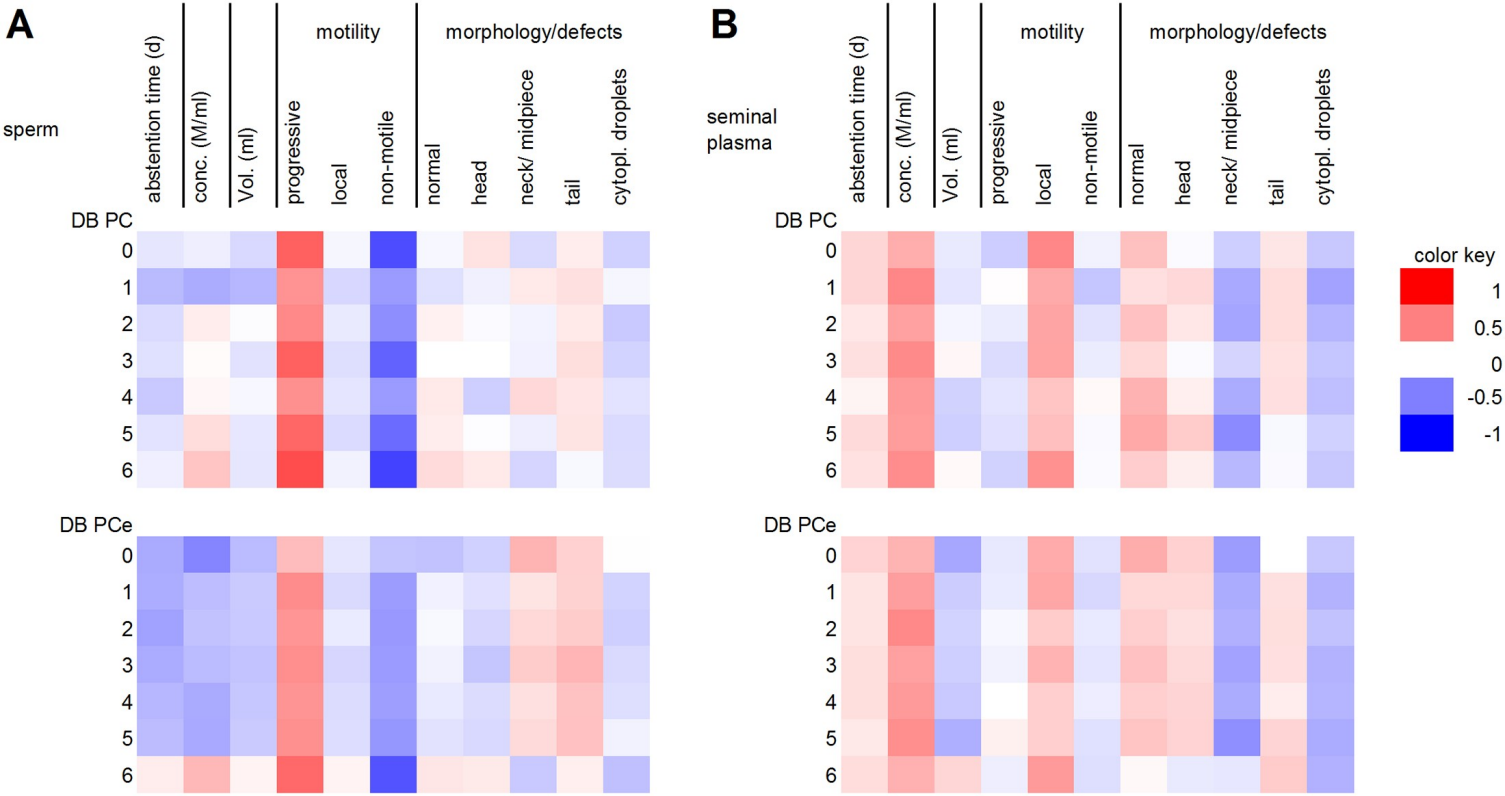
Heat map of the correlation coefficients between spermiogram parameters and the levels of phosphatidylcholines (PC) and ether-bound glycerophosphorylcholines (GPCe) with different double bond contents from ejaculated mature human sperm (A) and seminal plasma (B). Red colored cells represent positive correlations, and blue colored cells represent negative correlations.

Significant positive correlations with progressive motility were found for more than 50% (40 of 76) of the analyzed PC species and for 80% (12 of 15) of the analyzed SM species in sperm, indicating a decisive role of lipid metabolites in sperm motility. The classification of PC and PCe into subgroups with different double bond contents shows significant correlations of highly unsaturated PC and PCe in sperm cells with sperm motility (Fig 12). This is in accordance with publications showing, that the assembly of docosahexaenoic acid (C22:6) into sperm lipids during the maturation of sperm in the epididymis increases the fluidity of the membrane and, thus, promotes the motility of the sperm cells [10,55]. Overall, LPC and PC species tended to correlate negatively with sperm concentration, whereas no clear trend was observed for SM. Lysolipids (such as LPC) can act as detergents and are therefore capable of destroying lipid bilayers [56,57]. In this context, they also play a crucial role during the fertilization process [57,58]. As the sperm cell approaches the oocyte, the concentration of lysolipids in the sperm cell membrane increases, and lysolipids facilitate the fusion of the sperm and oocyte membranes [59]. However, if the lysolipid concentration in the sperm membrane increases too early, the cell can be destroyed before it reaches its final destination. Increased LPC concentrations have been shown in the sperm of very obese patients [60]. Interestingly, no correlations between lipid concentrations and morphological features were found in the present study; such associations would have been expected if the structure of the sperm membrane was damaged.

In contrast to that of sperm, the lipid concentration of seminal plasma was positively associated with sperm concentration and local motility and tended to correlate negatively with morphological abnormalities involving neck/midpiece defects. A study on Chinese patients reported that the levels of triglycerides, total cholesterol, and low- and high-density lipoproteins in seminal plasma were higher in individuals with oligo-, astheno- or teratozoospermia than in individuals with normal sperm concentration, motility and morphology [61]. These lipid species were not investigated in the present study. A significant positive correlation with normomorphic sperm was found for the levels of PC 42:6, GPCe 42:0, GPCe 42:5 and SM 22:3 in seminal plasma.

A complete list of the observed correlations between the sperm and seminal plasma components and the spermiogram parameters is provided in S6 and S7 Tables. The correlation coefficients for lipid concentrations in sperm with those in seminal plasma are shown in S9–S12 Tables. The results of the correlation analysis indicate that only a small exchange of metabolites occurs between sperm and seminal plasma.

## Conclusion

Here, we present the first targeted approach to a metabolomic analysis of human sperm and seminal plasma using sophisticated and reliable sample preparation methods and liquid chromatography/flow injection analysis coupled with electrospray ionization mass spectrometry under high-throughput conditions. This pilot study is a first step in linking traditional spermiogram parameters with sophisticated mass spectrometric tools. Although further studies will be necessary to define the normal range in the concentrations of the metabolites in the sperm and seminal plasma of healthy men, the multiple correlations with standard sperm parameters found in this study demonstrate that metabolomics is a very useful tool in the identification of biomarkers for male infertility. As a next step, metabolomic investigations of semen samples from subfertile and infertile men should be performed.

## Supporting information

S1 Fig Summary of the study design and outcome. Each ejaculate was analyzed manually and information about the abstention time, semen volume, pH, leucocyte concentration, sperm concentration, sperm motility and sperm morphology were recorded. Subsequently, the semen sample was separated by density gradient centrifugation and the mature sperm fraction and the seminal plasma were collected. Both were analyzed by a targeted metabolomics approach. Whereas in sperm 171 out of 180 metabolites were identified, 177 out of 180 metabolites were identified in seminal plasma. Concentrations of sperm metabolites were adjusted to 100 × 10^6^ cells. Metabolite concentrations were correlated to spermiogram parameters. Overall, the metabolome of sperm is more closely related to sperm count and sperm motility, whereas the metabolome of the seminal plasma is more closely related to sperm count and sperm morphology.
(TIF)

S1 Table Age, body mass index (BMI) and spermiogram parameters of 20 healthy human subjects. Data illustrate the quantitative aspects of the specimens as well as their characteristics with respect to sperm motility and morphology (left column). Data of donors are given as mean ± SD. For comparison the reference ranges established by the WHO (5^th^ edition, 2010) are shown in the right column.
(DOCX)

S2 Table Concentrations of metabolites in sperm and seminal plasma of 20 healthy human subjects. Data are given in μmol as mean ± SD. Abbreviations: Ala—alanine, Arg—arginine, Asn—asparagine, Asp—aspartate, Cit—citrulline, Gln—glutamine, Glu—glutamate, Gly—glycine, His—histidine, Ile—isoleucine, Leu—leucine, Lys—lysine, Met—methionine, Orn—ornithine, Phe—phenylalanine, Pro—proline, Ser—serine, Thr—threonine, Trp—tryptophan, Tyr—tyrosine, Val—valine, Ac-Orn—acetylornithine, ADMA—asymmetrically dimethylated arginine, alpha-AAA—alpha-aminoadipic acid, Met-SO—methionine sulfoxide, Nitro-Tyr—nitrotyrosine, OH-Pro—hydroxyproline, PEA—phenylethylamine, DMA—dimethylamine, C0—DL-carnitine, C10—decanoyl-L-carnitine, C10:1—decenoyl-L-carnitine, C10:2—decadienyl-L-carnitine, C12—dodecanoyl-L-carnitine, C12:1—dodecenoyl-L-carnitine, C12-DC—dodecanedioyl-L-carnitine, C14—tetradecanoyl-L-carnitine, C14:1—tetradecenoyl-L-carnitine, C14:1-OH—hydroxytetradecenoyl-L-carnitine, C14:2—tetradecadienyl-L-carnitine, C14:2-OH—hydroxytetradecadienyl-L-carnitine, C16—hexadecanoyl-L-carnitine, C16:1—hexadecenoyl-L-carnitine, C16:1-OH—hydroxyhexadecenoyl-L-carnitine, C16:2—hexadecadienyl-L-carnitine, C16:2-OH—hydroxyhexadecadienyl-L-carnitine, C16-OH—hydroxyhexadecanoyl-L-carnitine, C18—octadecanoyl-L-carnitine, C18:1—octadecenoyl-L-carnitine, C18:1-OH—hydroxyoctadecenoyl-L-carnitine, C18:2—octadecadienyl-L-carnitine, C2—acetyl-L-carnitine, C3—propionyl-L-carnitine, C3:1—propenyl-L-carnitine, C3-DC/C4-OH—malonyl-L-carnitine/hydroxybutyryl-L-carnitine, C3-DC-M/C5-OH—methylmalonyl-L-carnitine/hydroxyvaleryl-L-carnitine, C3-OH—hydroxypropionyl-L-carnitine, C4—butyryl-L-carnitine, C4:1—butenyl-L-carnitine, C4:1-DC/C6—fumaryl-L-carnitine/hexanoyl-L-carnitine, C5—valeryl-L-carnitine, C5:1—tiglyl-L-carnitine, C5:1-DC—glutaconyl-L-carnitine, C5-DC/C6-OH—glutaryl-L-carnitine/hydroxyhexanoyl-L-carnitine, C5-M-DC—methylglutaryl-L-carnitine, C6:1—hexenoyl-L-carnitine, C7-DC—pimelyl-L-carnitine, C8—octanoyl-L-carnitine, C8:1—octenoyl-L-carnitine, C9—nonayl-L-carnitine, LPC—lysophosphatidylcholine, PC—phosphatidylcholine, GPCe—etherphosphorylcholine, SM—sphingomyelin.
(DOCX)

S3 Table Correlation analysis of amino acids in sperm of 20 healthy donors. Data are Spearman correlation rank coefficients. Significant correlations are highlighted in bolt red. Abbreviations: Ala—alanine, Arg—arginine, Asn—asparagine, Gln—glutamine, Glu—glutamate, Ile—isoleucine, Leu—leucine, Phe—phenylalanine, Pro—proline, Ser—serine, Thr—threonine, Tyr—tyrosine.
(DOCX)

S4 Table Correlation analysis of amino acids in seminal plasma of 20 healthy donors. Data are Spearman correlation rank coefficients. Significant correlations are highlighted in bolt. Abbreviations: Ala—alanine, Arg—arginine, Asn—asparagine, Asp—aspartate, Cit—citrulline, Gln—glutamine, Glu—glutamate, Gly—glycine, His—histidine, Ile—isoleucine, Leu—leucine, Lys—lysine, Met—methionine, Orn—ornithine, Phe—phenylalanine, Pro—proline, Ser—serine, Thr—threonine, Trp—tryptophan, Tyr—tyrosine, Val—valine.
(DOCX)

S5 Table Correlation analysis of amino acids in sperm and seminal plasma (SP) of 20 healthy donors. Data are Spearman correlation rank coefficients. Significant correlations are highlighted in bolt. Abbreviations: Ala—alanine, Arg—arginine, Asn—asparagine, Asp—aspartate, Cit—citrulline, Gln—glutamine, Glu—glutamate, Gly—glycine, His—histidine, Ile—isoleucine, Leu—leucine, Lys—lysine, Met—methionine, Orn—ornithine, Phe—phenylalanine, Pro—proline, Ser—serine, Thr—threonine, Trp—tryptophan, Tyr—tyrosine, Val—valine.
(DOCX)

S6 Table Correlation analysis of metabolites in sperm of 20 healthy donors with spermiogram parameters. Data are Spearman correlation rank coefficients. Significant positive correlations are highlighted in red, significant negative correlations in blue. Abbreviations: Ala—alanine, Arg—arginine, Asn—asparagine, Gln—glutamine, Glu—glutamate, Gly—glycine, His—histidine, Ile—isoleucine, Leu—leucine, Phe—phenylalanine, Pro—proline, Ser—serine, Thr—threonine, Tyr—tyrosine, ADMA—asymmetrically dimethylated arginine, DMA—dimethylamine, C0—DL-carnitine, C10—decanoyl-L-carnitine, C10:1—decenoyl-L-carnitine, C10:2—decadienyl-L-carnitine, C12—dodecanoyl-L-carnitine, C12:1—dodecenoyl-L-carnitine, C12-DC—dodecanedioyl-L-carnitine, C14—tetradecanoyl-L-carnitine, C14:1—tetradecenoyl-L-carnitine, C14:1-OH—hydroxytetradecenoyl-L-carnitine, C14:2—tetradecadienyl-L-carnitine, C14:2-OH—hydroxytetradecadienyl-L-carnitine, C16—hexadecanoyl-L-carnitine, C16:1—hexadecenoyl-L-carnitine, C16:1-OH—hydroxyhexadecenoyl-L-carnitine, C16:2—hexadecadienyl-L-carnitine, C16:2-OH—hydroxyhexadecadienyl-L-carnitine, C16-OH—hydroxyhexadecanoyl-L-carnitine, C18—octadecanoyl-L-carnitine, C18:1—octadecenoyl-L-carnitine, C18:1-OH—hydroxyoctadecenoyl-L-carnitine, C18:2—octadecadienyl-L-carnitine, C2—acetyl-L-carnitine, C3—propionyl-L-carnitine, C3:1—propenyl-L-carnitine, C3-DC/C4-OH—malonyl-L-carnitine/hydroxybutyryl-L-carnitine, C3-DC-M/C5-OH—methylmalonyl-L-carnitine/hydroxyvaleryl-L-carnitine, C3-OH—hydroxypropionyl-L-carnitine, C4—butyryl-L-carnitine, C4:1—butenyl-L-carnitine, C4:1-DC/C6—fumaryl-L-carnitine/hexanoyl-L-carnitine, C5—valeryl-L-carnitine, C5:1—tiglyl-L-carnitine, C5:1-DC—glutaconyl-L-carnitine, C5-DC/C6-OH—glutaryl-L-carnitine/hydroxyhexanoyl-L-carnitine, C5-M-DC—methylglutaryl-L-carnitine, C6:1—hexenoyl-L-carnitine, C7-DC—pimelyl-L-carnitine, C8—octanoyl-L-carnitine, C8:1—octenoyl-L-carnitine, C9—nonayl-L-carnitine, LPC—lysophosphatidylcholine, PC—phosphatidylcholine, GPCe—etherphosphorylcholine, SM—sphingomyelin.
(DOCX)

S7 Table Correlation analysis of metabolites in seminal plasma with spermiogram parameters of 20 healthy donors. Data are Spearman correlation rank coefficients. Significant positive correlations are highlighted in red, significant negative correlations in blue. Abbreviations: Ala—alanine, Arg—arginine, Asn—asparagine, Asp—aspartate, Cit—citrulline, Gln—glutamine, Glu—glutamate, Gly—glycine, His—histidine, Ile—isoleucine, Leu—leucine, Lys—lysine, Met—methionine, Orn—ornithine, Phe—phenylalanine, Pro—proline, Ser—serine, Thr—threonine, Trp—tryptophan, Tyr—tyrosine, Val—valine, ADMA—asymmetrically dimethylated arginine, alpha-AAA—α-aminoadipic acid, OH-Pro—hydroxyproline, DMA—dimethylamine, C0—DL-carnitine, C10—decanoyl-L-carnitine, C10:1—decenoyl-L-carnitine, C10:2—decadienyl-L-carnitine, C12—dodecanoyl-L-carnitine, C12:1—dodecenoyl-L-carnitine, C12-DC—dodecanedioyl-L-carnitine, C14—tetradecanoyl-L-carnitine, C14:1—tetradecenoyl-L-carnitine, C14:1-OH—hydroxytetradecenoyl-L-carnitine, C14:2—tetradecadienyl-L-carnitine, C14:2-OH—hydroxytetradecadienyl-L-carnitine, C16—hexadecanoyl-L-carnitine, C16:1—hexadecenoyl-L-carnitine, C16:1-OH—hydroxyhexadecenoyl-L-carnitine, C16:2—hexadecadienyl-L-carnitine, C16:2-OH—hydroxyhexadecadienyl-L-carnitine, C16-OH—hydroxyhexadecanoyl-L-carnitine, C18—octadecanoyl-L-carnitine, C18:1—octadecenoyl-L-carnitine, C18:1-OH—hydroxyoctadecenoyl-L-carnitine, C18:2—octadecadienyl-L-carnitine, C2—acetyl-L-carnitine, C3—propionyl-L-carnitine, C3:1—propenyl-L-carnitine, C3-DC/C4-OH—malonyl-L-carnitine/hydroxybutyryl-L-carnitine, C3-DC-M/C5-OH—methylmalonyl-L-carnitine/hydroxyvaleryl-L-carnitine, C3-OH—hydroxypropionyl-L-carnitine, C4—butyryl-L-carnitine, C4:1—butenyl-L-carnitine, C4:1-DC/C6—fumaryl-L-carnitine/hexanoyl-L-carnitine, C5—valeryl-L-carnitine, C5:1—tiglyl-L-carnitine, C5:1-DC—glutaconyl-L-carnitine, C5-DC/C6-OH—glutaryl-L-carnitine/hydroxyhexanoyl-L-carnitine, C5-M-DC—methylglutaryl-L-carnitine, C6:1—hexenoyl-L-carnitine, C7-DC—pimelyl-L-carnitine, C8—octanoyl-L-carnitine, C8:1—octenoyl-L-carnitine, C9—nonayl-L-carnitine, LPC—lysophosphatidylcholine, PC—phosphatidylcholine, GPCe—etherphosphorylcholine, SM—sphingomyelin.
(DOCX)

S8 Table Correlation analysis of acylcarnitines in sperm with those in seminal plasma (SP) of 20 healthy donors. Data are Spearman correlation rank coefficients. Significances are highlighted in bolt. Abbreviations: C0—DL-carnitine, C10—decanoyl-L-carnitine, C10:1—decenoyl-L-carnitine, C10:2—decadienyl-L-carnitine, C12—dodecanoyl-L-carnitine, C12:1—dodecenoyl-L-carnitine, C12-DC—dodecanedioyl-L-carnitine, C14—tetradecanoyl-L-carnitine, C14:1—tetradecenoyl-L-carnitine, C14:1-OH—hydroxytetradecenoyl-L-carnitine, C14:2—tetradecadienyl-L-carnitine, C14:2-OH—hydroxytetradecadienyl-L-carnitine, C16—hexadecanoyl-L-carnitine, C16:1—hexadecenoyl-L-carnitine, C16:1-OH—hydroxyhexadecenoyl-L-carnitine, C16:2—hexadecadienyl-L-carnitine, C16:2-OH—hydroxyhexadecadienyl-L-carnitine, C16-OH—hydroxyhexadecanoyl-L-carnitine, C18—octadecanoyl-L-carnitine, C18:1—octadecenoyl-L-carnitine, C18:1-OH—hydroxyoctadecenoyl-L-carnitine, C18:2—octadecadienyl-L-carnitine, C2—acetyl-L-carnitine, C3—propionyl-L-carnitine, C3:1—propenyl-L-carnitine, C3-DC/C4-OH—malonyl-L-carnitine/hydroxybutyryl-L-carnitine, C3-DC-M/C5-OH—methylmalonyl-L-carnitine/hydroxyvaleryl-L-carnitine, C3-OH—hydroxypropionyl-L-carnitine, C4—butyryl-L-carnitine, C4:1—butenyl-L-carnitine, C4:1-DC/C6—fumaryl-L-carnitine/hexanoyl-L-carnitine, C5—valeryl-L-carnitine, C5:1—tiglyl-L-carnitine, C5:1-DC—glutaconyl-L-carnitine, C5-DC/C6-OH—glutaryl-L-carnitine/hydroxyhexanoyl-L-carnitine, C5-M-DC—methylglutaryl-L-carnitine, C6:1—hexenoyl-L-carnitine, C7-DC—pimelyl-L-carnitine, C8—octanoyl-L-carnitine, C8:1—octenoyl-L-carnitine, C9—nonayl-L-carnitine.
(DOCX)

S9 Table Correlation analysis of lysophospholipids in sperm with lyso-phosphatidylcholines (LPC), phosphatidylcholines (PC), etherphosphorylcholines (GPCe) and sphingomyelins (SM) in seminal plasma (SP) of 20 healthy donors. Data are Spearman correlation rank coefficients. Significances are highlighted in bolt.
(DOCX)

S10 Table Correlation analysis of phosphatidylcholines in sperm with lyso-phosphatidylcholines (LPC), phosphatidylcholines (PC), etherphosphorylcholines (GPCe) and sphingomyelins (SM) in seminal plasma (SP) of 20 healthy donors. Data are Spearman correlation rank coefficients. Significances are highlighted in bolt.
(DOCX)

S11 Table Correlation analysis of ether-phosphatidylcholines in sperm with lyso-phosphatidylcholines (LPC), phosphatidylcholines (PC), etherphosphorylcholines (GPCe) and sphingomyelins (SM) in seminal plasma (SP) of 20 healthy donors. Data are Spearman correlation rank coefficients. Significances are highlighted in bolt.
(DOCX)

S12 Table Correlation analysis of sphingomyelins (SM) in sperm with lyso-phosphatidylcholines (LPC), phosphatidylcholines (PC), etherphosphorylcholines (GPCe) and SM in seminal plasma (SP) of 20 healthy donors. Data are Spearman correlation rank coefficients. Significances are highlighted in bolt.
(DOCX)

## Abbreviations

Ac-Orn: acetylornithine
ADMA: asymmetrically dimethylated arginine
alpha-AAA: α-aminoadipic acid
Arg: arginine
Asp: aspartate
BMI: body mass index
C0: DL-carnitine
C10:1: decenoyl-L-carnitine
C12:1: dodecenoyl-L-carnitine
C14:2-OH: hydroxytetradecadienyl-L-carnitine
C16: hexadecanoyl-L-carnitine
C16:2: hexadecadienyl-L-carnitine
C16-OH: hydroxyhexadecanoyl-L-carnitine
C18: octadecanoyl-L-carnitine
C18:1: octadecenoyl-L-carnitine
C18:1-OH: hydroxyoctadecenoyl-L-carnitine
C2: acetyl-L-carnitine
C3: propionyl-L-carnitine
C4: butyryl-L-carnitine
C4-OH (C3-DC): hydroxybutyryl-L-carnitine
C5: valeryl-L-carnitine
C5:1: tiglyl-L-carnitine
C5:1-DC: glutaconyl-L-carnitine
C5-DC (C6-OH): glutaryl-L-carnitine (hydroxyhexanoyl-L-carnitine)
C5-M-DC: methylglutaryl-L-carnitine
C5-OH (C3-DC-M): hydroxyvaleryl-L-carnitine (methylmalonyl-L-carnitine)
C6 (C4:1-DC): hexanoyl-L-carnitine (fumaryl-L-carnitine)
C6:1: hexenoyl-L-carnitine
C8: octanyl-L-carnitine
C9: nonaylcarnitine
CASA: computer-assisted sperm analysis
Cit: Citrulline
ESI: electrospray ionization
FIA: flow injection analysis
GC: gas chromatography
Gln: glutamine
Gly: glycine
GPCe: etherphosphorylcholine
His: histidine
ICSI: intacytoplasmic sperm injection
Ile: isoleucine
IVF: *in vitro* fertilization
LC: liquid chromatography
Leu: leucine
LPC: lysophosphatidylcholine
Lys: lysine
Met: methionine
Met-SO: methionine sulfoxide
MS: mass spectrometry
Nitro-Tyr: 3-nitrotyrosine
NMR: nuclear magnetic resonance
OH-Pro: L-4-hydroxyproline
Orn: ornithine
PC: phosphatidylcholine
PEA: phenethylamine
Phe: phenylalanine
PITC: phenylisothiocyanate
*r_s_*: Spearman correlation coefficient
SD: standard deviation
SM: sphingomyelin
total DMA: total dimethylamine
Trp: tryptophan
Tyr: tyrosine
UPLC: ultra-performance liquid chromatography
Val: valine
WHO: World Health Organization
