# Supplementary figures and images for: Metabolomic profiling reveals correlations between spermiogram parameters and the metabolites present in human spermatozoa and seminal plasma
Source: PLoS One. 2019 Feb 20;14(2):e0211679. doi: 10.1371/journal.pone.0211679 (PMC6382115; doi:10.1371/journal.pone.0211679)

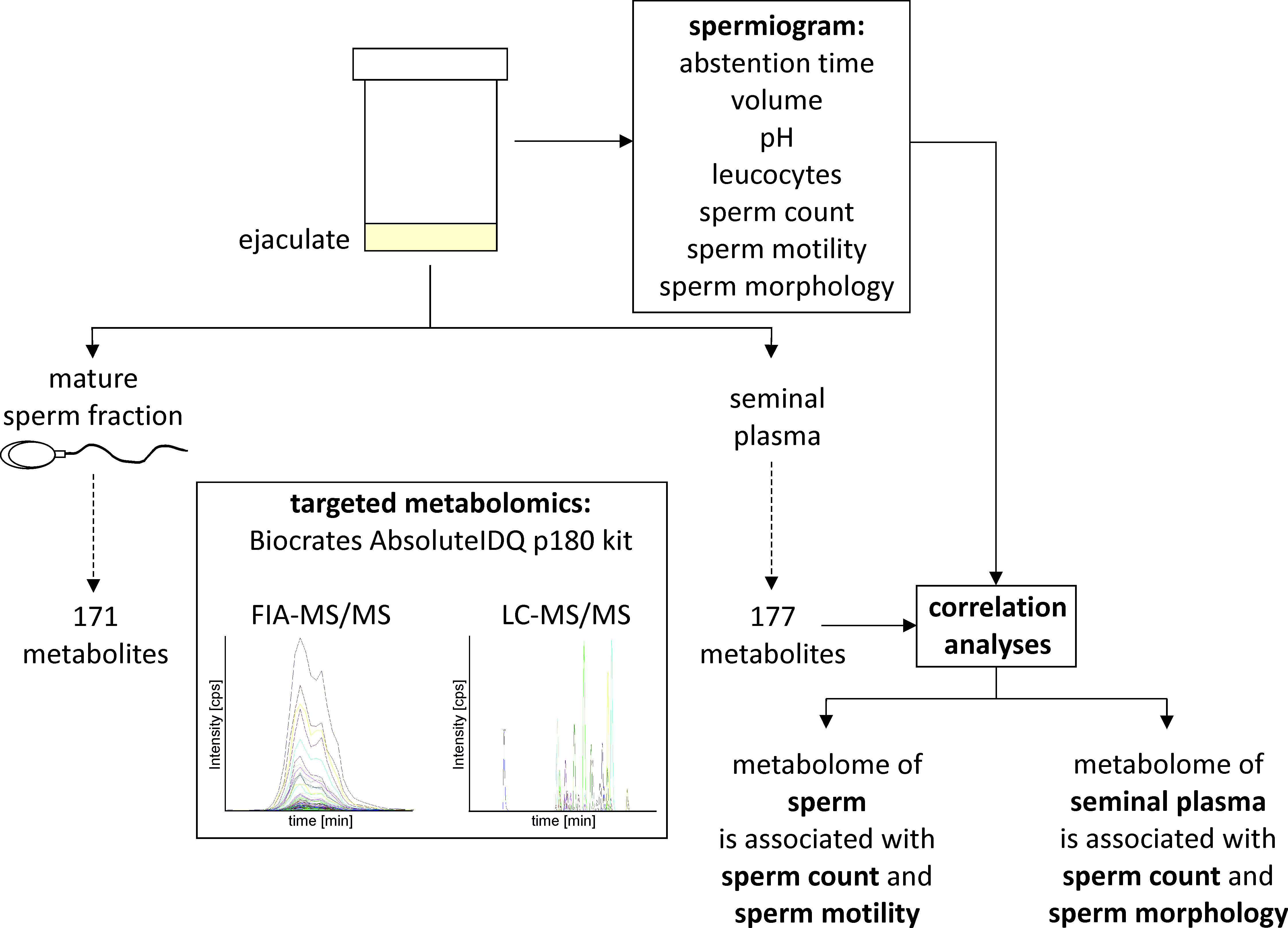

Supplement: S1 Fig — Each ejaculate was analyzed manually and information about the abstention time, semen volume, pH, leucocyte concentration, sperm concentration, sperm motility and sperm morphology were recorded. Subsequently, the semen sample was separated by density gradient centrifugation and the mature sperm fraction and the seminal plasma were collected. Both were analyzed by a targeted metabolomics approach. Whereas in sperm 171 out of 180 metabolites were identified, 177 out of 180 metabolites were identified in seminal plasma. Concentrations of sperm metabolites were adjusted to 100 × 106 cells. Metabolite concentrations were correlated to spermiogram parameters. Overall, the metabolome of sperm is more closely related to sperm count and sperm motility, whereas the metabolome of the seminal plasma is more closely related to sperm count and sperm morphology. (TIF) [file pone.0211679.s001.tif]
