# Supplementary material for: Metabolomic profiling reveals correlations between spermiogram parameters and the metabolites present in human spermatozoa and seminal plasma
Source: PLoS One. 2019 Feb 20;14(2):e0211679. doi: 10.1371/journal.pone.0211679 (PMC6382115; doi:10.1371/journal.pone.0211679)
Supplement: S1 Table — Data illustrate the quantitative aspects of the specimens as well as their characteristics with respect to sperm motility and morphology (left column). Data of donors are given as mean ± SD. For comparison the reference ranges established by the WHO (5th edition, 2010) are shown in the right column. (DOCX) [file pone.0211679.s002.docx]

|  | donors | WHO 5^th^ ed. |
| --- | --- | --- |
| age (y) | 31.7 ± 6.8 | - |
| BMI | 22.4 ± 2.2 | - |
| abstention (d) | 3.6 ± 1.1 | 2 - 7 |
| concentration (M/ml) | 145 ± 107 | ≥ 15 |
| volume (ml) | 3.2 ± 1.1 | - |
|  |  |  |
| motility (%) |  |  |
| progressive | 47.5 ± 12.8 | ≥ 40 |
| local | 9.9 ± 5.7 |  |
| non-motile | 40.3 ± 7.3 | - |
|  |  |  |
| morphology/defects (%) |  |  |
| normomorphic | 2.3 ± 1.9 | ≥ 4 |
| head | 76.5 ± 7.3 | - |
| neck/midpiece | 14.7 ± 3.7 | - |
| tail | 5.2 ± 3.2 | - |
| cytoplasmic | 0.7 ± 1.0 | - |
