# Supplementary material for: Metabolomic profiling reveals correlations between spermiogram parameters and the metabolites present in human spermatozoa and seminal plasma
Source: PLoS One. 2019 Feb 20;14(2):e0211679. doi: 10.1371/journal.pone.0211679 (PMC6382115; doi:10.1371/journal.pone.0211679)
Supplement: S2 Table — Data are given in μmol as mean ± SD. Abbreviations: Ala—alanine, Arg—arginine, Asn—asparagine, Asp—aspartate, Cit—citrulline, Gln—glutamine, Glu—glutamate, Gly—glycine, His—histidine, Ile—isoleucine, Leu—leucine, Lys—lysine, Met—methionine, Orn—ornithine, Phe—phenylalanine, Pro—proline, Ser—serine, Thr—threonine, Trp—tryptophan, Tyr—tyrosine, Val—valine, Ac-Orn—acetylornithine, ADMA—asymmetrically dimethylated arginine, alpha-AAA—alpha-aminoadipic acid, Met-SO—methionine sulfoxide, Nitro-Tyr—nitrotyrosine, OH-Pro—hydroxyproline, PEA—phenylethylamine, DMA—dimethylamine, C0—DL-carnitine, C10—decanoyl-L-carnitine, C10:1—decenoyl-L-carnitine, C10:2—decadienyl-L-carnitine, C12—dodecanoyl-L-carnitine, C12:1—dodecenoyl-L-carnitine, C12-DC—dodecanedioyl-L-carnitine, C14—tetradecanoyl-L-carnitine, C14:1—tetradecenoyl-L-carnitine, C14:1-OH—hydroxytetradecenoyl-L-carnitine, C14:2—tetradecadienyl-L-carnitine, C14:2-OH—hydroxytetradecadienyl-L-carnitine, C16—hexadecanoyl-L-carnitine, C16:1—hexadecenoyl-L-carnitine, C16:1-OH—hydroxyhexadecenoyl-L-carnitine, C16:2—hexadecadienyl-L-carnitine, C16:2-OH—hydroxyhexadecadienyl-L-carnitine, C16-OH—hydroxyhexadecanoyl-L-carnitine, C18—octadecanoyl-L-carnitine, C18:1—octadecenoyl-L-carnitine, C18:1-OH—hydroxyoctadecenoyl-L-carnitine, C18:2—octadecadienyl-L-carnitine, C2—acetyl-L-carnitine, C3—propionyl-L-carnitine, C3:1—propenyl-L-carnitine, C3-DC/C4-OH—malonyl-L-carnitine/hydroxybutyryl-L-carnitine, C3-DC-M/C5-OH—methylmalonyl-L-carnitine/hydroxyvaleryl-L-carnitine, C3-OH—hydroxypropionyl-L-carnitine, C4—butyryl-L-carnitine, C4:1—butenyl-L-carnitine, C4:1-DC/C6—fumaryl-L-carnitine/hexanoyl-L-carnitine, C5—valeryl-L-carnitine, C5:1—tiglyl-L-carnitine, C5:1-DC—glutaconyl-L-carnitine, C5-DC/C6-OH—glutaryl-L-carnitine/hydroxyhexanoyl-L-carnitine, C5-M-DC—methylglutaryl-L-carnitine, C6:1—hexenoyl-L-carnitine, C7-DC—pimelyl-L-carnitine, C8—octanoyl-L-carnitine, C8:1—octenoyl-L-carnitine, C9—nonayl-L-carnitine, LPC—lysophosphatidylcholine, PC—p [file pone.0211679.s003.docx]

| **amino acids** | sperm | seminal plasma |
| --- | --- | --- |
| Ala | 4.09 ± 4.86 | 634 ± 314 |
| Arg | 1.84 ± 3.22 | 895 ± 114 |
| Asn | 1.18 ± 1.92 | 1333 ± 496 |
| Asp | 0.31 ± 0.40 | 980 ± 494 |
| Cit | 0.29 ± 0.94 | 0.99 ± 0.91 |
| Gln | 21.1 ± 24.4 | 2893 ± 664 |
| Glu | 4.27 ± 2.22 | 3535 ± 1316 |
| Gly | 1.97 ± 3.01 | 2182 ± 607 |
| His | 0.32 ± 0.94 | 467 ± 106 |
| Ile | 2.15 ± 2.85 | 1349 ± 235 |
| Leu | 2.12 ± 4.93 | 1543 ± 270 |
| Lys | 0.82 ± 1.95 | 1860 ± 465 |
| Met | 0.015 ± 0.061 | 25.7 ± 10.4 |
| Orn | 0.11 ± 0.46 | 53.8 ± 16.8 |
| Phe | 0.62 ± 1.02 | 617 ± 117 |
| Pro | 1.39 ± 1.55 | 266 ± 116 |
| Ser | 7.60 ± 8.22 | 2438 ± 370 |
| Thr | 2.62 ± 3.20 | 2063 ± 664 |
| Trp | 0.061 ± 0.204 | 47.5 ± 24.4 |
| Tyr | 3.59 ± 5.18 | 1412 ± 176 |
| Val | 1.31 ± 2.09 | 1264 ± 359 |

| **biogenic amines** | sperm | seminal plasma |
| --- | --- | --- |
| Ac-Orn | 0 | 0.15 ± 0.21 |
| ADMA | 0.18 ± 0.24 | 0.32 ± 0.19 |
| alpha-AAA | 0 | 4.89 ± 3.07 |
| Carnosine | 0 | 7.50 ± 3.10 |
| Creatinine | 0 | 253 ± 67 |
| Histamine | 0 | 0 |
| Kynurenine | 0-101 ± 0.201 | 0.176 ± 0.061 |
| Met-SO | 0 | 0 |
| Nitro-Tyr | 0 | 0 |
| OH-Pro | 0 | 0.112 ± 0.086 |
| PEA | 0 | 0.021 ± 0.047 |
| Putrescine | 0.087 ± 0.106 | 95 ± 131 |
| Sarcosine | 0.24 ± 0.34 | 6.16 ± 2.29 |
| Serotonin | 0.040 ± 0.054 | 0.071 ± 0.039 |
| Spermidine | 0.20 ± 0.40 | 104 ± 35 |
| Spermine | 2.19 ± 4.20 | 1227 ± 184 |
| Taurine | 1.96 ± 2.50 | 357 ± 46 |
| total DMA | 0.041 ± 0.136 | 0.70 ± 0.63 |

| **acyl carnitines** | sperm | seminal plasma |
| --- | --- | --- |
| C0 | 8.51 ± 6.26 | 244 ± 135 |
| C2 | 32.4 ± 26.4 | 190 ± 80 |
| C3 | 0.84 ± 0.60 | 12.4 ± 3.9 |
| C3-DC | 0.061 ± 0.047 | 3.47 ± 1.10 |
| C3-OH | 0.025 ± 0.032 | 0.038 ± 0.022 |
| C3:1 | 0.010 ± 0.015 | 0.040 ± 0.005 |
| C4 | 0.109 ± 0.075 | 42.2 ± 18.8 |
| C4:1 | 0.015 ± 0.018 | 0.119 ± 0.029 |
| C5 | 0.18 ± 0.12 | 8.62 ± 2.69 |
| C5-DC | 0.014 ± 0.015 | 1.33 ± 0.41 |
| C5-M-DC | 0.021 ± 0.029 | 0.33 ± 0.23 |
| C5-OH (C3-DC-M) | 0.072 ± 0.058 | 1.55 ± 0.50 |
| C5:1 | 0.035 ± 0.034 | 0.71 ± 0.22 |
| C5:1-DC | 0.013 ± 0.018 | 0.72 ± 0.40 |
| C6 (C4:1-DC) | 0.017 ± 0.016 | 1.89 ± 0.70 |
| C6:1 | 0.011 ± 0.014 | 0.18 ± 0.04 |
| C7-DC | 0.035 ± 0.048 | 0.57 ± 0.21 |
| C8 | 0.086 ± 0.12 | 0.35 ± 0.08 |
| C9 | 0.016 ± 0.021 | 0.30 ± 0.26 |
| C10 | 0.042 ± 0.061 | 0.169 ± 0.036 |
| C10:1 | 0.029 ± 0.037 | 0.074 ± 0.022 |
| C10:2 | 0.16 ± 0.21 | 0.37 ± 0.04 |
| C12 | 0.031 ± 0.038 | 0.18 ± 0.07 |
| C12-DC | 0.097 ± 0.131 | 0.18 ± 0.01 |
| C12:1 | 0.043 ± 0.056 | 0.086 ± 0.012 |
| C14 | 0.053 ± 0.062 | 0.120 ± 0.025 |
| C14:1 | 0.045 ± 0.053 | 0.116 ± 0.020 |
| C14:1-OH | 0.042 ± 0.049 | 0.23 ± 0.09 |
| C14:2 | 0.060 ± 0.080 | 0.105 ± 0.021 |
| C14:2-OH | 0.036 ± 0.050 | 0.128 ± 0.031 |
| C16 | 0.045 ± 0.044 | 0.27 ± 0.09 |
| C16-OH | 0.018 ± 0.018 | 0.055 ± 0.008 |
| C16:1 | 0.034 ± 0.048 | 0.155 ± 0.030 |
| C16:1-OH | 0.018 ± 0.020 | 0.061 ± 0.014 |
| C16:2 | 0.010 ± 0.011 | 0.043 ± 0.010 |
| C16:2-OH | 0.013 ± 0.018 | 0.112 ± 0.033 |
| C18 | 0.025 ± 0.027 | 0.085 ± 0.052 |
| C18:1 | 0.013 ± 0.015 | 0.046 ± 0.027 |
| C18:1-OH | 0.018 ± 0.024 | 0.064 ± 0.029 |
| C18:2 | 0.014 ± 0.020 | 0.028 ± 0.009 |

| **lysophosphatidyl-**  **cholines** | sperm | seminal plasma |
| --- | --- | --- |
| LPC 14:0 | 0.72 ± 1.00 | 5.54 ± 2.29 |
| LPC 16:0 | 0.39 ± 0.36 | 13.6 ± 8.2 |
| LPC 16:1 | 0.081 ± 0.140 | 0.116 ± 0.039 |
| LPC 17:0 | 0.028 ± 0.039 | 0.183 ± 0.106 |
| LPC 18:0 | 0.114 ± 0.108 | 2.52 ± 1.49 |
| LPC 18:1 | 1.03 ± 1.85 | 2.01 ± 0.93 |
| LPC 18:2 | 0.096 ± 0.120 | 0.132 ± 0.046 |
| LPC 20:3 | 0.141 ± 0.201 | 0.119 ± 0.032 |
| LPC 20:4 | 0.027 ± 0.030 | 0.061 ± 0.033 |

| **phosphatidyl-cholines** | sperm | seminal plasma |
| --- | --- | --- |
| PC 24:0 | 0.082 ± 0.102 | 0.039 ± 0.014 |
| PC 26:0 | 0.63 ± 0.75 | 0.58 ± 0.16 |
| PC 28:1 | 3.01 ± 3.90 | 4.87 ± 0.76 |
| PC 30:0 | 0.31 ± 0.30 | 1.33 ± 0.56 |
| PC 30:2 | 0.014 ± 0.014 | 0.015 ± 0.018 |
| PC 32:0 | 0.73 ± 0.47 | 4.51 ± 1.92 |
| PC 32:1 | 0.28 ± 0.23 | 0.55 ± 0.29 |
| PC 32:2 | 0.074 ± 0.078 | 0.044 ± 0.030 |
| PC 32:3 | 0.048 ± 0.052 | 0.021 ± 0.008 |
| PC 34:1 | 1.70 ± 1.29 | 27.8 ± 12.7 |
| PC 34:2 | 1.94 ± 1.55 | 4.76 ± 2.41 |
| PC 34:3 | 0.167 ± 0.197 | 0.117 ± 0.061 |
| PC 34:4 | 0.026 ± 0.022 | 0.020 ± 0.008 |
| PC 36:0 | 0.56 ± 0.46 | 0.34 ± 0.24 |
| PC 36:1 | 0.39 ± 0.30 | 11.0 ± 5.1 |
| PC 36:2 | 1.02 ± 0.73 | 4.73 ± 2.26 |
| PC 36:3 | 2.30 ± 1.97 | 2.79 ± 1.23 |
| PC 36:4 | 0.47 ± 0.40 | 0.722 ± 0.33 |
| PC 36:5 | 0.118 ± 0.114 | 0.052 ± 0.028 |
| PC 36:6 | 0.074 ± 0.056 | 0.072 ± 0.039 |
| PC 38:0 | 0.24 ± 0.17 | 0.43 ± 0.20 |
| PC 38:1 | 0.077 ± 0.081 | 0.15 ± 0.15 |
| PC 38:3 | 0.70 ± 0.46 | 1.67 ± 0.75 |
| PC 38:4 | 0.31 ± 0.23 | 0.61 ± 0.23 |
| PC 38:5 | 0.35 ± 0.27 | 0.39 ± 0.24 |
| PC 38:6 | 3.25 ± 2.39 | 1.64 ± 1.26 |
| PC 40:1 | 0.28 ± 0.39 | 0.26 ± 0.06 |
| PC 40:2 | 0.046 ± 0.056 | 0.053 ± 0.037 |
| PC 40:3 | 0.101 ± 0.067 | 0.138 ± 0.068 |
| PC 40:4 | 0.085 ± 0.056 | 0.142 ± 0.068 |
| PC 40:5 | 0.082 ± 0.075 | 0.112 ± 0.064 |
| PC 40:6 | 0.90 ± 0.67 | 0.92 ± 0.42 |
| PC 42:0 | 0.057 ± 0.088 | 0.036 ± 0.010 |
| PC 42:1 | 0.022 ± 0.023 | 0.099 ± 0.015 |
| PC 42:2 | 0.031 ± 0.035 | 0.036 ± 0.015 |
| PC 42:4 | 0.091 ± 0.071 | 0.083 ± 0.046 |
| PC 42:5 | 0.079 ± 0.059 | 0.078 ± 0.048 |
| PC 42:6 | 0.36 ± 0.46 | 0.32 ± 0.08 |

| **1-acyl,2-alkyl phosphatidylcholines** | sperm | seminal plasma |
| --- | --- | --- |
| GPCe 30:0 | 0.20 ± 0.21 | 0.18 ± 0.04 |
| GPCe 30:1 | 0.068 ± 0.070 | 0.023 ± 0.021 |
| GPCe 30:2 | 0.037 ± 0.040 | 0.026 ± 0.006 |
| GPCe 32:1 | 0.117 ± 0.092 | 0.36 ± 0.20 |
| GPCe 32:2 | 0.096 ± 0.103 | 0.060 ± 0.022 |
| GPCe 34:0 | 0.091 ± 0.073 | 0.42 ± 0.18 |
| GPCe 34:1 | 0.173 ± 0.127 | 0.91 ± 0.40 |
| GPCe 34:2 | 0.102 ± 0.076 | 0.39 ± 0.04 |
| GPCe 34:3 | 0.073 ± 0.071 | 0.097 ± 0.042 |
| GPCe 36:0 | 0.101 ± 0.148 | 0.23 ± 0.06 |
| GPCe 36:1 | 0.174 ± 0.167 | 0.81 ± 0.38 |
| GPCe 36:2 | 0.111 ± 0.096 | 0.25 ± 0.12 |
| GPCe 36:3 | 0.057 ± 0.043 | 0.133 ± 0.056 |
| GPCe 36:4 | 0.064 ± 0.050 | 0.131 ± 0.045 |
| GPCe 36:5 | 0.066 ± 0.058 | 0.080 ± 0.029 |
| GPCe 38:0 | 0.22 ± 0.32 | 0.31 ± 0.07 |
| GPCe 38:1 | 0.034 ± 0.040 | 0.052 ± 0.056 |
| GPCe 38:2 | 0.055 ± 0.052 | 0.069 ± 0.039 |
| GPCe 38:3 | 0.084 ± 0.067 | 0.123 ± 0.059 |
| GPCe 38:4 | 0.084 ± 0.062 | 0.117 ± 0.049 |
| GPCe 38:5 | 0.176 ± 0.173 | 0.168 ± 0.095 |
| GPCe 38:6 | 1.36 ± 0.97 | 1.38 ± 0.97 |
| GPCe 40:1 | 0.057 ± 0.066 | 0.040 ± 0.025 |
| GPCe 40:2 | 0.062 ± 0.062 | 0.139 ± 0.056 |
| GPCe 40:3 | 0.044 ± 0.035 | 0.054 ± 0.021 |
| GPCe 40:4 | 0.060 ± 0.063 | 0.069 ± 0.018 |
| GPCe 40:5 | 0.051 ± 0.039 | 0.065 ± 0.028 |
| GPCe 40:6 | 0.159 ± 0.120 | 0.148 ± 0.079 |
| GPCe 42:0 | 0.34 ± 0.43 | 0.31 ± 0.06 |
| GPCe 42:1 | 0.074 ± 0.097 | 0.072 ± 0.015 |
| GPCe 42:2 | 0.039 ± 0.045 | 0.038 ± 0.010 |
| GPCe 42:3 | 0.090 ± 0.078 | 0.098 ± 0.029 |
| GPCe 42:4 | 0.008 ± 0.012 | 0.009 ± 0.008 |
| GPCe 42:5 | 0.28 ± 0.40 | 0.26 ± 0.03 |
| GPCe 44:3 | 0.057 ± 0.073 | 0.137 ± 0.060 |
| GPCe 44:4 | 0.077 ± 0.121 | 0.092 ± 0.014 |
| GPCe 44:5 | 0.074 ± 0.112 | 0.043 ± 0.018 |
| GPCe 44:6 | 0.039 ± 0.048 | 0.032 ± 0.010 |

| **sphingomyelins hydroxylated SM** | sperm | seminal plasma |
| --- | --- | --- |
| SM (OH) 14:1 | 0.23 ± 0.18 | 1.82 ± 0.97 |
| SM (OH) 16:1 | 0.113 ± 0.095 | 1.32 ± 0.70 |
| SM (OH) 22:1 | 0.082 ± 0.079 | 2.14 ± 0.93 |
| SM (OH) 22:2 | 0.069 ± 0.059 | 0.60 ± 0.29 |
| SM (OH) 24:1 | 0.026 ± 0.028 | 0.55 ± 0.20 |
| SM 16:0 | 4.44 ± 3.97 | 104 ± 54 |
| SM 16:1 | 0.34 ± 0.27 | 2.43 ± 1.27 |
| SM 18:0 | 0.55 ± 0.52 | 14.9 ± 8.5 |
| SM 18:1 | 0.184 ± 0.152 | 1.63 ± 0.97 |
| SM 20:2 | 0.011 ± 0.016 | 0.030 ± 0.015 |
| SM 22:3 | 0.009 ± 0.018 | 0.020 ± 0.015 |
| SM 24:0 | 0.36 ± 0.33 | 18.6 ± 9.6 |
| SM 24:1 | 0.78 ± 0.64 | 10.6 ± 5.3 |
| SM 26:0 | 0.021 ± 0.021 | 0.61 ± 0.27 |
| SM 26:1 | 0.177 ± 0.143 | 0.59 ± 0.31 |

| **sugars** | sperm | seminal plasma |
| --- | --- | --- |
| hexoses | 77.6 ± 87.2 | 31436 ± 15287 |
