# Supplementary material for: Metabolomic profiling reveals correlations between spermiogram parameters and the metabolites present in human spermatozoa and seminal plasma
Source: PLoS One. 2019 Feb 20;14(2):e0211679. doi: 10.1371/journal.pone.0211679 (PMC6382115; doi:10.1371/journal.pone.0211679)
Supplement: S3 Table — Data are Spearman correlation rank coefficients. Significant correlations are highlighted in bolt red. Abbreviations: Ala—alanine, Arg—arginine, Asn—asparagine, Gln—glutamine, Glu—glutamate, Ile—isoleucine, Leu—leucine, Phe—phenylalanine, Pro—proline, Ser—serine, Thr—threonine, Tyr—tyrosine. (DOCX) [file pone.0211679.s004.docx]

|  | Arg | Asn | Gln | Glu | Ile | Leu | Phe | Pro | Ser | Thr | Tyr |
| --- | --- | --- | --- | --- | --- | --- | --- | --- | --- | --- | --- |
| Ala | 0.389 | **0.551** | 0.209 | **0.946** | **0.610** | 0.435 | **0.611** | **0.950** | **0.601** | **0.841** | **0.616** |
| Arg |  | **0.871** | 0.420 | **0.486** | **0.839** | **0.885** | **0.844** | 0.441 | **0.870** | **0.685** | **0.873** |
| Asn |  |  | 0.351 | **0.634** | **0.896** | **0.788** | **0.861** | **0.527** | **0.940** | **0.743** | **0.928** |
| Gln |  |  |  | 0.198 | **0.549** | **0.630** | **0.646** | 0.279 | **0.519** | **0.470** | **0.498** |
| Glu |  |  |  |  | **0.654** | **0.518** | **0.645** | **0.917** | **0.660** | **0.901** | **0.664** |
| Ile |  |  |  |  |  | **0.900** | **0.935** | **0.643** | **0.977** | **0.813** | **0.983** |
| Leu |  |  |  |  |  |  | **0.914** | **0.482** | **0.884** | **0.750** | **0.876** |
| Phe |  |  |  |  |  |  |  | **0.626** | **0.951** | **0.835** | **0.927** |
| Pro |  |  |  |  |  |  |  |  | **0.619** | **0.862** | **0.639** |
| Ser |  |  |  |  |  |  |  |  |  | **0.813** | **0.986** |
| Thr |  |  |  |  |  |  |  |  |  |  | **0.806** |
