# Supplementary material for: Metabolomic profiling reveals correlations between spermiogram parameters and the metabolites present in human spermatozoa and seminal plasma
Source: PLoS One. 2019 Feb 20;14(2):e0211679. doi: 10.1371/journal.pone.0211679 (PMC6382115; doi:10.1371/journal.pone.0211679)
Supplement: S4 Table — Data are Spearman correlation rank coefficients. Significant correlations are highlighted in bolt. Abbreviations: Ala—alanine, Arg—arginine, Asn—asparagine, Asp—aspartate, Cit—citrulline, Gln—glutamine, Glu—glutamate, Gly—glycine, His—histidine, Ile—isoleucine, Leu—leucine, Lys—lysine, Met—methionine, Orn—ornithine, Phe—phenylalanine, Pro—proline, Ser—serine, Thr—threonine, Trp—tryptophan, Tyr—tyrosine, Val—valine. (DOCX) [file pone.0211679.s005.docx]

|  | Arg | Asn | Asp | Cit | Gln | Glu | Gly | His | Ile | Leu | Lys | Met | Orn | Phe | Pro | Ser | Thr | Trp | Tyr | Val |
| --- | --- | --- | --- | --- | --- | --- | --- | --- | --- | --- | --- | --- | --- | --- | --- | --- | --- | --- | --- | --- |
| Ala | **0.489** | **0.889** | **0.959** | 0.368 | **0.854** | **0.941** | **0.830** | 0.217 | **0.660** | **0.680** | **0.921** | **0.970** | **0.678** | **0.585** | **0.919** | **0.706** | **0.817** | **0.936** | 0.342 | **0.845** |
| Arg |  | **0.714** | **0.488** | -0.140 | **0.768** | **0.586** | **0.764** | 0.388 | **0.862** | **0.803** | **0.682** | **0.516** | 0.185 | **0.838** | **0.465** | **0.892** | **0.799** | **0.587** | **0.688** | **0.795** |
| Asn |  |  | **0.876** | 0.162 | **0.974** | **0.943** | **0.941** | 0.319 | **0.873** | **0.888** | **0.968** | **0.872** | **0.510** | **0.839** | **0.790** | **0.898** | **0.958** | **0.821** | **0.559** | **0.979** |
| Asp |  |  |  | 0.389 | **0.842** | **0.940** | **0.815** | 0.182 | **0.633** | **0.662** | **0.912** | **0.933** | **0.691** | **0.580** | **0.892** | **0.674** | **0.784** | **0.897** | 0.313 | **0.835** |
| Cit |  |  |  |  | 0.166 | 0.342 | 0.211 | 0.026 | -0.072 | -0.027 | 0.292 | 0.377 | **0.698** | -0.082 | 0.365 | 0.068 | 0.100 | 0.377 | -0.012 | 0.192 |
| Gln |  |  |  |  |  | **0.920** | **0.971** | 0.360 | **0.865** | **0.883** | **0.955** | **0.824** | **0.535** | **0.856** | **0.768** | **0.919** | **0.942** | **0.803** | **0.580** | **0.973** |
| Glu |  |  |  |  |  |  | **0.910** | 0.273 | **0.771** | **0.811** | **0.973** | **0.904** | **0.605** | **0.727** | **0.856** | **0.830** | **0.903** | **0.871** | **0.475** | **0.931** |
| Gly |  |  |  |  |  |  |  | 0.349 | **0.841** | **0.848** | **0.955** | **0.798** | **0.513** | **0.838** | **0.736** | **0.924** | **0.951** | **0.800** | **0.588** | **0.964** |
| His |  |  |  |  |  |  |  |  | 0.416 | 0.363 | 0.313 | 0.153 | 0.372 | **0.473** | 0.196 | **0.471** | 0.335 | 0.210 | 0.409 | 0.378 |
| Ile |  |  |  |  |  |  |  |  |  | **0.965** | **0.812** | **0.655** | 0.240 | **0.913** | **0.629** | **0.954** | **0.936** | **0.658** | **0.763** | **0.915** |
| Leu |  |  |  |  |  |  |  |  |  |  | **0.823** | **0.654** | 0.300 | **0.924** | **0.622** | **0.938** | **0.919** | **0.622** | **0.733** | **0.919** |
| Lys |  |  |  |  |  |  |  |  |  |  |  | **0.907** | **0.582** | **0.771** | **0.844** | **0.882** | **0.942** | **0.887** | **0.527** | **0.961** |
| Met |  |  |  |  |  |  |  |  |  |  |  |  | **0.654** | **0.595** | **0.940** | **0.691** | **0.804** | **0.958** | 0.332 | **0.831** |
| Orn |  |  |  |  |  |  |  |  |  |  |  |  |  | 0.258 | **0.643** | 0.362 | 0.388 | **0.635** | 0.062 | **0.500** |
| Phe |  |  |  |  |  |  |  |  |  |  |  |  |  |  | **0.546** | **0.921** | **0.871** | **0.560** | **0.690** | **0.883** |
| Pro |  |  |  |  |  |  |  |  |  |  |  |  |  |  |  | **0.637** | **0.728** | **0.949** | 0.359 | **0.762** |
| Ser |  |  |  |  |  |  |  |  |  |  |  |  |  |  |  |  | **0.960** | **0.714** | **0.741** | **0.955** |
| Thr |  |  |  |  |  |  |  |  |  |  |  |  |  |  |  |  |  | **0.794** | **0.655** | **0.977** |
| Trp |  |  |  |  |  |  |  |  |  |  |  |  |  |  |  |  |  |  | 0.387 | **0.816** |
| Tyr |  |  |  |  |  |  |  |  |  |  |  |  |  |  |  |  |  |  |  | **0.645** |
