# Supplementary material for: Metabolomic profiling reveals correlations between spermiogram parameters and the metabolites present in human spermatozoa and seminal plasma
Source: PLoS One. 2019 Feb 20;14(2):e0211679. doi: 10.1371/journal.pone.0211679 (PMC6382115; doi:10.1371/journal.pone.0211679)
Supplement: S5 Table — Data are Spearman correlation rank coefficients. Significant correlations are highlighted in bolt. Abbreviations: Ala—alanine, Arg—arginine, Asn—asparagine, Asp—aspartate, Cit—citrulline, Gln—glutamine, Glu—glutamate, Gly—glycine, His—histidine, Ile—isoleucine, Leu—leucine, Lys—lysine, Met—methionine, Orn—ornithine, Phe—phenylalanine, Pro—proline, Ser—serine, Thr—threonine, Trp—tryptophan, Tyr—tyrosine, Val—valine. (DOCX) [file pone.0211679.s006.docx]

| sperm  SP | Ala | Arg | Asn | Gln | Glu | Ile | Leu | Phe | Pro | Ser | Thr | Tyr |
| --- | --- | --- | --- | --- | --- | --- | --- | --- | --- | --- | --- | --- |
| Ala | -0.173 | -0.246 | -0.174 | 0.025 | -0.040 | -0.178 | -0.127 | -0.135 | -0.217 | -0.166 | 0.029 | -0.186 |
| Arg | -0.224 | -0.070 | 0.010 | **0.477** | -0.162 | 0.149 | 0.201 | 0.109 | -0.234 | 0.136 | 0.018 | 0.090 |
| Asn | -0.162 | -0.176 | -0.060 | 0.270 | -0.060 | 0.017 | 0.047 | 0.053 | -0.202 | 0.003 | 0.063 | -0.027 |
| Asp | -0.159 | -0.269 | -0.232 | 0.099 | -0.068 | -0.210 | -0.157 | -0.127 | -0.202 | -0.191 | 0.032 | -0.214 |
| Cit | -0.417 | -0.227 | -0.400 | -0.282 | -0.435 | **-0.518** | -0.296 | -0.361 | -0.438 | **-0.492** | -0.303 | **-0.496** |
| Gln | -0.247 | -0.198 | -0.089 | 0.321 | -0.146 | -0.014 | 0.020 | 0.023 | -0.284 | -0.026 | -0.005 | -0.054 |
| Glu | -0.195 | -0.164 | -0.088 | 0.183 | -0.105 | -0.056 | -0.020 | -0.002 | -0.257 | -0.057 | 0.038 | -0.074 |
| Gly | -0.275 | -0.202 | -0.088 | 0.211 | -0.171 | -0.032 | -0.009 | -0.020 | -0.340 | -0.045 | -0.041 | -0.069 |
| His | -0.291 | 0.070 | -0.073 | 0.261 | -0.206 | -0.042 | 0.102 | -0.108 | -0.273 | -0.113 | -0.160 | -0.053 |
| Ile | -0.183 | 0.046 | 0.153 | **0.454** | -0.068 | 0.260 | 0.298 | 0.214 | -0.183 | 0.231 | 0.135 | 0.209 |
| Leu | -0.134 | 0.079 | 0.192 | **0.512** | -0.039 | 0.276 | 0.316 | 0.287 | -0.164 | 0.267 | 0.165 | 0.247 |
| Lys | -0.206 | -0.218 | -0.141 | 0.192 | -0.104 | -0.069 | -0.021 | -0.030 | -0.256 | -0.086 | 0.029 | -0.111 |
| Met | -0.099 | -0.230 | -0.158 | 0.083 | 0.024 | -0.133 | -0.063 | -0.082 | -0.128 | -0.133 | 0.120 | -0.170 |
| Orn | -0.341 | -0.185 | -0.343 | -0.012 | -0.265 | -0.405 | -0.193 | -0.288 | -0.371 | -0.393 | -0.162 | -0.386 |
| Phe | -0.109 | 0.028 | 0.171 | **0.568** | -0.034 | 0.246 | 0.261 | 0.256 | -0.144 | 0.223 | 0.139 | 0.193 |
| Pro | -0.120 | -0.236 | -0.192 | 0.163 | 0.008 | -0.147 | -0.044 | -0.107 | -0.147 | -0.170 | 0.121 | -0.186 |
| Ser | -0.277 | -0.055 | 0.042 | 0.382 | -0.184 | 0.127 | 0.179 | 0.105 | -0.316 | 0.103 | 0.005 | 0.078 |
| Thr | -0.178 | -0.108 | 0.042 | 0.246 | -0.074 | 0.109 | 0.107 | 0.079 | -0.225 | 0.088 | 0.075 | 0.064 |
| Trp | -0.206 | -0.315 | -0.247 | 0.029 | -0.084 | -0.224 | -0.130 | -0.206 | -0.259 | -0.230 | 0.003 | -0.264 |
| Tyr | -0.161 | 0.251 | 0.275 | **0.476** | -0.059 | 0.311 | **0.448** | 0.337 | -0.198 | 0.313 | 0.167 | 0.273 |
| Val | -0.220 | -0.127 | -0.023 | 0.300 | -0.120 | 0.041 | 0.100 | 0.070 | -0.273 | 0.030 | 0.039 | 0.000 |
