# Supplementary material for: Metabolomic profiling reveals correlations between spermiogram parameters and the metabolites present in human spermatozoa and seminal plasma
Source: PLoS One. 2019 Feb 20;14(2):e0211679. doi: 10.1371/journal.pone.0211679 (PMC6382115; doi:10.1371/journal.pone.0211679)
Supplement: S7 Table — Data are Spearman correlation rank coefficients. Significant positive correlations are highlighted in red, significant negative correlations in blue. Abbreviations: Ala—alanine, Arg—arginine, Asn—asparagine, Asp—aspartate, Cit—citrulline, Gln—glutamine, Glu—glutamate, Gly—glycine, His—histidine, Ile—isoleucine, Leu—leucine, Lys—lysine, Met—methionine, Orn—ornithine, Phe—phenylalanine, Pro—proline, Ser—serine, Thr—threonine, Trp—tryptophan, Tyr—tyrosine, Val—valine, ADMA—asymmetrically dimethylated arginine, alpha-AAA—α-aminoadipic acid, OH-Pro—hydroxyproline, DMA—dimethylamine, C0—DL-carnitine, C10—decanoyl-L-carnitine, C10:1—decenoyl-L-carnitine, C10:2—decadienyl-L-carnitine, C12—dodecanoyl-L-carnitine, C12:1—dodecenoyl-L-carnitine, C12-DC—dodecanedioyl-L-carnitine, C14—tetradecanoyl-L-carnitine, C14:1—tetradecenoyl-L-carnitine, C14:1-OH—hydroxytetradecenoyl-L-carnitine, C14:2—tetradecadienyl-L-carnitine, C14:2-OH—hydroxytetradecadienyl-L-carnitine, C16—hexadecanoyl-L-carnitine, C16:1—hexadecenoyl-L-carnitine, C16:1-OH—hydroxyhexadecenoyl-L-carnitine, C16:2—hexadecadienyl-L-carnitine, C16:2-OH—hydroxyhexadecadienyl-L-carnitine, C16-OH—hydroxyhexadecanoyl-L-carnitine, C18—octadecanoyl-L-carnitine, C18:1—octadecenoyl-L-carnitine, C18:1-OH—hydroxyoctadecenoyl-L-carnitine, C18:2—octadecadienyl-L-carnitine, C2—acetyl-L-carnitine, C3—propionyl-L-carnitine, C3:1—propenyl-L-carnitine, C3-DC/C4-OH—malonyl-L-carnitine/hydroxybutyryl-L-carnitine, C3-DC-M/C5-OH—methylmalonyl-L-carnitine/hydroxyvaleryl-L-carnitine, C3-OH—hydroxypropionyl-L-carnitine, C4—butyryl-L-carnitine, C4:1—butenyl-L-carnitine, C4:1-DC/C6—fumaryl-L-carnitine/hexanoyl-L-carnitine, C5—valeryl-L-carnitine, C5:1—tiglyl-L-carnitine, C5:1-DC—glutaconyl-L-carnitine, C5-DC/C6-OH—glutaryl-L-carnitine/hydroxyhexanoyl-L-carnitine, C5-M-DC—methylglutaryl-L-carnitine, C6:1—hexenoyl-L-carnitine, C7-DC—pimelyl-L-carnitine, C8—octanoyl-L-carnitine, C8:1—octenoyl-L-carnitine, C9—nonayl-L-carnitine, LPC—lysophosphatidylchol [file pone.0211679.s008.docx]

| **seminal plasma** | abstention time (d) | c (mio/ml) | V (ml) | motility | | | Morphology | | | | |
| --- | --- | --- | --- | --- | --- | --- | --- | --- | --- | --- | --- |
|  |  |  |  | progressive | local | non-motile | normal | head | neck/ midpiece | tail | cytopl. Droplets |
| Ala | 0.192 | 0.314 | 0.172 | -0.090 | 0.470 | -0.228 | 0.149 | 0.220 | -0.359 | 0.025 | -0.249 |
| Arg | -0.072 | 0.661 | 0.642 | 0.137 | -0.062 | -0.055 | 0.122 | 0.227 | -0.394 | -0.003 | -0.140 |
| Asn | 0.057 | 0.507 | 0.321 | -0.008 | 0.300 | -0.180 | 0.212 | 0.250 | -0.409 | -0.051 | -0.128 |
| Asp | 0.187 | 0.406 | 0.067 | -0.012 | 0.398 | -0.255 | 0.262 | 0.119 | -0.331 | 0.047 | -0.167 |
| Cit | 0.613 | 0.223 | -0.244 | -0.248 | 0.089 | 0.050 | -0.123 | 0.212 | -0.237 | -0.067 | -0.035 |
| Gln | 0.096 | 0.565 | 0.417 | 0.015 | 0.243 | -0.167 | 0.198 | 0.306 | -0.444 | -0.155 | -0.105 |
| Glu | 0.147 | 0.487 | 0.191 | -0.003 | 0.339 | -0.229 | 0.120 | 0.248 | -0.382 | -0.047 | -0.109 |
| Gly | 0.141 | 0.568 | 0.405 | -0.039 | 0.163 | -0.058 | 0.117 | 0.418 | -0.538 | -0.241 | -0.033 |
| His | -0.237 | 0.339 | 0.255 | -0.441 | 0.275 | 0.290 | -0.037 | 0.348 | -0.301 | -0.372 | -0.394 |
| Ile | -0.177 | 0.599 | 0.524 | 0.116 | 0.186 | -0.187 | 0.158 | 0.210 | -0.326 | -0.054 | -0.071 |
| Leu | -0.174 | 0.597 | 0.428 | 0.179 | 0.193 | -0.236 | 0.100 | 0.241 | -0.337 | -0.074 | -0.096 |
| Lys | 0.208 | 0.514 | 0.285 | -0.032 | 0.286 | -0.154 | 0.129 | 0.349 | -0.498 | -0.108 | -0.133 |
| Met | 0.282 | 0.335 | 0.166 | -0.049 | 0.367 | -0.243 | 0.110 | 0.237 | -0.371 | 0.086 | -0.287 |
| Orn | 0.329 | 0.335 | -0.148 | -0.199 | 0.324 | -0.031 | -0.007 | 0.394 | -0.484 | -0.115 | -0.457 |
| Phe | -0.099 | 0.612 | 0.469 | 0.020 | 0.044 | -0.041 | 0.042 | 0.288 | -0.327 | -0.162 | -0.100 |
| Pro | 0.224 | 0.369 | 0.187 | -0.005 | 0.400 | -0.279 | 0.111 | 0.244 | -0.305 | -0.076 | -0.283 |
| Ser | -0.013 | 0.648 | 0.528 | 0.009 | 0.134 | -0.062 | 0.051 | 0.351 | -0.454 | -0.127 | -0.104 |
| Thr | 0.019 | 0.547 | 0.423 | 0.004 | 0.206 | -0.111 | 0.096 | 0.298 | -0.427 | -0.060 | -0.043 |
| Trp | 0.277 | 0.377 | 0.290 | -0.105 | 0.373 | -0.150 | 0.076 | 0.251 | -0.365 | 0.024 | -0.297 |
| Tyr | -0.180 | 0.606 | 0.398 | 0.133 | 0.093 | -0.118 | 0.207 | 0.241 | -0.318 | -0.327 | 0.044 |
| Val | 0.050 | 0.580 | 0.398 | -0.011 | 0.243 | -0.135 | 0.133 | 0.297 | -0.436 | -0.086 | -0.129 |
| ADMA | -0.098 | -0.179 | -0.317 | -0.337 | 0.257 | 0.302 | 0.125 | 0.094 | -0.045 | -0.488 | 0.285 |
| alpha-AAA | 0.387 | -0.059 | -0.146 | -0.262 | 0.485 | -0.116 | -0.158 | 0.146 | -0.166 | -0.006 | -0.359 |
| Carnosine | -0.026 | 0.471 | 0.298 | -0.290 | 0.230 | 0.192 | -0.160 | 0.638 | -0.576 | -0.446 | -0.337 |
| Creatinine | -0.352 | -0.160 | 0.302 | -0.360 | 0.038 | 0.457 | 0.056 | -0.346 | 0.414 | 0.095 | 0.191 |
| Kynurenine | 0.235 | 0.207 | -0.369 | -0.215 | 0.243 | 0.112 | -0.101 | 0.298 | -0.406 | 0.024 | -0.320 |
| OH-Pro | -0.180 | 0.438 | 0.431 | 0.119 | -0.231 | 0.092 | -0.215 | 0.190 | -0.086 | -0.101 | -0.171 |
| Putrescine | -0.497 | -0.149 | -0.087 | -0.094 | 0.370 | 0.023 | -0.097 | -0.124 | 0.190 | 0.022 | -0.193 |
| Sarcosine | 0.294 | -0.331 | -0.259 | -0.176 | 0.169 | 0.019 | -0.416 | 0.373 | -0.165 | -0.288 | -0.365 |
| Serotonin | 0.221 | 0.208 | 0.005 | -0.290 | 0.177 | 0.327 | -0.010 | 0.435 | -0.433 | -0.590 | 0.073 |
| Spermidine | 0.094 | 0.090 | -0.177 | 0.011 | 0.207 | -0.260 | 0.246 | 0.082 | -0.153 | -0.110 | -0.304 |
| Spermine | 0.010 | 0.160 | -0.260 | 0.176 | 0.123 | -0.269 | 0.053 | 0.386 | -0.473 | -0.165 | -0.376 |
| Taurine | -0.143 | 0.011 | 0.130 | -0.080 | 0.434 | -0.145 | -0.010 | 0.471 | -0.388 | -0.433 | -0.267 |
| total DMA | 0.269 | -0.332 | 0.096 | -0.354 | 0.499 | 0.077 | 0.046 | 0.078 | -0.077 | -0.337 | 0.057 |
| C0 | 0.105 | 0.272 | 0.026 | -0.332 | 0.321 | 0.152 | -0.076 | 0.295 | -0.321 | -0.276 | -0.111 |
| C10 | -0.065 | 0.071 | 0.467 | -0.188 | -0.249 | 0.451 | -0.390 | -0.008 | 0.238 | -0.088 | -0.022 |
| C10:1 | -0.534 | 0.008 | 0.020 | -0.429 | 0.150 | 0.424 | 0.219 | -0.219 | 0.210 | -0.187 | 0.137 |
| C10:2 | -0.304 | 0.083 | 0.313 | 0.017 | -0.075 | 0.124 | -0.449 | 0.158 | 0.046 | -0.211 | -0.139 |
| C12 | -0.264 | 0.322 | 0.428 | -0.228 | 0.151 | 0.124 | 0.031 | 0.028 | 0.026 | -0.319 | 0.244 |
| C12-DC | -0.412 | 0.227 | 0.047 | -0.286 | 0.125 | 0.366 | 0.056 | 0.276 | -0.244 | -0.317 | -0.051 |
| C12:1 | -0.396 | -0.047 | 0.002 | -0.372 | 0.212 | 0.297 | -0.090 | 0.252 | -0.130 | -0.268 | -0.205 |
| C14 | -0.544 | -0.039 | -0.034 | -0.062 | -0.082 | 0.077 | 0.011 | -0.391 | 0.525 | 0.140 | -0.031 |
| C14:1 | -0.554 | -0.035 | 0.084 | -0.026 | 0.031 | 0.205 | -0.120 | -0.225 | 0.390 | -0.015 | -0.239 |
| C14:1-OH | -0.438 | 0.295 | 0.133 | 0.225 | -0.301 | 0.069 | 0.028 | -0.168 | 0.206 | -0.106 | 0.286 |
| C14:2 | -0.447 | 0.245 | 0.241 | 0.270 | -0.144 | -0.035 | -0.185 | -0.178 | 0.247 | 0.042 | 0.031 |
| C14:2-OH | -0.228 | 0.471 | 0.104 | 0.429 | -0.388 | -0.079 | -0.110 | 0.176 | -0.147 | -0.122 | 0.087 |
| C16 | -0.222 | 0.406 | -0.027 | 0.173 | 0.125 | -0.263 | 0.271 | -0.228 | 0.096 | 0.204 | -0.330 |
| C16-OH | 0.210 | 0.289 | 0.050 | -0.321 | 0.228 | 0.214 | -0.133 | 0.548 | -0.477 | -0.563 | -0.342 |
| C16:1 | -0.212 | 0.156 | 0.494 | -0.241 | 0.181 | 0.215 | -0.345 | 0.079 | 0.183 | -0.195 | -0.336 |
| C16:1-OH | -0.269 | 0.340 | 0.391 | -0.228 | 0.120 | 0.256 | -0.245 | 0.428 | -0.347 | -0.358 | -0.091 |
| C16:2 | -0.019 | 0.489 | 0.693 | 0.057 | 0.209 | -0.122 | 0.006 | 0.228 | -0.313 | -0.055 | -0.192 |
| C16:2-OH | -0.079 | 0.322 | 0.609 | 0.127 | 0.082 | -0.105 | -0.055 | 0.155 | -0.153 | -0.131 | -0.065 |
| C18 | -0.089 | 0.283 | -0.152 | -0.062 | 0.452 | -0.198 | 0.198 | 0.033 | -0.175 | 0.100 | -0.516 |
| C18:1 | -0.158 | 0.269 | -0.197 | -0.107 | 0.305 | -0.081 | 0.214 | -0.003 | -0.148 | 0.104 | -0.560 |
| C18:1-OH | -0.231 | 0.579 | 0.306 | 0.352 | -0.589 | 0.033 | 0.092 | 0.119 | -0.207 | -0.150 | 0.239 |
| C18:2 | -0.279 | 0.090 | 0.124 | -0.094 | 0.056 | -0.036 | 0.104 | -0.483 | 0.424 | 0.299 | -0.009 |
| C2 | -0.214 | 0.501 | -0.089 | 0.097 | -0.121 | 0.179 | 0.131 | 0.063 | -0.226 | -0.036 | 0.114 |
| C3 | -0.329 | 0.639 | 0.108 | 0.071 | 0.102 | -0.137 | 0.155 | -0.088 | -0.090 | 0.140 | -0.139 |
| C3-DC (C4-OH) | 0.076 | 0.441 | -0.023 | 0.113 | 0.079 | -0.050 | -0.041 | 0.226 | -0.363 | -0.008 | 0.014 |
| C3-OH | -0.002 | 0.015 | 0.402 | 0.175 | 0.201 | -0.311 | 0.199 | 0.055 | -0.191 | -0.051 | 0.116 |
| C3:1 | -0.067 | 0.290 | 0.432 | 0.025 | 0.136 | 0.042 | -0.160 | 0.229 | -0.103 | -0.347 | 0.200 |
| C4 | -0.231 | 0.241 | -0.043 | -0.083 | -0.049 | 0.244 | -0.197 | -0.018 | 0.060 | 0.095 | -0.302 |
| C4:1 | -0.339 | 0.298 | 0.261 | 0.076 | -0.171 | 0.182 | -0.260 | 0.171 | -0.125 | -0.223 | -0.003 |
| C5 | -0.066 | 0.456 | -0.014 | -0.198 | 0.159 | 0.083 | -0.264 | 0.157 | -0.150 | 0.131 | -0.424 |
| C5-DC (C6-OH) | -0.109 | 0.531 | 0.129 | -0.012 | -0.025 | 0.221 | -0.254 | 0.348 | -0.331 | -0.196 | 0.012 |
| C5-M-DC | -0.135 | 0.320 | 0.236 | 0.083 | -0.033 | 0.058 | -0.014 | 0.084 | -0.273 | 0.055 | 0.176 |
| C5-OH (C3-DC-M) | -0.260 | 0.636 | 0.144 | -0.099 | 0.059 | 0.067 | 0.002 | 0.271 | -0.368 | -0.198 | -0.263 |
| C5:1 | 0.009 | 0.397 | 0.096 | -0.164 | 0.202 | 0.045 | -0.243 | 0.149 | -0.165 | 0.070 | -0.397 |
| C5:1-DC | -0.169 | 0.633 | 0.155 | -0.026 | -0.082 | 0.210 | -0.291 | 0.434 | -0.360 | -0.286 | -0.062 |
| C6 (C4:1-DC) | 0.182 | 0.550 | 0.087 | 0.074 | 0.139 | -0.057 | 0.093 | 0.238 | -0.379 | 0.011 | -0.245 |
| C6:1 | 0.070 | 0.592 | 0.279 | 0.111 | -0.057 | 0.081 | -0.095 | 0.281 | -0.389 | -0.003 | 0.036 |
| C7-DC | -0.041 | 0.289 | -0.082 | -0.191 | 0.167 | 0.311 | -0.364 | 0.357 | -0.272 | -0.245 | -0.104 |
| C8 | 0.009 | 0.278 | -0.010 | -0.235 | 0.347 | 0.107 | -0.078 | 0.029 | -0.068 | -0.080 | -0.193 |
| C9 | -0.126 | 0.347 | 0.320 | -0.252 | 0.196 | 0.312 | -0.270 | 0.509 | -0.400 | -0.453 | -0.143 |
| LPC 14:0 | 0.119 | -0.098 | 0.151 | 0.270 | 0.253 | -0.340 | 0.111 | 0.184 | -0.360 | -0.006 | -0.174 |
| LPC 16:0 | 0.141 | 0.260 | -0.222 | -0.059 | 0.449 | -0.256 | 0.132 | 0.158 | -0.319 | 0.099 | -0.369 |
| LPC 16:1 | 0.279 | 0.212 | -0.396 | -0.029 | 0.166 | -0.134 | 0.149 | 0.232 | -0.384 | 0.115 | -0.247 |
| LPC 17:0 | 0.079 | 0.189 | -0.358 | -0.094 | 0.376 | -0.202 | 0.186 | 0.080 | -0.252 | 0.139 | -0.276 |
| LPC 18:0 | 0.107 | 0.260 | -0.182 | -0.022 | 0.483 | -0.269 | 0.136 | 0.099 | -0.279 | 0.192 | -0.412 |
| LPC 18:1 | 0.224 | 0.352 | -0.254 | -0.134 | 0.307 | -0.085 | 0.120 | 0.266 | -0.427 | 0.017 | -0.242 |
| LPC 18:2 | 0.079 | 0.206 | -0.377 | -0.049 | -0.025 | 0.019 | 0.115 | 0.293 | -0.369 | -0.080 | -0.287 |
| LPC 20:3 | 0.141 | 0.332 | -0.066 | -0.167 | -0.121 | 0.289 | -0.048 | 0.275 | -0.338 | 0.012 | -0.270 |
| LPC 20:4 | 0.002 | -0.042 | -0.282 | -0.100 | 0.024 | 0.114 | 0.043 | 0.204 | -0.186 | -0.008 | -0.105 |
| PC 24:0 | 0.023 | 0.065 | -0.023 | 0.068 | 0.201 | -0.050 | -0.054 | 0.375 | -0.378 | -0.174 | 0.163 |
| PC 26:0 | 0.245 | -0.023 | -0.430 | 0.043 | -0.182 | 0.031 | 0.184 | 0.428 | -0.540 | -0.295 | 0.152 |
| PC 28:1 | 0.272 | 0.161 | -0.057 | 0.094 | 0.384 | -0.374 | 0.170 | 0.059 | -0.289 | 0.259 | -0.217 |
| PC 30:0 | 0.177 | 0.332 | -0.064 | -0.125 | 0.452 | -0.114 | 0.200 | 0.022 | -0.243 | 0.148 | -0.190 |
| PC 32:0 | 0.117 | 0.319 | -0.046 | -0.174 | 0.455 | -0.071 | 0.251 | -0.071 | -0.104 | 0.074 | -0.221 |
| PC 32:1 | -0.199 | 0.335 | -0.268 | 0.144 | -0.018 | 0.023 | 0.047 | 0.198 | -0.223 | -0.115 | 0.026 |
| PC 32:2 | -0.229 | 0.208 | 0.008 | 0.046 | 0.022 | -0.028 | 0.189 | 0.021 | -0.058 | 0.106 | 0.059 |
| PC 32:3 | 0.183 | 0.311 | -0.358 | -0.316 | 0.132 | 0.172 | 0.162 | 0.336 | -0.492 | -0.110 | -0.325 |
| PC 34:1 | 0.148 | 0.459 | -0.142 | 0.002 | 0.343 | -0.221 | 0.131 | 0.146 | -0.328 | 0.139 | -0.371 |
| PC 34:2 | 0.141 | 0.430 | -0.010 | -0.066 | 0.304 | -0.087 | 0.214 | 0.117 | -0.375 | 0.147 | -0.281 |
| PC 34:3 | 0.094 | 0.305 | -0.244 | -0.005 | 0.246 | -0.095 | 0.146 | 0.109 | -0.259 | 0.184 | -0.269 |
| PC 34:4 | -0.018 | 0.200 | -0.385 | -0.272 | 0.297 | 0.144 | 0.196 | 0.123 | -0.279 | -0.014 | -0.133 |
| PC 36:0 | 0.010 | 0.406 | 0.189 | -0.012 | 0.083 | 0.118 | -0.070 | 0.098 | -0.211 | -0.090 | 0.073 |
| PC 36:1 | 0.208 | 0.435 | -0.026 | -0.065 | 0.408 | -0.151 | 0.136 | 0.158 | -0.367 | 0.131 | -0.337 |
| PC 36:2 | 0.137 | 0.343 | -0.054 | -0.063 | 0.407 | -0.163 | 0.259 | 0.107 | -0.356 | 0.112 | -0.310 |
| PC 36:3 | 0.069 | 0.535 | -0.068 | -0.102 | 0.245 | -0.027 | 0.169 | -0.051 | -0.141 | 0.159 | -0.217 |
| PC 36:4 | 0.011 | 0.356 | -0.136 | -0.145 | 0.209 | 0.087 | 0.269 | 0.147 | -0.354 | 0.087 | -0.278 |
| PC 36:5 | 0.117 | 0.355 | -0.124 | -0.176 | 0.244 | 0.070 | 0.136 | 0.427 | -0.560 | -0.087 | -0.215 |
| PC 36:6 | 0.206 | 0.451 | 0.087 | -0.113 | 0.331 | -0.103 | 0.264 | 0.207 | -0.431 | -0.052 | -0.171 |
| PC 38:0 | 0.252 | 0.341 | -0.048 | -0.171 | 0.409 | -0.042 | 0.066 | 0.087 | -0.310 | 0.264 | -0.383 |
| PC 38:3 | 0.174 | 0.376 | 0.103 | -0.144 | 0.403 | -0.092 | 0.097 | -0.025 | -0.167 | 0.189 | -0.244 |
| PC 38:4 | 0.050 | 0.322 | -0.035 | -0.065 | 0.389 | -0.092 | 0.243 | 0.063 | -0.325 | 0.212 | -0.357 |
| PC 38:5 | 0.104 | 0.301 | -0.320 | -0.020 | 0.237 | -0.116 | 0.360 | 0.246 | -0.486 | -0.002 | -0.225 |
| PC 38:6 | 0.132 | 0.538 | 0.103 | -0.063 | 0.356 | -0.083 | 0.196 | 0.087 | -0.289 | -0.030 | -0.298 |
| PC 40:1 | -0.125 | -0.111 | -0.202 | -0.094 | 0.244 | 0.098 | 0.053 | 0.272 | -0.352 | -0.055 | -0.017 |
| PC 40:2 | 0.073 | -0.017 | 0.026 | 0.036 | 0.275 | -0.101 | 0.026 | 0.437 | -0.447 | -0.157 | -0.354 |
| PC 40:3 | -0.021 | 0.514 | -0.170 | 0.008 | 0.255 | -0.047 | 0.235 | 0.048 | -0.301 | 0.110 | -0.112 |
| PC 40:4 | 0.128 | 0.466 | -0.018 | -0.039 | 0.240 | -0.013 | 0.290 | -0.042 | -0.243 | 0.198 | -0.157 |
| PC 40:5 | 0.096 | 0.132 | -0.019 | -0.170 | 0.403 | -0.043 | 0.270 | 0.076 | -0.286 | 0.101 | -0.300 |
| PC 40:6 | 0.143 | 0.373 | 0.041 | -0.151 | 0.455 | -0.076 | 0.130 | 0.038 | -0.226 | 0.045 | -0.171 |
| PC 42:0 | -0.125 | -0.063 | -0.147 | -0.460 | 0.579 | 0.237 | 0.153 | -0.029 | -0.009 | 0.004 | -0.182 |
| PC 42:1 | -0.058 | 0.701 | 0.136 | 0.082 | 0.018 | -0.002 | 0.153 | 0.394 | -0.540 | -0.197 | -0.229 |
| PC 42:2 | 0.224 | 0.226 | -0.238 | -0.249 | 0.144 | 0.181 | 0.168 | 0.010 | -0.078 | 0.022 | 0.107 |
| PC 42:4 | 0.012 | 0.653 | 0.097 | -0.017 | 0.159 | 0.059 | 0.337 | -0.026 | -0.260 | 0.084 | -0.122 |
| PC 42:5 | 0.034 | 0.639 | 0.265 | 0.120 | 0.175 | -0.101 | 0.323 | 0.265 | -0.513 | -0.136 | -0.148 |
| PC 42:6 | -0.059 | 0.132 | -0.487 | 0.029 | 0.073 | -0.140 | 0.496 | 0.195 | -0.359 | -0.156 | 0.055 |
| GPCe 30:0 | 0.321 | 0.200 | -0.178 | -0.123 | 0.130 | -0.021 | 0.343 | 0.446 | -0.670 | -0.238 | -0.037 |
| GPCe 30:1 | 0.114 | 0.194 | 0.311 | 0.266 | -0.281 | -0.121 | 0.141 | 0.450 | -0.582 | -0.213 | 0.209 |
| GPCe 30:2 | -0.040 | 0.478 | -0.185 | 0.093 | 0.028 | -0.071 | 0.294 | 0.048 | -0.224 | -0.081 | 0.291 |
| GPCe 32:1 | 0.051 | 0.408 | -0.016 | -0.108 | 0.309 | -0.066 | 0.108 | -0.109 | -0.053 | 0.197 | -0.281 |
| GPCe 32:2 | 0.051 | 0.414 | 0.056 | -0.099 | 0.382 | -0.047 | 0.354 | -0.026 | -0.263 | 0.200 | -0.175 |
| GPCe 34:0 | 0.004 | 0.221 | -0.244 | -0.019 | 0.446 | -0.274 | 0.246 | -0.050 | -0.087 | 0.078 | -0.346 |
| GPCe 34:1 | 0.067 | 0.286 | -0.111 | -0.068 | 0.445 | -0.208 | 0.077 | 0.058 | -0.254 | 0.229 | -0.350 |
| GPCe 34:2 | 0.107 | 0.373 | -0.118 | 0.002 | 0.295 | -0.192 | 0.069 | -0.011 | -0.173 | 0.245 | -0.337 |
| GPCe 34:3 | 0.074 | 0.257 | -0.001 | -0.181 | 0.293 | 0.051 | 0.258 | -0.089 | -0.166 | 0.372 | -0.440 |
| GPCe 36:0 | 0.283 | 0.427 | -0.127 | -0.137 | 0.265 | -0.053 | 0.140 | 0.433 | -0.635 | -0.043 | -0.385 |
| GPCe 36:1 | 0.182 | 0.394 | -0.230 | -0.080 | 0.299 | -0.090 | 0.210 | 0.307 | -0.478 | -0.002 | -0.336 |
| GPCe 36:2 | 0.197 | 0.511 | -0.148 | -0.011 | 0.124 | -0.050 | 0.127 | 0.179 | -0.369 | 0.116 | -0.229 |
| GPCe 36:3 | 0.038 | 0.365 | -0.100 | -0.062 | 0.314 | -0.110 | 0.203 | 0.044 | -0.224 | 0.172 | -0.405 |
| GPCe 36:4 | 0.056 | 0.192 | -0.193 | 0.015 | 0.272 | -0.135 | 0.077 | 0.087 | -0.186 | 0.182 | -0.483 |
| GPCe 36:5 | -0.071 | 0.328 | -0.220 | 0.025 | 0.274 | -0.131 | 0.287 | -0.018 | -0.232 | 0.272 | -0.404 |
| GPCe 38:0 | 0.181 | 0.364 | -0.275 | -0.148 | 0.339 | -0.031 | 0.168 | 0.242 | -0.458 | 0.067 | -0.234 |
| GPCe 38:1 | -0.214 | -0.027 | -0.164 | -0.105 | 0.234 | 0.047 | -0.009 | 0.169 | -0.074 | -0.054 | 0.003 |
| GPCe 38:2 | -0.079 | 0.132 | -0.303 | 0.232 | 0.024 | -0.255 | 0.324 | 0.158 | -0.278 | 0.015 | -0.171 |
| GPCe 38:3 | 0.025 | 0.308 | -0.077 | -0.045 | 0.257 | -0.040 | 0.165 | 0.039 | -0.242 | 0.291 | -0.162 |
| GPCe 38:4 | 0.109 | 0.559 | -0.105 | 0.036 | 0.100 | -0.050 | 0.193 | 0.166 | -0.341 | 0.070 | -0.252 |
| GPCe 38:5 | 0.067 | 0.454 | -0.312 | 0.120 | 0.120 | -0.158 | 0.144 | 0.006 | -0.227 | 0.295 | -0.326 |
| GPCe 38:6 | 0.149 | 0.314 | 0.177 | -0.056 | 0.397 | -0.135 | 0.029 | -0.068 | -0.103 | 0.197 | -0.302 |
| GPCe 40:1 | 0.133 | -0.255 | 0.017 | -0.083 | 0.064 | 0.034 | -0.093 | 0.266 | 0.018 | -0.422 | 0.130 |
| GPCe 40:2 | 0.282 | 0.383 | -0.183 | -0.142 | 0.274 | -0.079 | 0.140 | 0.293 | -0.397 | -0.125 | -0.184 |
| GPCe 40:3 | -0.135 | 0.400 | -0.074 | -0.017 | 0.300 | -0.050 | 0.366 | 0.087 | -0.273 | 0.015 | -0.123 |
| GPCe 40:4 | 0.146 | 0.084 | -0.298 | -0.191 | 0.324 | 0.050 | 0.273 | 0.201 | -0.352 | -0.104 | -0.015 |
| GPCe 40:5 | -0.006 | 0.608 | -0.164 | 0.103 | 0.067 | -0.076 | 0.180 | 0.229 | -0.477 | 0.106 | -0.191 |
| GPCe 40:6 | 0.064 | 0.356 | 0.096 | -0.059 | 0.368 | -0.160 | -0.021 | 0.005 | -0.111 | 0.061 | -0.171 |
| GPCe 42:0 | 0.058 | -0.002 | -0.143 | 0.073 | 0.148 | -0.101 | 0.522 | 0.040 | -0.289 | -0.027 | 0.228 |
| GPCe 42:1 | 0.162 | -0.027 | -0.520 | -0.059 | 0.201 | -0.034 | 0.242 | 0.137 | -0.369 | 0.180 | -0.185 |
| GPCe 42:2 | 0.049 | 0.223 | -0.050 | -0.164 | 0.372 | -0.006 | -0.027 | 0.187 | -0.178 | 0.052 | -0.335 |
| GPCe 42:3 | 0.148 | 0.329 | -0.250 | 0.196 | 0.059 | -0.184 | 0.220 | 0.362 | -0.558 | -0.117 | -0.106 |
| GPCe 42:4 | -0.084 | 0.544 | -0.123 | 0.257 | -0.300 | -0.057 | -0.025 | 0.264 | -0.321 | 0.045 | 0.089 |
| GPCe 42:5 | -0.025 | 0.362 | -0.133 | 0.341 | 0.104 | -0.432 | 0.470 | 0.108 | -0.358 | -0.061 | 0.077 |
| GPCe 44:3 | 0.182 | 0.251 | -0.246 | -0.144 | 0.470 | -0.137 | 0.127 | 0.239 | -0.360 | 0.043 | -0.385 |
| GPCe 44:4 | -0.339 | 0.247 | 0.025 | -0.116 | 0.078 | 0.221 | 0.019 | 0.180 | -0.144 | -0.241 | 0.027 |
| GPCe 44:5 | -0.028 | -0.009 | -0.360 | 0.020 | -0.114 | 0.045 | 0.118 | 0.344 | -0.338 | -0.304 | 0.091 |
| GPCe 44:6 | 0.063 | 0.129 | -0.530 | -0.099 | 0.113 | 0.098 | 0.381 | -0.033 | -0.271 | 0.280 | -0.027 |
| SM (OH) C14:1 | 0.105 | 0.374 | -0.188 | -0.014 | 0.371 | -0.221 | 0.104 | 0.194 | -0.349 | 0.098 | -0.386 |
| SM (OH) C16:1 | 0.176 | 0.362 | -0.022 | -0.036 | 0.423 | -0.234 | 0.064 | 0.236 | -0.403 | 0.070 | -0.372 |
| SM (OH) C22:1 | 0.184 | 0.338 | -0.139 | -0.011 | 0.266 | -0.207 | 0.124 | 0.255 | -0.431 | 0.098 | -0.346 |
| SM (OH) C22:2 | 0.166 | 0.447 | -0.067 | 0.014 | 0.338 | -0.211 | 0.114 | 0.226 | -0.427 | 0.087 | -0.372 |
| SM (OH) C24:1 | 0.181 | 0.435 | -0.115 | 0.008 | 0.264 | -0.205 | 0.127 | 0.260 | -0.462 | 0.085 | -0.326 |
| SM C16:0 | 0.208 | 0.344 | -0.017 | -0.099 | 0.462 | -0.192 | 0.059 | 0.182 | -0.325 | 0.033 | -0.273 |
| SM C16:1 | 0.164 | 0.386 | 0.056 | -0.128 | 0.426 | -0.122 | 0.073 | 0.220 | -0.386 | -0.008 | -0.351 |
| SM C18:0 | 0.255 | 0.394 | -0.061 | -0.029 | 0.396 | -0.216 | 0.118 | 0.161 | -0.375 | 0.140 | -0.307 |
| SM C18:1 | 0.162 | 0.341 | 0.139 | -0.114 | 0.474 | -0.137 | 0.069 | 0.085 | -0.268 | 0.090 | -0.378 |
| SM C20:2 | 0.244 | 0.547 | 0.021 | -0.140 | 0.204 | 0.006 | 0.264 | 0.253 | -0.471 | -0.028 | -0.277 |
| SM C22:3 | -0.173 | 0.132 | 0.127 | 0.199 | 0.167 | -0.233 | 0.532 | -0.439 | 0.212 | 0.141 | -0.078 |
| SM C24:0 | 0.239 | 0.441 | -0.091 | -0.026 | 0.258 | -0.155 | 0.092 | 0.177 | -0.372 | 0.192 | -0.306 |
| SM C24:1 | 0.282 | 0.442 | -0.018 | -0.052 | 0.270 | -0.128 | 0.126 | 0.200 | -0.391 | 0.082 | -0.231 |
| SM C26:0 | 0.011 | 0.344 | -0.181 | -0.068 | 0.205 | -0.054 | 0.104 | 0.032 | -0.213 | 0.292 | -0.285 |
| SM C26:1 | 0.053 | 0.568 | -0.052 | -0.045 | 0.143 | -0.014 | 0.136 | -0.016 | -0.168 | 0.115 | -0.133 |
| H1 | -0.083 | 0.480 | 0.439 | 0.279 | -0.501 | 0.054 | -0.109 | 0.295 | -0.273 | -0.151 | 0.220 |
