# Supplementary material for: Metabolomic profiling reveals correlations between spermiogram parameters and the metabolites present in human spermatozoa and seminal plasma
Source: PLoS One. 2019 Feb 20;14(2):e0211679. doi: 10.1371/journal.pone.0211679 (PMC6382115; doi:10.1371/journal.pone.0211679)
Supplement: S8 Table — Data are Spearman correlation rank coefficients. Significances are highlighted in bolt. Abbreviations: C0—DL-carnitine, C10—decanoyl-L-carnitine, C10:1—decenoyl-L-carnitine, C10:2—decadienyl-L-carnitine, C12—dodecanoyl-L-carnitine, C12:1—dodecenoyl-L-carnitine, C12-DC—dodecanedioyl-L-carnitine, C14—tetradecanoyl-L-carnitine, C14:1—tetradecenoyl-L-carnitine, C14:1-OH—hydroxytetradecenoyl-L-carnitine, C14:2—tetradecadienyl-L-carnitine, C14:2-OH—hydroxytetradecadienyl-L-carnitine, C16—hexadecanoyl-L-carnitine, C16:1—hexadecenoyl-L-carnitine, C16:1-OH—hydroxyhexadecenoyl-L-carnitine, C16:2—hexadecadienyl-L-carnitine, C16:2-OH—hydroxyhexadecadienyl-L-carnitine, C16-OH—hydroxyhexadecanoyl-L-carnitine, C18—octadecanoyl-L-carnitine, C18:1—octadecenoyl-L-carnitine, C18:1-OH—hydroxyoctadecenoyl-L-carnitine, C18:2—octadecadienyl-L-carnitine, C2—acetyl-L-carnitine, C3—propionyl-L-carnitine, C3:1—propenyl-L-carnitine, C3-DC/C4-OH—malonyl-L-carnitine/hydroxybutyryl-L-carnitine, C3-DC-M/C5-OH—methylmalonyl-L-carnitine/hydroxyvaleryl-L-carnitine, C3-OH—hydroxypropionyl-L-carnitine, C4—butyryl-L-carnitine, C4:1—butenyl-L-carnitine, C4:1-DC/C6—fumaryl-L-carnitine/hexanoyl-L-carnitine, C5—valeryl-L-carnitine, C5:1—tiglyl-L-carnitine, C5:1-DC—glutaconyl-L-carnitine, C5-DC/C6-OH—glutaryl-L-carnitine/hydroxyhexanoyl-L-carnitine, C5-M-DC—methylglutaryl-L-carnitine, C6:1—hexenoyl-L-carnitine, C7-DC—pimelyl-L-carnitine, C8—octanoyl-L-carnitine, C8:1—octenoyl-L-carnitine, C9—nonayl-L-carnitine. (DOCX) [file pone.0211679.s009.docx]

| sperm  SP | C0 | C10 | C10:1 | C10:2 | C12 | C12-DC | C12:1 | C14 | C14:1 | C14:1-OH | C14:2 | C14:2-OH | C16 | C16-OH | C16:1 | C16:1-OH | C16:2 | C16:2-OH | C18 | C18:1 | C18:1-OH | C18:2 | **C2** | C3 | C3-DC (C4-OH) | C3-OH | C3:1 | C4 | C4:1 | C5 | C5-DC (C6-OH) | C5-M-DC | C5-OH (C3-DC-M) | C5:1 | C5:1-DC | C6 (C4:1-DC) | C6:1 | C7-DC | C8 | C9 |
| --- | --- | --- | --- | --- | --- | --- | --- | --- | --- | --- | --- | --- | --- | --- | --- | --- | --- | --- | --- | --- | --- | --- | --- | --- | --- | --- | --- | --- | --- | --- | --- | --- | --- | --- | --- | --- | --- | --- | --- | --- |
| C0 | -0.035 | -0.286 | -0.293 | -0.271 | -0.289 | -0.278 | -0.281 | -0.095 | -0.119 | -0.181 | -0.135 | -0.227 | -0.099 | -0.292 | -0.277 | -0.290 | -0.248 | -0.252 | -0.313 | -0.205 | -0.306 | -0.249 | 0.108 | 0.026 | -0.041 | -0.274 | -0.245 | -0.005 | -0.137 | 0.009 | -0.241 | -0.381 | -0.044 | -0.194 | -0.316 | -0.209 | -0.202 | -0.279 | -0.295 | -0.277 |
| C10 | -0.223 | -0.025 | 0.048 | -0.045 | -0.056 | -0.033 | -0.036 | -0.133 | -0.109 | -0.013 | -0.184 | -0.100 | -0.187 | -0.020 | -0.102 | 0.005 | -0.077 | -0.063 | -0.256 | -0.267 | -0.069 | -0.066 | -0.098 | -0.162 | -0.200 | -0.004 | -0.014 | -0.292 | -0.103 | -0.227 | -0.145 | -0.032 | -0.275 | -0.215 | -0.068 | -0.188 | -0.042 | -0.030 | -0.043 | 0.005 |
| C10:1 | **-0.573** | 0.035 | 0.005 | 0.006 | -0.028 | 0.024 | -0.004 | 0.075 | 0.017 | 0.081 | 0.037 | 0.043 | 0.001 | -0.043 | 0.001 | -0.065 | 0.044 | 0.002 | -0.047 | -0.021 | -0.082 | 0.019 | **-0.499** | -0.417 | -0.440 | 0.039 | 0.121 | -0.499 | -0.082 | **-0.450** | -0.134 | 0.017 | -0.433 | -0.164 | -0.055 | -0.134 | 0.060 | 0.017 | 0.027 | 0.054 |
| C10:2 | 0.194 | 0.066 | 0.105 | 0.051 | 0.041 | 0.053 | 0.056 | 0.005 | 0.023 | 0.061 | -0.041 | -0.021 | 0.076 | 0.140 | -0.068 | 0.069 | -0.046 | 0.025 | -0.105 | -0.026 | 0.007 | -0.017 | 0.185 | 0.259 | 0.077 | 0.110 | 0.093 | 0.245 | 0.211 | 0.197 | 0.147 | 0.026 | 0.150 | 0.142 | 0.104 | 0.117 | 0.183 | 0.038 | 0.035 | 0.130 |
| C12 | -0.069 | 0.005 | -0.023 | 0.000 | 0.047 | 0.009 | -0.028 | 0.125 | 0.062 | 0.083 | -0.006 | -0.029 | 0.107 | 0.019 | -0.025 | -0.069 | 0.029 | 0.011 | -0.105 | 0.058 | -0.078 | -0.038 | 0.138 | 0.128 | -0.089 | -0.004 | 0.015 | -0.089 | -0.012 | 0.125 | 0.015 | -0.124 | 0.015 | -0.005 | 0.043 | -0.003 | -0.003 | -0.034 | -0.008 | 0.014 |
| C12-DC | -0.426 | -0.257 | -0.234 | -0.223 | -0.293 | -0.267 | -0.269 | -0.222 | -0.260 | -0.186 | -0.159 | -0.212 | -0.188 | -0.298 | -0.284 | -0.304 | -0.175 | -0.268 | -0.360 | -0.306 | -0.327 | -0.287 | -0.251 | -0.361 | -0.234 | -0.246 | -0.157 | -0.286 | -0.263 | -0.308 | -0.319 | -0.258 | -0.384 | -0.394 | -0.292 | -0.313 | -0.187 | -0.271 | -0.272 | -0.180 |
| C12:1 | **-0.508** | -0.110 | -0.146 | -0.104 | -0.143 | -0.117 | -0.143 | 0.015 | -0.077 | -0.036 | -0.041 | -0.066 | -0.005 | -0.213 | -0.105 | -0.216 | -0.039 | -0.114 | -0.156 | -0.072 | -0.221 | -0.105 | **-0.547** | -0.547 | -0.397 | -0.117 | -0.025 | -0.417 | -0.162 | **-0.448** | -0.157 | -0.127 | -0.442 | -0.215 | -0.114 | -0.194 | -0.090 | -0.104 | -0.106 | -0.084 |
| C14 | -0.066 | 0.253 | 0.272 | 0.223 | 0.223 | 0.244 | 0.275 | 0.237 | 0.286 | 0.269 | 0.256 | 0.276 | 0.184 | 0.214 | 0.261 | 0.270 | 0.242 | 0.250 | 0.255 | 0.226 | 0.238 | 0.258 | -0.080 | 0.180 | -0.182 | 0.245 | 0.286 | 0.005 | 0.191 | 0.066 | 0.084 | 0.250 | -0.039 | 0.088 | 0.145 | 0.176 | 0.302 | 0.219 | 0.229 | 0.268 |
| C14:1 | -0.045 | -0.038 | 0.014 | -0.062 | -0.044 | -0.053 | -0.024 | -0.061 | -0.116 | -0.072 | -0.111 | -0.080 | 0.035 | 0.031 | -0.105 | 0.004 | -0.119 | -0.078 | 0.011 | -0.001 | -0.051 | -0.047 | 0.002 | 0.173 | -0.142 | 0.011 | -0.002 | -0.023 | 0.017 | -0.059 | 0.033 | 0.041 | -0.123 | -0.027 | -0.008 | 0.011 | 0.089 | -0.037 | -0.062 | 0.048 |
| C14:1-OH | 0.126 | 0.096 | 0.128 | 0.068 | 0.078 | 0.079 | 0.092 | -0.050 | -0.045 | 0.038 | -0.003 | 0.022 | 0.056 | 0.167 | -0.015 | 0.127 | -0.009 | 0.051 | 0.082 | 0.067 | 0.074 | 0.040 | 0.245 | 0.289 | 0.145 | 0.100 | 0.095 | 0.120 | 0.114 | 0.143 | 0.105 | 0.100 | 0.145 | 0.124 | 0.061 | 0.110 | 0.163 | 0.041 | 0.063 | 0.153 |
| C14:2 | 0.185 | -0.019 | 0.037 | -0.026 | 0.025 | -0.031 | -0.026 | -0.028 | -0.101 | -0.058 | -0.143 | -0.115 | 0.102 | 0.148 | -0.130 | 0.035 | -0.132 | -0.065 | 0.021 | 0.058 | -0.025 | -0.076 | 0.277 | 0.331 | 0.126 | 0.021 | -0.015 | 0.191 | 0.071 | 0.177 | 0.122 | -0.023 | 0.147 | 0.105 | 0.059 | 0.120 | 0.092 | -0.063 | -0.041 | 0.058 |
| C14:2-OH | 0.373 | -0.062 | -0.007 | -0.072 | -0.046 | -0.077 | -0.053 | -0.180 | -0.188 | -0.113 | -0.151 | -0.129 | 0.004 | 0.072 | -0.163 | 0.016 | -0.161 | -0.090 | -0.012 | -0.024 | -0.047 | -0.094 | **0.516** | 0.442 | 0.378 | -0.060 | -0.087 | 0.326 | 0.026 | 0.332 | 0.062 | -0.057 | 0.311 | 0.093 | -0.042 | 0.068 | 0.020 | -0.108 | -0.097 | 0.011 |
| C16 | 0.389 | -0.091 | -0.030 | -0.146 | -0.019 | -0.095 | -0.050 | 0.109 | 0.000 | -0.064 | -0.117 | -0.106 | 0.271 | 0.137 | -0.096 | 0.024 | -0.135 | -0.084 | 0.277 | 0.267 | -0.040 | -0.029 | **0.492** | **0.528** | 0.359 | -0.063 | -0.056 | 0.266 | -0.030 | 0.355 | 0.023 | -0.087 | 0.378 | 0.084 | -0.086 | 0.111 | 0.065 | -0.136 | -0.141 | -0.032 |
| C16-OH | 0.269 | -0.132 | -0.124 | -0.132 | -0.172 | -0.134 | -0.126 | -0.108 | -0.112 | -0.062 | -0.130 | -0.141 | -0.054 | -0.097 | -0.201 | -0.138 | -0.172 | -0.129 | -0.216 | -0.109 | -0.178 | -0.115 | 0.435 | 0.251 | 0.256 | -0.128 | -0.140 | 0.071 | -0.001 | 0.241 | -0.042 | -0.198 | 0.188 | -0.001 | -0.169 | -0.074 | -0.032 | -0.167 | -0.166 | -0.095 |
| C16:1 | -0.071 | -0.245 | -0.226 | -0.301 | -0.184 | -0.242 | -0.248 | -0.049 | -0.175 | -0.196 | -0.321 | -0.298 | 0.008 | -0.177 | -0.264 | -0.236 | -0.275 | -0.241 | -0.223 | -0.107 | -0.300 | -0.222 | 0.044 | 0.146 | -0.259 | -0.202 | -0.215 | -0.138 | -0.200 | -0.059 | -0.110 | -0.270 | -0.151 | -0.169 | -0.134 | -0.204 | -0.200 | -0.197 | -0.266 | -0.207 |
| C16:1-OH | -0.008 | -0.146 | -0.140 | -0.161 | -0.138 | -0.154 | -0.172 | -0.040 | -0.118 | -0.067 | -0.154 | -0.182 | 0.047 | -0.115 | -0.226 | -0.186 | -0.169 | -0.152 | -0.241 | -0.108 | -0.242 | -0.172 | 0.111 | 0.075 | -0.012 | -0.142 | -0.102 | 0.063 | -0.049 | 0.081 | -0.063 | -0.246 | 0.034 | -0.069 | -0.106 | -0.099 | -0.069 | -0.173 | -0.177 | -0.086 |
| C16:2 | 0.253 | -0.421 | -0.371 | **-0.462** | -0.290 | -0.409 | -0.395 | -0.130 | -0.283 | -0.343 | **-0.460** | **-0.466** | 0.018 | -0.210 | -0.413 | -0.321 | **-0.454** | -0.385 | -0.162 | -0.092 | -0.370 | -0.336 | **0.517** | 0.388 | 0.193 | -0.397 | -0.450 | 0.147 | -0.280 | 0.284 | -0.166 | **-0.491** | 0.259 | -0.132 | -0.299 | -0.172 | -0.337 | -0.427 | **-0.456** | -0.384 |
| C16:2-OH | 0.358 | -0.304 | -0.275 | -0.319 | -0.229 | -0.307 | -0.284 | -0.196 | -0.282 | -0.309 | -0.328 | -0.366 | -0.007 | -0.178 | -0.352 | -0.237 | -0.371 | -0.297 | -0.121 | -0.054 | -0.263 | -0.283 | **0.538** | **0.502** | 0.213 | -0.299 | -0.344 | 0.283 | -0.096 | 0.379 | -0.042 | -0.338 | 0.328 | -0.005 | -0.198 | -0.078 | -0.208 | -0.343 | -0.333 | -0.259 |
| C18 | 0.242 | -0.114 | -0.077 | -0.173 | -0.028 | -0.105 | -0.087 | 0.188 | 0.033 | -0.031 | -0.118 | -0.110 | 0.275 | 0.097 | -0.087 | -0.036 | -0.119 | -0.089 | 0.169 | 0.209 | -0.100 | -0.028 | 0.305 | 0.317 | 0.235 | -0.070 | -0.073 | 0.188 | -0.098 | 0.217 | 0.007 | -0.137 | 0.257 | 0.023 | -0.083 | 0.039 | 0.003 | -0.106 | -0.152 | -0.071 |
| C18:1 | 0.156 | -0.105 | -0.066 | -0.149 | -0.063 | -0.105 | -0.090 | 0.119 | 0.002 | -0.026 | -0.148 | -0.102 | 0.187 | 0.094 | -0.103 | -0.066 | -0.125 | -0.102 | 0.120 | 0.137 | -0.121 | -0.051 | 0.244 | 0.260 | 0.159 | -0.066 | -0.045 | 0.068 | -0.110 | 0.168 | -0.047 | -0.115 | 0.154 | -0.026 | -0.090 | 0.026 | 0.031 | -0.124 | -0.141 | -0.051 |
| C18:1-OH | 0.298 | -0.041 | -0.011 | -0.027 | -0.046 | -0.059 | -0.056 | -0.155 | -0.145 | -0.055 | -0.143 | -0.090 | -0.023 | 0.055 | -0.128 | -0.036 | -0.116 | -0.067 | -0.009 | -0.029 | -0.053 | -0.070 | **0.531** | 0.414 | 0.322 | -0.072 | -0.062 | 0.156 | 0.020 | 0.394 | 0.005 | -0.076 | 0.282 | 0.078 | -0.031 | 0.081 | 0.020 | -0.130 | -0.063 | 0.011 |
| C18:2 | -0.179 | 0.068 | 0.057 | 0.014 | 0.099 | 0.066 | 0.038 | 0.188 | 0.123 | 0.090 | -0.054 | 0.011 | 0.096 | 0.152 | 0.054 | 0.035 | 0.031 | 0.045 | 0.096 | 0.122 | 0.004 | 0.033 | -0.116 | 0.086 | -0.296 | 0.077 | 0.101 | -0.168 | -0.082 | -0.018 | -0.054 | 0.002 | -0.077 | -0.055 | 0.056 | -0.012 | 0.051 | 0.032 | 0.062 | 0.042 |
| C2 | -0.135 | -0.355 | -0.312 | -0.346 | -0.316 | -0.358 | -0.353 | -0.249 | -0.366 | -0.314 | -0.328 | -0.325 | -0.128 | -0.238 | -0.364 | -0.288 | -0.385 | -0.352 | -0.165 | -0.196 | -0.329 | -0.281 | 0.116 | -0.065 | 0.065 | -0.346 | -0.347 | -0.189 | -0.304 | -0.211 | -0.240 | -0.345 | -0.170 | -0.209 | -0.334 | -0.173 | -0.264 | -0.350 | -0.363 | -0.289 |
| C3 | 0.101 | -0.386 | -0.405 | -0.411 | -0.281 | -0.389 | -0.404 | -0.043 | -0.259 | -0.327 | -0.352 | -0.387 | 0.078 | -0.238 | -0.379 | -0.375 | -0.407 | -0.369 | -0.053 | 0.037 | -0.415 | -0.319 | 0.337 | **0.460** | -0.088 | -0.406 | -0.365 | 0.087 | -0.319 | 0.269 | -0.234 | **-0.488** | 0.134 | -0.165 | -0.318 | -0.175 | -0.302 | -0.432 | -0.393 | -0.363 |
| C3-DC (C4-OH) | 0.024 | -0.383 | -0.355 | -0.353 | -0.302 | -0.374 | -0.375 | -0.153 | -0.294 | -0.318 | -0.287 | -0.342 | -0.049 | -0.274 | -0.358 | -0.300 | -0.374 | -0.351 | -0.159 | -0.122 | -0.328 | -0.290 | 0.179 | -0.059 | 0.171 | -0.383 | -0.400 | -0.026 | -0.263 | -0.086 | -0.196 | -0.433 | -0.009 | -0.147 | -0.336 | -0.153 | -0.316 | -0.380 | -0.378 | -0.354 |
| C3-OH | 0.275 | 0.123 | 0.165 | 0.086 | 0.172 | 0.139 | 0.161 | 0.145 | 0.179 | 0.151 | 0.035 | 0.068 | 0.165 | 0.291 | 0.111 | 0.204 | 0.065 | 0.138 | 0.183 | 0.172 | 0.200 | 0.134 | 0.333 | 0.159 | 0.419 | 0.160 | 0.063 | 0.221 | 0.179 | 0.244 | 0.216 | 0.092 | 0.390 | 0.264 | 0.142 | 0.246 | 0.174 | 0.099 | 0.084 | 0.123 |
| C3:1 | 0.047 | -0.239 | -0.223 | -0.205 | -0.132 | -0.218 | -0.228 | -0.102 | -0.188 | -0.181 | -0.201 | -0.242 | -0.051 | -0.180 | -0.212 | -0.196 | -0.212 | -0.199 | -0.246 | -0.123 | -0.196 | -0.195 | 0.311 | 0.146 | 0.046 | -0.221 | -0.308 | -0.024 | -0.138 | 0.069 | -0.016 | -0.289 | -0.029 | -0.069 | -0.106 | -0.072 | -0.241 | -0.200 | -0.234 | -0.211 |
| C4 | -0.147 | -0.375 | -0.350 | -0.350 | -0.358 | -0.385 | -0.375 | -0.193 | -0.328 | -0.342 | -0.294 | -0.326 | -0.117 | -0.371 | -0.367 | -0.333 | -0.385 | -0.373 | -0.177 | -0.176 | -0.373 | -0.283 | -0.053 | 0.006 | -0.253 | -0.382 | -0.341 | -0.165 | -0.270 | -0.155 | -0.297 | -0.369 | -0.256 | -0.282 | -0.373 | -0.258 | -0.276 | -0.380 | -0.363 | -0.330 |
| C4:1 | -0.056 | -0.199 | -0.198 | -0.171 | -0.195 | -0.212 | -0.245 | -0.136 | -0.259 | -0.197 | -0.254 | -0.253 | -0.043 | -0.142 | -0.303 | -0.238 | -0.277 | -0.240 | -0.203 | -0.124 | -0.266 | -0.219 | 0.036 | -0.018 | -0.070 | -0.187 | -0.180 | -0.080 | -0.072 | -0.038 | -0.056 | -0.243 | -0.079 | -0.046 | -0.129 | -0.089 | -0.110 | -0.237 | -0.190 | -0.146 |
| C5 | 0.057 | -0.361 | -0.376 | -0.353 | -0.279 | -0.363 | -0.378 | -0.008 | -0.195 | -0.257 | -0.267 | -0.316 | 0.038 | -0.329 | -0.323 | -0.358 | -0.333 | -0.324 | -0.173 | -0.053 | -0.409 | -0.272 | 0.191 | 0.334 | -0.223 | -0.400 | -0.341 | 0.066 | -0.291 | 0.197 | -0.267 | **-0.486** | -0.012 | -0.237 | -0.317 | -0.225 | -0.316 | -0.381 | -0.352 | -0.349 |
| C5-DC (C6-OH) | -0.105 | -0.377 | -0.363 | -0.335 | -0.291 | -0.370 | -0.402 | -0.151 | -0.332 | -0.293 | -0.316 | -0.353 | -0.090 | -0.328 | -0.370 | -0.354 | -0.351 | -0.353 | -0.323 | -0.226 | -0.395 | -0.298 | 0.135 | -0.015 | -0.083 | -0.385 | -0.391 | -0.117 | -0.312 | -0.072 | -0.221 | **-0.457** | -0.175 | -0.246 | -0.299 | -0.242 | -0.362 | -0.354 | -0.358 | -0.335 |
| C5-M-DC | -0.126 | -0.240 | -0.233 | -0.217 | -0.170 | -0.231 | -0.266 | -0.063 | -0.181 | -0.175 | -0.252 | -0.242 | -0.060 | -0.141 | -0.238 | -0.231 | -0.266 | -0.230 | -0.175 | -0.151 | -0.235 | -0.189 | 0.054 | -0.053 | -0.067 | -0.234 | -0.265 | -0.087 | -0.185 | -0.051 | -0.134 | -0.317 | -0.078 | -0.106 | -0.165 | -0.087 | -0.219 | -0.238 | -0.221 | -0.231 |
| C5-OH (C3-DC-M) | -0.132 | **-0.540** | **-0.551** | **-0.544** | **-0.518** | **-0.545** | **-0.567** | -0.288 | -0.414 | **-0.451** | **-0.513** | **-0.533** | -0.221 | -0.422 | **-0.575** | **-0.577** | **-0.554** | **-0.540** | -0.429 | -0.319 | **-0.616** | **-0.526** | 0.161 | 0.119 | -0.204 | **-0.530** | **-0.471** | -0.156 | **-0.445** | 0.029 | **-0.455** | **-0.638** | -0.124 | -0.394 | **-0.508** | -0.401 | -0.421 | **-0.577** | **-0.551** | **-0.506** |
| C5:1 | 0.095 | -0.339 | -0.326 | -0.347 | -0.269 | -0.331 | -0.340 | 0.007 | -0.127 | -0.230 | -0.261 | -0.306 | 0.029 | -0.254 | -0.311 | -0.304 | -0.327 | -0.302 | -0.210 | -0.112 | -0.357 | -0.256 | 0.212 | 0.295 | -0.118 | -0.324 | -0.296 | 0.120 | -0.214 | 0.182 | -0.245 | **-0.446** | 0.039 | -0.203 | -0.303 | -0.201 | -0.255 | -0.323 | -0.340 | -0.326 |
| C5:1-DC | 0.009 | -0.356 | -0.348 | -0.335 | -0.289 | -0.358 | -0.388 | -0.226 | -0.360 | -0.285 | -0.373 | -0.375 | -0.134 | -0.275 | -0.399 | -0.369 | -0.366 | -0.350 | -0.394 | -0.258 | -0.426 | -0.353 | 0.316 | 0.230 | -0.060 | -0.370 | -0.366 | -0.020 | -0.316 | 0.113 | -0.211 | **-0.454** | -0.076 | -0.241 | -0.258 | -0.235 | -0.333 | -0.364 | -0.360 | -0.298 |
| C6 (C4:1-DC) | 0.146 | **-0.587** | **-0.547** | **-0.562** | **-0.470** | **-0.573** | **-0.550** | -0.303 | **-0.470** | **-0.515** | **-0.490** | **-0.529** | -0.195 | **-0.458** | **-0.505** | **-0.459** | -0.559 | **-0.530** | -0.240 | -0.230 | **-0.485** | -0.419 | **0.484** | 0.286 | 0.106 | **-0.603** | **-0.651** | -0.024 | **-0.482** | 0.140 | -0.372 | **-0.612** | 0.035 | -0.336 | **-0.539** | -0.336 | **-0.537** | **-0.580** | **-0.584** | **-0.566** |
| C6:1 | 0.113 | -0.409 | -0.352 | -0.402 | -0.303 | -0.403 | -0.400 | -0.197 | -0.348 | -0.337 | -0.371 | -0.400 | -0.051 | -0.278 | -0.396 | -0.303 | -0.409 | -0.375 | -0.209 | -0.183 | -0.358 | -0.312 | 0.358 | 0.138 | 0.205 | -0.416 | -0.434 | 0.057 | -0.307 | 0.054 | -0.218 | **-0.466** | 0.065 | -0.194 | -0.336 | -0.195 | -0.363 | -0.401 | -0.421 | -0.359 |
| C7-DC | -0.125 | -0.275 | -0.263 | -0.233 | -0.223 | -0.268 | -0.295 | -0.100 | -0.250 | -0.194 | -0.208 | -0.246 | -0.065 | -0.257 | -0.284 | -0.259 | -0.270 | -0.258 | -0.303 | -0.195 | -0.305 | -0.211 | 0.024 | -0.095 | -0.099 | -0.267 | -0.288 | -0.123 | -0.193 | -0.176 | -0.121 | -0.335 | -0.229 | -0.163 | -0.211 | -0.162 | -0.238 | -0.237 | -0.258 | -0.227 |
| C8 | -0.209 | -0.428 | -0.415 | -0.427 | -0.381 | -0.412 | -0.425 | -0.139 | -0.250 | -0.336 | -0.343 | -0.390 | -0.168 | -0.338 | -0.400 | -0.391 | -0.414 | -0.403 | -0.355 | -0.272 | -0.425 | -0.361 | -0.014 | -0.069 | -0.229 | -0.375 | -0.387 | -0.214 | -0.336 | -0.206 | -0.344 | **-0.484** | -0.252 | -0.333 | -0.408 | -0.324 | -0.346 | -0.381 | -0.421 | -0.420 |
| C9 | -0.205 | -0.340 | -0.347 | -0.310 | -0.287 | -0.333 | -0.372 | -0.142 | -0.323 | -0.248 | -0.324 | -0.346 | -0.123 | -0.334 | -0.358 | -0.367 | -0.324 | -0.329 | -0.370 | -0.230 | -0.402 | -0.276 | 0.029 | -0.158 | -0.184 | -0.349 | -0.367 | -0.278 | -0.300 | -0.141 | -0.199 | -0.427 | -0.232 | -0.251 | -0.281 | -0.293 | -0.335 | -0.337 | -0.329 | -0.309 |
