# Supplementary material for: Metabolomic profiling reveals correlations between spermiogram parameters and the metabolites present in human spermatozoa and seminal plasma
Source: PLoS One. 2019 Feb 20;14(2):e0211679. doi: 10.1371/journal.pone.0211679 (PMC6382115; doi:10.1371/journal.pone.0211679)
Supplement: S9 Table — Data are Spearman correlation rank coefficients. Significances are highlighted in bolt. (DOCX) [file pone.0211679.s010.docx]

| sperm  SP | LPC 14:0 | LPC 16:0 | LPC 16:1 | LPC 17:0 | LPC 18:0 | LPC 18:1 | LPC 18:2 | LPC 20:3 | LPC 20:4 |
| --- | --- | --- | --- | --- | --- | --- | --- | --- | --- |
| \| LPC 14:0 \| \| --- \| | 0.110 | 0.323 | 0.130 | 0.164 | 0.317 | 0.096 | 0.156 | 0.113 | 0.196 |
| LPC 16:0 | -0.125 | 0.039 | -0.065 | -0.005 | -0.015 | -0.129 | -0.131 | -0.093 | 0.007 |
| LPC 16:1 | 0.003 | -0.051 | -0.082 | -0.001 | -0.080 | -0.036 | -0.110 | -0.044 | -0.153 |
| LPC 17:0 | -0.017 | 0.063 | 0.004 | 0.054 | 0.008 | -0.015 | -0.036 | -0.006 | 0.045 |
| LPC 18:0 | -0.147 | 0.044 | -0.091 | 0.004 | -0.006 | -0.159 | -0.125 | -0.098 | 0.025 |
| LPC 18:1 | -0.126 | -0.039 | -0.107 | -0.041 | -0.081 | -0.143 | -0.165 | -0.109 | -0.079 |
| LPC 18:2 | 0.119 | 0.205 | 0.145 | 0.151 | 0.160 | 0.174 | 0.132 | 0.126 | 0.122 |
| LPC 20:3 | -0.098 | 0.170 | 0.026 | 0.096 | 0.155 | 0.005 | 0.051 | -0.006 | 0.230 |
| LPC 20:4 | 0.329 | 0.068 | 0.201 | 0.211 | 0.040 | 0.281 | 0.104 | 0.259 | -0.103 |
| PC 24:0 | 0.233 | 0.002 | 0.043 | 0.081 | -0.067 | 0.123 | -0.063 | 0.178 | -0.144 |
| PC 26:0 | 0.113 | 0.122 | 0.014 | -0.023 | 0.079 | 0.179 | 0.104 | 0.046 | -0.137 |
| PC 28:1 | -0.101 | 0.009 | -0.065 | -0.020 | -0.035 | -0.114 | -0.195 | -0.093 | -0.069 |
| PC 30:0 | -0.251 | -0.191 | -0.242 | -0.192 | -0.222 | -0.283 | -0.325 | -0.228 | -0.095 |
| PC 32:0 | -0.235 | -0.081 | -0.178 | -0.132 | -0.094 | -0.230 | -0.217 | -0.185 | 0.100 |
| PC 32:1 | 0.198 | 0.307 | 0.228 | 0.350 | 0.248 | 0.200 | 0.209 | 0.291 | 0.266 |
| PC 32:2 | -0.023 | -0.159 | -0.091 | -0.020 | -0.193 | -0.083 | -0.188 | -0.053 | -0.295 |
| PC 32:3 | -0.245 | -0.144 | -0.177 | -0.170 | -0.182 | -0.197 | -0.212 | -0.244 | -0.225 |
| PC 34:1 | -0.250 | -0.087 | -0.189 | -0.098 | -0.123 | -0.271 | -0.250 | -0.190 | -0.066 |
| PC 34:2 | -0.262 | -0.024 | -0.159 | -0.049 | -0.048 | -0.274 | -0.185 | -0.187 | 0.002 |
| PC 34:3 | -0.066 | 0.030 | -0.062 | 0.045 | -0.008 | -0.072 | -0.080 | -0.028 | 0.000 |
| PC 34:4 | -0.156 | -0.302 | -0.229 | -0.233 | -0.358 | -0.182 | -0.281 | -0.202 | -0.302 |
| PC 36:0 | **-0.489** | -0.371 | -0.442 | -0.412 | -0.374 | **-0.492** | **-0.483** | **-0.451** | -0.121 |
| PC 36:1 | -0.337 | -0.244 | -0.322 | -0.242 | -0.273 | -0.388 | -0.388 | -0.304 | -0.205 |
| PC 36:2 | -0.203 | 0.030 | -0.124 | -0.029 | 0.003 | -0.217 | -0.138 | -0.147 | 0.034 |
| PC 36:3 | -0.332 | -0.137 | -0.205 | -0.126 | -0.153 | -0.316 | -0.275 | -0.230 | 0.048 |
| PC 36:4 | -0.099 | 0.066 | -0.052 | 0.054 | 0.034 | -0.090 | -0.029 | -0.038 | 0.045 |
| PC 36:5 | -0.177 | -0.167 | -0.226 | -0.172 | -0.217 | -0.202 | -0.275 | -0.189 | -0.316 |
| PC 36:6 | -0.307 | -0.155 | -0.251 | -0.196 | -0.184 | -0.343 | -0.332 | -0.289 | -0.116 |
| PC 38:0 | -0.439 | -0.284 | -0.381 | -0.315 | -0.327 | -0.424 | -0.445 | -0.393 | -0.193 |
| PC 38:3 | -0.347 | -0.170 | -0.245 | -0.187 | -0.179 | -0.356 | -0.329 | -0.283 | 0.004 |
| PC 38:4 | -0.215 | 0.071 | -0.118 | 0.011 | 0.038 | -0.209 | -0.096 | -0.130 | 0.073 |
| PC 38:5 | -0.128 | 0.066 | -0.096 | -0.019 | 0.003 | -0.095 | -0.095 | -0.111 | -0.098 |
| PC 38:6 | **-0.559** | -0.331 | **-0.461** | -0.387 | -0.338 | **-0.588** | **-0.501** | **-0.501** | -0.181 |
| PC 40:1 | 0.054 | -0.183 | -0.175 | -0.234 | -0.291 | 0.053 | -0.162 | -0.020 | -0.299 |
| PC 40:2 | 0.134 | 0.302 | 0.085 | 0.217 | 0.229 | 0.084 | 0.146 | 0.120 | 0.133 |
| PC 40:3 | -0.341 | -0.185 | -0.272 | -0.168 | -0.215 | -0.355 | -0.314 | -0.267 | -0.153 |
| PC 40:4 | -0.344 | -0.134 | -0.251 | -0.142 | -0.130 | -0.338 | -0.274 | -0.257 | -0.054 |
| PC 40:5 | -0.075 | 0.087 | -0.070 | -0.012 | 0.040 | -0.065 | -0.047 | -0.070 | 0.173 |
| PC 40:6 | -0.429 | -0.298 | -0.367 | -0.328 | -0.306 | **-0.460** | -0.435 | -0.400 | -0.162 |
| PC 42:0 | 0.002 | -0.158 | -0.162 | -0.152 | -0.243 | -0.032 | -0.122 | -0.048 | 0.055 |
| PC 42:1 | **-0.528** | -0.180 | -0.410 | -0.287 | -0.203 | **-0.538** | -0.322 | -0.423 | -0.257 |
| PC 42:2 | 0.014 | -0.171 | -0.107 | -0.084 | -0.163 | -0.015 | -0.159 | -0.022 | -0.078 |
| PC 42:4 | **-0.690** | -0.383 | **-0.559** | -0.443 | -0.366 | **-0.674** | **-0.520** | **-0.584** | -0.295 |
| PC 42:5 | **-0.656** | -0.325 | **-0.561** | **-0.463** | -0.337 | **-0.653** | **-0.568** | **-0.596** | -0.355 |
| PC 42:6 | 0.137 | 0.024 | -0.002 | 0.001 | -0.073 | 0.144 | -0.018 | 0.061 | -0.182 |
| GPCe 30:0 | -0.223 | -0.074 | -0.231 | -0.231 | -0.124 | -0.180 | -0.218 | -0.273 | -0.313 |
| GPCe 30:1 | 0.014 | -0.036 | -0.073 | -0.031 | -0.071 | -0.068 | -0.153 | -0.075 | -0.225 |
| GPCe 30:2 | 0.005 | -0.153 | -0.074 | -0.071 | -0.172 | -0.051 | -0.177 | 0.004 | -0.106 |
| GPCe 32:1 | -0.298 | -0.014 | -0.120 | -0.028 | -0.014 | -0.275 | -0.152 | -0.182 | 0.170 |
| GPCe 32:2 | -0.370 | -0.152 | -0.302 | -0.190 | -0.178 | -0.371 | -0.308 | -0.302 | -0.101 |
| GPCe 34:0 | -0.084 | 0.149 | 0.014 | 0.072 | 0.130 | -0.066 | 0.014 | -0.022 | 0.170 |
| GPCe 34:1 | -0.208 | -0.017 | -0.098 | -0.020 | -0.058 | -0.218 | -0.170 | -0.139 | -0.014 |
| GPCe 34:2 | -0.215 | 0.071 | -0.053 | 0.043 | 0.044 | -0.185 | -0.105 | -0.108 | 0.170 |
| GPCe 34:3 | -0.308 | 0.017 | -0.187 | -0.059 | -0.008 | -0.241 | -0.105 | -0.206 | 0.100 |
| GPCe 36:0 | -0.314 | -0.171 | -0.289 | -0.218 | -0.236 | -0.331 | -0.326 | -0.302 | -0.261 |
| GPCe 36:1 | -0.217 | -0.107 | -0.224 | -0.139 | -0.156 | -0.235 | -0.235 | -0.203 | -0.208 |
| GPCe 36:2 | -0.254 | -0.038 | -0.158 | -0.036 | -0.062 | -0.248 | -0.183 | -0.176 | -0.026 |
| GPCe 36:3 | -0.150 | 0.134 | -0.036 | 0.087 | 0.104 | -0.132 | -0.021 | -0.058 | 0.193 |
| GPCe 36:4 | -0.047 | 0.236 | 0.041 | 0.169 | 0.212 | -0.011 | 0.089 | 0.027 | 0.233 |
| GPCe 36:5 | -0.162 | 0.159 | -0.015 | 0.144 | 0.115 | -0.137 | 0.020 | -0.047 | 0.089 |
| GPCe 38:0 | -0.203 | -0.134 | -0.205 | -0.141 | -0.190 | -0.214 | -0.251 | -0.182 | -0.180 |
| GPCe 38:1 | 0.011 | -0.165 | -0.110 | -0.087 | -0.257 | -0.015 | -0.195 | -0.044 | -0.322 |
| GPCe 38:2 | 0.220 | 0.418 | 0.253 | 0.319 | 0.369 | 0.271 | 0.299 | 0.260 | 0.150 |
| GPCe 38:3 | -0.129 | -0.041 | -0.102 | 0.008 | -0.074 | -0.143 | -0.140 | -0.072 | -0.098 |
| GPCe 38:4 | -0.292 | -0.033 | -0.206 | -0.079 | -0.057 | -0.265 | -0.209 | -0.211 | 0.017 |
| GPCe 38:5 | -0.242 | -0.017 | -0.144 | -0.024 | -0.038 | -0.202 | -0.152 | -0.150 | 0.021 |
| GPCe 38:6 | **-0.462** | -0.202 | -0.324 | -0.269 | -0.186 | **-0.453** | -0.373 | -0.386 | -0.023 |
| GPCe 40:1 | 0.221 | 0.074 | 0.078 | -0.005 | 0.081 | 0.202 | 0.089 | 0.115 | -0.102 |
| GPCe 40:2 | -0.165 | -0.144 | -0.187 | -0.165 | -0.172 | -0.198 | -0.254 | -0.187 | -0.140 |
| GPCe 40:3 | -0.238 | -0.099 | -0.234 | -0.129 | -0.122 | -0.266 | -0.180 | -0.193 | -0.202 |
| GPCe 40:4 | -0.062 | 0.027 | -0.108 | -0.055 | -0.014 | -0.044 | -0.062 | -0.093 | -0.064 |
| GPCe 40:5 | -0.361 | -0.150 | -0.273 | -0.148 | -0.183 | -0.365 | -0.284 | -0.282 | -0.230 |
| GPCe 40:6 | -0.301 | -0.180 | -0.197 | -0.167 | -0.169 | -0.343 | -0.311 | -0.253 | -0.021 |
| GPCe 42:0 | -0.120 | -0.153 | -0.290 | -0.256 | -0.190 | -0.128 | -0.170 | -0.201 | -0.329 |
| GPCe 42:1 | -0.065 | 0.036 | -0.093 | -0.098 | -0.044 | 0.048 | -0.051 | -0.065 | -0.173 |
| GPCe 42:2 | -0.063 | 0.029 | -0.084 | 0.002 | -0.041 | -0.065 | -0.090 | -0.026 | 0.111 |
| GPCe 42:3 | 0.000 | 0.235 | 0.047 | 0.147 | 0.189 | 0.015 | 0.051 | 0.035 | 0.060 |
| GPCe 42:4 | 0.018 | -0.121 | 0.010 | 0.045 | -0.146 | -0.012 | -0.186 | 0.052 | -0.260 |
| GPCe 42:5 | -0.322 | 0.089 | -0.251 | -0.157 | 0.039 | -0.271 | -0.098 | -0.271 | -0.096 |
| GPCe 44:3 | -0.162 | -0.039 | -0.165 | -0.090 | -0.117 | -0.165 | -0.203 | -0.149 | -0.075 |
| GPCe 44:4 | -0.197 | -0.062 | -0.187 | -0.110 | -0.150 | -0.179 | -0.099 | -0.139 | 0.032 |
| GPCe 44:5 | -0.021 | -0.026 | -0.081 | -0.058 | -0.071 | -0.012 | -0.042 | -0.111 | -0.271 |
| GPCe 44:6 | -0.111 | -0.035 | -0.133 | -0.057 | -0.071 | -0.041 | -0.029 | -0.074 | -0.218 |
| SM (OH) 14:1 | -0.208 | -0.146 | -0.186 | -0.114 | -0.176 | -0.262 | -0.266 | -0.189 | -0.204 |
| SM (OH) 16:1 | -0.256 | -0.165 | -0.216 | -0.148 | -0.196 | -0.326 | -0.319 | -0.239 | -0.204 |
| SM (OH) 22:1 | -0.104 | -0.006 | -0.062 | -0.002 | -0.042 | -0.132 | -0.143 | -0.082 | -0.106 |
| SM (OH) 22:2 | -0.269 | -0.168 | -0.234 | -0.155 | -0.198 | -0.325 | -0.323 | -0.239 | -0.182 |
| SM (OH) 24:1 | -0.197 | -0.072 | -0.138 | -0.064 | -0.110 | -0.229 | -0.235 | -0.162 | -0.134 |
| SM 16:0 | -0.214 | -0.197 | -0.213 | -0.182 | -0.232 | -0.281 | -0.340 | -0.210 | -0.133 |
| SM 16:1 | -0.283 | -0.192 | -0.233 | -0.189 | -0.212 | -0.347 | -0.332 | -0.257 | -0.145 |
| SM 18:0 | -0.317 | -0.256 | -0.306 | -0.255 | -0.289 | -0.362 | -0.412 | -0.303 | -0.236 |
| SM 18:1 | -0.355 | -0.186 | -0.264 | -0.205 | -0.199 | -0.405 | -0.343 | -0.304 | -0.073 |
| SM 20:2 | -0.364 | -0.164 | -0.293 | -0.196 | -0.195 | -0.371 | -0.341 | -0.311 | -0.144 |
| SM 22:3 | -0.155 | 0.080 | -0.101 | -0.045 | 0.130 | -0.147 | 0.069 | -0.076 | 0.194 |
| SM 24:0 | -0.211 | -0.110 | -0.178 | -0.105 | -0.152 | -0.223 | -0.275 | -0.169 | -0.070 |
| SM 24:1 | -0.235 | -0.212 | -0.233 | -0.184 | -0.229 | -0.284 | -0.352 | -0.225 | -0.180 |
| SM 26:0 | -0.068 | -0.023 | -0.015 | 0.038 | -0.062 | -0.054 | -0.128 | -0.005 | -0.008 |
| SM 26:1 | -0.310 | -0.183 | -0.193 | -0.127 | -0.182 | -0.319 | -0.298 | -0.222 | -0.034 |
