# Supplementary material for: Metabolomic profiling reveals correlations between spermiogram parameters and the metabolites present in human spermatozoa and seminal plasma
Source: PLoS One. 2019 Feb 20;14(2):e0211679. doi: 10.1371/journal.pone.0211679 (PMC6382115; doi:10.1371/journal.pone.0211679)
Supplement: S12 Table — Data are Spearman correlation rank coefficients. Significances are highlighted in bolt. (DOCX) [file pone.0211679.s013.docx]

|  | SM (OH) 14:1 | SM (OH) 16:1 | SM (OH) 22:1 | SM (OH) 22:2 | SM (OH) 24:1 | SM 16:0 | SM 16:1 | SM 18:0 | SM 18:1 | SM 20:2 | SM 22:3 | SM 24:0 | SM 24:1 | SM 26:0 | SM 26:1 |
| --- | --- | --- | --- | --- | --- | --- | --- | --- | --- | --- | --- | --- | --- | --- | --- |
| \| LPC 14:0 \| \| --- \| | 0.406 | 0.332 | 0.403 | 0.320 | 0.420 | 0.367 | 0.415 | 0.331 | 0.325 | 0.381 | **0.523** | 0.377 | 0.266 | -0.257 | 0.122 |
| LPC 16:0 | 0.356 | 0.309 | 0.061 | 0.273 | 0.221 | 0.344 | 0.325 | 0.322 | 0.338 | 0.246 | -0.013 | 0.287 | 0.311 | -0.038 | 0.311 |
| LPC 16:1 | 0.203 | 0.119 | -0.071 | 0.078 | 0.068 | 0.191 | 0.205 | 0.183 | 0.191 | 0.241 | 0.167 | 0.198 | 0.189 | -0.140 | 0.143 |
| LPC 17:0 | 0.298 | 0.203 | 0.088 | 0.219 | 0.191 | 0.263 | 0.244 | 0.230 | 0.227 | 0.260 | 0.148 | 0.266 | 0.238 | -0.150 | 0.195 |
| LPC 18:0 | 0.367 | 0.326 | 0.071 | 0.278 | 0.225 | 0.346 | 0.346 | 0.328 | 0.356 | 0.287 | 0.029 | 0.296 | 0.320 | -0.023 | 0.323 |
| LPC 18:1 | 0.272 | 0.189 | -0.041 | 0.163 | 0.133 | 0.241 | 0.250 | 0.223 | 0.233 | 0.158 | -0.005 | 0.218 | 0.224 | -0.084 | 0.238 |
| LPC 18:2 | 0.361 | 0.278 | 0.123 | 0.287 | 0.260 | 0.365 | 0.365 | 0.347 | 0.356 | **0.452** | 0.053 | 0.370 | 0.353 | 0.258 | 0.362 |
| LPC 20:3 | 0.242 | 0.206 | 0.062 | 0.307 | 0.135 | 0.223 | 0.305 | 0.223 | 0.302 | 0.357 | -0.142 | 0.251 | 0.271 | 0.347 | 0.250 |
| LPC 20:4 | 0.137 | 0.033 | 0.014 | -0.044 | 0.047 | 0.135 | 0.096 | 0.111 | 0.038 | 0.311 | -0.008 | 0.167 | 0.017 | -0.028 | 0.027 |
| PC 24:0 | 0.179 | 0.055 | 0.013 | -0.049 | 0.136 | 0.174 | 0.135 | 0.159 | 0.041 | -0.115 | 0.022 | 0.194 | 0.071 | -0.087 | 0.112 |
| PC 26:0 | 0.056 | -0.060 | 0.092 | 0.049 | 0.169 | 0.063 | 0.092 | 0.072 | 0.029 | -0.071 | 0.301 | 0.162 | 0.105 | -0.293 | -0.047 |
| PC 28:1 | 0.373 | 0.301 | 0.054 | 0.198 | 0.230 | 0.370 | 0.325 | 0.334 | 0.331 | 0.217 | 0.002 | 0.304 | 0.262 | -0.134 | 0.320 |
| PC 30:0 | 0.167 | 0.115 | -0.162 | 0.048 | 0.015 | 0.150 | 0.131 | 0.117 | 0.143 | 0.065 | -0.088 | 0.072 | 0.111 | -0.078 | 0.273 |
| PC 32:0 | 0.185 | 0.175 | -0.071 | 0.187 | 0.060 | 0.168 | 0.170 | 0.134 | 0.197 | 0.165 | -0.117 | 0.084 | 0.183 | 0.009 | 0.299 |
| PC 32:1 | **0.475** | 0.379 | 0.245 | 0.414 | 0.389 | 0.438 | **0.454** | 0.423 | 0.386 | 0.324 | -0.055 | **0.462** | 0.442 | 0.420 | **0.458** |
| PC 32:2 | -0.014 | -0.049 | -0.233 | -0.142 | -0.159 | -0.026 | -0.072 | -0.047 | -0.069 | 0.064 | -0.404 | -0.011 | -0.102 | 0.213 | 0.038 |
| PC 32:3 | 0.018 | -0.017 | -0.174 | 0.055 | -0.084 | -0.002 | 0.035 | 0.000 | 0.056 | 0.094 | -0.164 | -0.009 | 0.029 | -0.168 | 0.016 |
| PC 34:1 | 0.335 | 0.333 | -0.078 | 0.226 | 0.160 | 0.317 | 0.335 | 0.317 | 0.359 | 0.216 | -0.099 | 0.238 | 0.328 | 0.089 | 0.397 |
| PC 34:2 | 0.311 | 0.255 | -0.020 | 0.236 | 0.132 | 0.245 | 0.308 | 0.227 | 0.296 | 0.262 | -0.058 | 0.220 | 0.247 | 0.006 | 0.347 |
| PC 34:3 | 0.329 | 0.234 | 0.013 | 0.193 | 0.172 | 0.293 | 0.314 | 0.269 | 0.299 | 0.329 | 0.081 | 0.289 | 0.263 | 0.089 | 0.287 |
| PC 34:4 | -0.095 | -0.223 | -0.263 | -0.202 | -0.199 | -0.134 | -0.152 | -0.171 | -0.164 | -0.057 | 0.101 | -0.129 | -0.183 | -0.269 | -0.096 |
| PC 36:0 | -0.208 | -0.167 | -0.360 | -0.126 | -0.270 | -0.185 | -0.191 | -0.205 | -0.146 | -0.311 | -0.165 | -0.284 | -0.159 | 0.203 | -0.011 |
| PC 36:1 | 0.191 | 0.183 | -0.210 | 0.057 | 0.012 | 0.168 | 0.198 | 0.168 | 0.221 | 0.068 | -0.092 | 0.083 | 0.165 | -0.057 | 0.288 |
| PC 36:2 | 0.334 | 0.285 | 0.035 | 0.267 | 0.182 | 0.287 | 0.325 | 0.263 | 0.316 | 0.297 | -0.031 | 0.250 | 0.280 | -0.027 | 0.361 |
| PC 36:3 | 0.226 | 0.235 | -0.148 | 0.205 | 0.048 | 0.197 | 0.214 | 0.182 | 0.251 | 0.163 | -0.265 | 0.110 | 0.233 | 0.211 | 0.397 |
| PC 36:4 | 0.278 | 0.198 | 0.017 | 0.222 | 0.126 | 0.209 | 0.286 | 0.194 | 0.250 | 0.372 | -0.065 | 0.244 | 0.226 | 0.063 | 0.300 |
| PC 36:5 | 0.080 | 0.026 | -0.207 | -0.037 | -0.034 | 0.060 | 0.084 | 0.062 | 0.057 | 0.025 | -0.212 | 0.066 | 0.032 | -0.100 | 0.065 |
| PC 36:6 | 0.215 | 0.158 | -0.143 | 0.141 | 0.023 | 0.161 | 0.206 | 0.132 | 0.214 | 0.084 | -0.139 | 0.107 | 0.158 | -0.046 | 0.293 |
| PC 38:0 | 0.063 | 0.060 | -0.254 | 0.022 | -0.098 | 0.065 | 0.080 | 0.063 | 0.143 | -0.015 | -0.173 | -0.014 | 0.068 | -0.082 | 0.171 |
| PC 38:3 | 0.156 | 0.153 | -0.139 | 0.111 | -0.003 | 0.131 | 0.134 | 0.102 | 0.164 | 0.084 | -0.156 | 0.041 | 0.110 | 0.000 | 0.238 |
| PC 38:4 | 0.320 | 0.279 | 0.050 | 0.282 | 0.169 | 0.275 | 0.323 | 0.257 | 0.317 | 0.353 | -0.098 | 0.259 | 0.275 | 0.069 | 0.374 |
| PC 38:5 | 0.310 | 0.217 | 0.016 | 0.254 | 0.185 | 0.292 | 0.304 | 0.269 | 0.305 | 0.280 | -0.098 | 0.293 | 0.287 | 0.038 | 0.342 |
| PC 38:6 | 0.065 | 0.133 | -0.315 | 0.088 | -0.119 | 0.060 | 0.104 | 0.059 | 0.189 | -0.018 | -0.286 | -0.090 | 0.120 | 0.157 | 0.323 |
| PC 40:1 | -0.153 | -0.249 | -0.145 | -0.287 | -0.077 | -0.128 | -0.161 | -0.119 | -0.211 | -0.301 | 0.140 | -0.056 | -0.170 | -0.314 | -0.152 |
| PC 40:2 | 0.371 | 0.300 | 0.252 | 0.384 | 0.245 | 0.359 | 0.442 | 0.367 | 0.441 | 0.311 | 0.037 | 0.394 | 0.394 | 0.187 | 0.338 |
| PC 40:3 | 0.170 | 0.130 | -0.205 | 0.075 | 0.016 | 0.150 | 0.134 | 0.123 | 0.144 | 0.099 | -0.221 | 0.075 | 0.128 | 0.194 | 0.346 |
| PC 40:4 | 0.183 | 0.168 | -0.140 | 0.138 | 0.035 | 0.138 | 0.188 | 0.120 | 0.188 | 0.195 | -0.164 | 0.083 | 0.147 | 0.022 | 0.302 |
| PC 40:5 | 0.238 | 0.138 | 0.073 | 0.232 | 0.099 | 0.198 | 0.239 | 0.155 | 0.236 | 0.321 | 0.105 | 0.217 | 0.203 | -0.017 | 0.269 |
| PC 40:6 | 0.018 | 0.003 | -0.257 | -0.035 | -0.115 | 0.006 | -0.017 | -0.033 | 0.020 | -0.070 | -0.120 | -0.105 | -0.033 | -0.051 | 0.124 |
| PC 42:0 | -0.134 | -0.267 | -0.123 | -0.118 | -0.212 | -0.171 | -0.158 | -0.209 | -0.155 | -0.103 | 0.142 | -0.116 | -0.137 | -0.205 | -0.058 |
| PC 42:1 | 0.158 | 0.189 | -0.221 | 0.133 | 0.017 | 0.092 | 0.233 | 0.131 | 0.253 | -0.017 | -0.223 | 0.071 | 0.205 | 0.329 | 0.311 |
| PC 42:2 | -0.020 | -0.096 | -0.243 | -0.132 | -0.107 | -0.008 | -0.054 | -0.041 | -0.072 | 0.023 | -0.145 | -0.032 | -0.027 | 0.008 | 0.124 |
| PC 42:4 | -0.068 | 0.009 | -0.422 | -0.026 | -0.206 | -0.089 | -0.026 | -0.083 | 0.039 | -0.057 | -0.438 | -0.188 | -0.012 | 0.219 | 0.244 |
| PC 42:5 | 0.021 | 0.102 | -0.352 | 0.066 | -0.120 | 0.009 | 0.087 | 0.015 | 0.144 | -0.072 | -0.417 | -0.089 | 0.062 | 0.185 | 0.201 |
| PC 42:6 | 0.158 | 0.058 | -0.025 | 0.113 | 0.110 | 0.155 | 0.137 | 0.149 | 0.125 | 0.069 | -0.079 | 0.218 | 0.206 | -0.097 | 0.234 |
| GPCe 30:0 | 0.048 | -0.047 | -0.095 | 0.053 | 0.014 | 0.026 | 0.074 | 0.018 | 0.063 | -0.084 | -0.049 | 0.068 | 0.047 | -0.343 | 0.007 |
| GPCe 30:1 | 0.097 | 0.021 | -0.077 | 0.012 | -0.028 | 0.059 | 0.127 | 0.056 | 0.082 | -0.021 | -0.042 | 0.121 | 0.047 | 0.046 | 0.102 |
| GPCe 30:2 | 0.215 | 0.073 | -0.135 | -0.047 | 0.118 | 0.149 | 0.122 | 0.096 | 0.029 | 0.032 | 0.141 | 0.143 | 0.080 | -0.004 | 0.244 |
| GPCe 32:1 | 0.260 | 0.258 | 0.002 | 0.278 | 0.106 | 0.211 | 0.241 | 0.183 | 0.268 | 0.253 | -0.103 | 0.141 | 0.227 | 0.139 | 0.300 |
| GPCe 32:2 | 0.146 | 0.096 | -0.149 | 0.102 | -0.016 | 0.083 | 0.135 | 0.056 | 0.140 | 0.124 | -0.176 | 0.065 | 0.087 | -0.044 | 0.238 |
| GPCe 34:0 | 0.379 | 0.371 | 0.163 | 0.360 | 0.294 | 0.364 | 0.346 | 0.332 | 0.359 | 0.388 | 0.034 | 0.293 | 0.353 | 0.044 | 0.343 |
| GPCe 34:1 | 0.298 | 0.254 | 0.032 | 0.201 | 0.160 | 0.259 | 0.248 | 0.235 | 0.247 | 0.207 | -0.027 | 0.211 | 0.200 | -0.085 | 0.230 |
| GPCe 34:2 | 0.392 | 0.369 | 0.094 | 0.361 | 0.240 | 0.356 | 0.373 | 0.335 | 0.391 | 0.316 | -0.013 | 0.296 | 0.353 | 0.118 | 0.370 |
| GPCe 34:3 | 0.170 | 0.150 | 0.014 | 0.247 | 0.033 | 0.095 | 0.223 | 0.099 | 0.244 | 0.318 | -0.036 | 0.140 | 0.177 | -0.082 | 0.173 |
| GPCe 36:0 | 0.203 | 0.157 | -0.157 | 0.136 | 0.026 | 0.159 | 0.251 | 0.182 | 0.271 | 0.033 | -0.079 | 0.144 | 0.215 | -0.146 | 0.235 |
| GPCe 36:1 | 0.245 | 0.182 | -0.114 | 0.134 | 0.087 | 0.209 | 0.265 | 0.211 | 0.269 | 0.161 | -0.016 | 0.191 | 0.227 | -0.055 | 0.247 |
| GPCe 36:2 | 0.334 | 0.255 | -0.047 | 0.245 | 0.142 | 0.268 | 0.343 | 0.257 | 0.340 | 0.232 | 0.007 | 0.253 | 0.292 | 0.110 | 0.330 |
| GPCe 36:3 | 0.403 | 0.370 | 0.108 | 0.392 | 0.229 | 0.350 | 0.414 | 0.335 | 0.423 | 0.442 | -0.045 | 0.332 | 0.388 | 0.181 | 0.422 |
| GPCe 36:4 | 0.405 | 0.382 | 0.204 | 0.418 | 0.279 | 0.386 | 0.436 | 0.374 | 0.450 | **0.527** | 0.100 | 0.371 | 0.403 | 0.201 | 0.311 |
| GPCe 36:5 | 0.417 | 0.374 | 0.142 | 0.398 | 0.250 | 0.352 | 0.418 | 0.347 | 0.424 | **0.469** | -0.095 | 0.355 | 0.379 | 0.137 | 0.437 |
| GPCe 38:0 | 0.195 | 0.111 | -0.139 | 0.067 | 0.061 | 0.176 | 0.174 | 0.162 | 0.167 | 0.075 | -0.076 | 0.149 | 0.156 | -0.081 | 0.255 |
| GPCe 38:1 | -0.118 | -0.188 | -0.176 | -0.174 | -0.171 | -0.099 | -0.170 | -0.112 | -0.146 | -0.231 | -0.285 | -0.074 | -0.170 | -0.005 | -0.183 |
| GPCe 38:2 | **0.567** | **0.461** | 0.356 | 0.438 | **0.511** | **0.532** | **0.552** | **0.513** | **0.499** | **0.569** | 0.126 | **0.589** | **0.495** | 0.200 | 0.437 |
| GPCe 38:3 | 0.242 | 0.126 | -0.033 | 0.063 | 0.094 | 0.167 | 0.195 | 0.138 | 0.146 | 0.219 | 0.005 | 0.202 | 0.092 | -0.073 | 0.149 |
| GPCe 38:4 | 0.307 | 0.287 | -0.096 | 0.288 | 0.117 | 0.280 | 0.343 | 0.272 | 0.373 | 0.285 | -0.160 | 0.238 | 0.338 | 0.342 | 0.411 |
| GPCe 38:5 | 0.325 | 0.314 | -0.041 | 0.263 | 0.176 | 0.316 | 0.331 | 0.308 | 0.356 | 0.360 | 0.001 | 0.262 | 0.334 | 0.245 | 0.375 |
| GPCe 38:6 | 0.060 | 0.128 | -0.151 | 0.087 | -0.045 | 0.062 | 0.066 | 0.042 | 0.122 | 0.049 | -0.121 | -0.065 | 0.044 | -0.002 | 0.111 |
| GPCe 40:1 | -0.047 | -0.097 | 0.022 | -0.124 | 0.023 | 0.012 | -0.053 | -0.011 | -0.048 | -0.121 | 0.007 | -0.005 | -0.071 | 0.059 | -0.144 |
| GPCe 40:2 | 0.212 | 0.122 | -0.142 | 0.074 | 0.055 | 0.185 | 0.195 | 0.156 | 0.198 | 0.081 | 0.056 | 0.134 | 0.168 | -0.041 | 0.216 |
| GPCe 40:3 | 0.155 | 0.083 | -0.121 | 0.027 | 0.043 | 0.086 | 0.129 | 0.062 | 0.096 | 0.158 | -0.067 | 0.098 | 0.065 | 0.038 | 0.175 |
| GPCe 40:4 | 0.065 | -0.081 | 0.004 | 0.068 | 0.031 | 0.039 | 0.039 | -0.009 | 0.008 | 0.053 | 0.104 | 0.084 | 0.020 | -0.219 | -0.015 |
| GPCe 40:5 | 0.236 | 0.181 | -0.178 | 0.102 | 0.066 | 0.183 | 0.227 | 0.176 | 0.217 | 0.169 | -0.087 | 0.164 | 0.176 | 0.203 | 0.288 |
| GPCe 40:6 | 0.114 | 0.136 | -0.134 | 0.050 | -0.005 | 0.105 | 0.057 | 0.065 | 0.074 | 0.044 | -0.095 | -0.021 | 0.033 | 0.046 | 0.124 |
| GPCe 42:0 | -0.134 | -0.259 | -0.118 | -0.168 | -0.090 | -0.186 | -0.125 | -0.202 | -0.173 | -0.199 | 0.289 | -0.074 | -0.147 | **-0.505** | -0.179 |
| GPCe 42:1 | 0.117 | 0.023 | 0.007 | 0.050 | 0.148 | 0.167 | 0.111 | 0.161 | 0.122 | 0.081 | 0.006 | 0.192 | 0.131 | -0.169 | 0.150 |
| GPCe 42:2 | 0.256 | 0.175 | 0.049 | 0.220 | 0.117 | 0.205 | 0.275 | 0.198 | 0.278 | 0.125 | 0.091 | 0.232 | 0.241 | 0.029 | 0.143 |
| GPCe 42:3 | **0.496** | 0.363 | 0.221 | 0.405 | 0.392 | 0.444 | **0.502** | 0.417 | 0.444 | 0.358 | 0.226 | **0.465** | 0.426 | 0.060 | 0.361 |
| GPCe 42:4 | 0.283 | 0.227 | -0.156 | -0.020 | 0.140 | 0.253 | 0.226 | 0.247 | 0.156 | 0.160 | -0.167 | 0.238 | 0.133 | 0.270 | 0.199 |
| GPCe 42:5 | 0.289 | 0.226 | 0.059 | 0.281 | 0.270 | 0.260 | 0.290 | 0.248 | 0.311 | 0.011 | 0.063 | 0.286 | 0.343 | 0.190 | 0.379 |
| GPCe 44:3 | 0.271 | 0.211 | -0.037 | 0.216 | 0.118 | 0.269 | 0.275 | 0.262 | 0.316 | 0.121 | -0.089 | 0.227 | 0.283 | -0.011 | 0.297 |
| GPCe 44:4 | -0.071 | -0.121 | -0.055 | 0.097 | -0.106 | -0.140 | -0.035 | -0.123 | -0.024 | -0.284 | -0.085 | -0.051 | 0.009 | 0.156 | -0.029 |
| GPCe 44:5 | -0.123 | -0.208 | -0.096 | -0.050 | -0.132 | -0.098 | -0.126 | -0.117 | -0.102 | -0.085 | -0.021 | -0.054 | -0.095 | 0.062 | -0.162 |
| GPCe 44:6 | 0.026 | -0.084 | -0.068 | -0.046 | 0.044 | -0.003 | 0.008 | -0.003 | -0.038 | 0.084 | 0.027 | 0.086 | 0.008 | -0.286 | 0.055 |
| SM (OH) 14:1 | 0.250 | 0.239 | -0.123 | 0.093 | 0.087 | 0.236 | 0.227 | 0.229 | 0.238 | 0.201 | -0.048 | 0.153 | 0.189 | -0.021 | 0.241 |
| SM (OH) 16:1 | 0.244 | 0.229 | -0.114 | 0.096 | 0.068 | 0.215 | 0.226 | 0.206 | 0.233 | 0.124 | -0.044 | 0.129 | 0.162 | -0.112 | 0.214 |
| SM (OH) 22:1 | 0.365 | 0.299 | 0.020 | 0.193 | 0.204 | 0.314 | 0.347 | 0.304 | 0.320 | 0.288 | 0.072 | 0.293 | 0.265 | -0.118 | 0.251 |
| SM (OH) 22:2 | 0.272 | 0.270 | -0.139 | 0.130 | 0.087 | 0.250 | 0.274 | 0.248 | 0.284 | 0.180 | -0.062 | 0.161 | 0.227 | -0.012 | 0.308 |
| SM (OH) 24:1 | 0.341 | 0.299 | -0.050 | 0.196 | 0.162 | 0.299 | 0.329 | 0.292 | 0.319 | 0.230 | -0.030 | 0.251 | 0.268 | -0.048 | 0.297 |
| SM 16:0 | 0.217 | 0.186 | -0.143 | 0.062 | 0.052 | 0.203 | 0.185 | 0.182 | 0.195 | 0.038 | -0.045 | 0.102 | 0.152 | -0.095 | 0.253 |
| SM 16:1 | 0.183 | 0.193 | -0.148 | 0.082 | 0.021 | 0.156 | 0.177 | 0.149 | 0.185 | 0.088 | -0.114 | 0.057 | 0.128 | -0.098 | 0.229 |
| SM 18:0 | 0.194 | 0.181 | -0.209 | 0.038 | 0.025 | 0.188 | 0.185 | 0.180 | 0.212 | 0.043 | -0.071 | 0.087 | 0.153 | -0.102 | 0.264 |
| SM 18:1 | 0.168 | 0.197 | -0.126 | 0.119 | 0.005 | 0.143 | 0.173 | 0.134 | 0.206 | 0.086 | -0.117 | 0.029 | 0.129 | -0.070 | 0.249 |
| SM 20:2 | 0.189 | 0.226 | -0.215 | 0.212 | -0.008 | 0.165 | 0.253 | 0.191 | 0.299 | 0.090 | -0.443 | 0.105 | 0.271 | 0.096 | 0.417 |
| SM 22:3 | 0.144 | 0.236 | 0.104 | 0.213 | 0.166 | 0.098 | 0.190 | 0.107 | 0.192 | 0.289 | 0.120 | 0.072 | 0.218 | 0.006 | 0.331 |
| SM 24:0 | 0.319 | 0.278 | -0.111 | 0.171 | 0.125 | 0.301 | 0.316 | 0.293 | 0.329 | 0.195 | -0.088 | 0.244 | 0.287 | 0.072 | 0.377 |
| SM 24:1 | 0.223 | 0.193 | -0.195 | 0.053 | 0.038 | 0.200 | 0.217 | 0.188 | 0.217 | 0.116 | -0.080 | 0.120 | 0.165 | -0.057 | 0.278 |
| SM 26:0 | 0.290 | 0.265 | -0.061 | 0.136 | 0.138 | 0.287 | 0.245 | 0.272 | 0.238 | 0.324 | -0.247 | 0.245 | 0.221 | 0.158 | 0.357 |
| SM 26:1 | 0.198 | 0.208 | -0.203 | 0.117 | 0.019 | 0.176 | 0.168 | 0.155 | 0.189 | 0.157 | -0.283 | 0.071 | 0.167 | 0.255 | 0.377 |
